# Supplementary material for: Cross-sectional study comparing cognitive function in treatment responsive versus treatment non-responsive schizophrenia: evidence from the STRATA study
Source: BMJ Open. 2021 Nov 25;11(11):e054160. doi: 10.1136/bmjopen-2021-054160 (PMC8627394; doi:10.1136/bmjopen-2021-054160)
Supplement: Supplementary data [file bmjopen-2021-054160supp001.pdf]

STRATA: Investigating factors associated with response to antipsychotic treatment

Sponsor

Name of organisation: King's College London
Address: Room 1.8 Hodgkin Building, Guy's Campus, London SE1 4UL
Telephone: 020 7848 6960
Fax:
Email: keith.brennan@kcl.ac.uk

Chief Investigator

Name: Dr James MacCabe
Address: Institute of Psychiatry, Psychology & Neuroscience (IoPPN), De Crespigny Park, SE5 8AF
Telephone: 02078480757
Fax:
Email: james.maccabe@kcl.ac.uk

Name and address of Principal Investigator (on grant award).

Name: Prof Shitij Kapur
Address: Institute of Psychiatry, Psychology & Neuroscience (IoPPN), De Crespigny Park, SE5 8AF
Telephone: 020 7848 0593
Fax:
Email: shitij.kapur@kcl.ac.uk

Name and address of lead Co-Investigators at sites

Name: Prof Shon Lewis
Address: The University of Manchester, University Place, Oxford Road, Manchester, M13 9PL
Telephone: 0161 306 7944
Fax:
Email: shon.lewis@manchester.ac.uk

Name: Prof Mick O'Donovan
Address: MRC Centre for Neuropsychiatric Genetics and Genomics, Cardiff University, Henry Wellcome Building, Heath Park, Cardiff, CF14 4XN
Telephone: (029) 20687066
Fax:
Email: odonovanmc@cardiff.ac.uk

Millgate E, *et al. BMJ Open* 2021; 11:e054160. doi: 10.1136/bmjopen-2021-054160

BMJ Publishing Group Limited (BMJ) disclaims all liability and responsibility arising from any reliance placed on this supplemental material which has been supplied by the author(s)

STRATA: Investigating factors associated with response to antipsychotic treatment
Protocol. Version 4.0, 19th August 2016
REC REF: 15/LO/0038

Name: Prof Stephen Lawrie
Address: University of Edinburgh, Royal Edinburgh Hospital, Edinburgh, EH10 5HF
Telephone: 0131 537 6671
Fax:
Email: s.lawrie@ed.ac.uk

Millgate E, *et al. BMJ Open* 2021; 11:e054160. doi: 10.1136/bmjopen-2021-054160

STRATA: Investigating factors associated with response to antipsychotic treatment
Protocol. Version 4.0, 19th August 2016
REC REF: 15/LO/0038

CONTENTS

1. Introduction

2. Study Objectives and Design

2.1. Study Objectives

2.2 Study Design

3. Sample Size, Statistics, Selection and Withdrawal of Subjects

4. Study procedures

5. Sample Handling and Laboratories

6. Assessment of Safety

7. Study oversight arrangements

8. Ethics and Regulatory Approvals

9. Data Handling

10. Finance & Publication Policy

STRATA: Investigating factors associated with response to antipsychotic treatment
Protocol. Version 4.0, 19th August 2016
REC REF: 15/LO/0038

Study Synopsis

Full Title	Investigating factors associated with response to antipsychotic treatment
Short Title/Acronym	STRATA
Protocol Version number and Date	Version 4.0, 19th August 2016
Study Duration	36 months
Study Design	Basic Science
Sponsor/Co-sponsors	Kings College London / South London and Maudsley NHS Foundation Trust
Chief Investigator	Dr James MacCabe
REC number	15/LO/0038
Primary objective	The principle objective is to use Positron Emission Tomography (PET), Magnetic Resonance Spectroscopy (MRS), genetic and clinical data to confirm recent evidence of a distinct subtype of schizophrenia, based on differences in dopamine and glutamate function which would lead to developing a clinically useful, acceptable and cost-effective stratification tool.
Secondary objective (s)	To establish a lasting network of academia and industry partners and patients databases to facilitate and expedite both follow-up and novel research built to address patient stratification.<br><br>To examine and improve test-re-test and intercentre reliability of neuroimaging procedures for future large scale multisite studies in this mould that will be conducted.
Number of Subject	100
Main Inclusion Criteria	aged 18-65;<br><br>DSM 5 schizophrenia/schizophreniform disorder.

Statistical Methodology and Analysis	Summary statistics will be used to describe the demographic and clinical characteristics of each participant group.<br><br>Group differences in demographic, clinical variables and 18F-DOPAKi and glutamate concentration will be determined using pre-specified between group comparisons as appropriate (e.g. Chi square; Fischer's Exact; ANOVA).

STRATA: Investigating factors associated with response to antipsychotic treatment
Protocol. Version 4.0, 19th August 2016
REC REF: 15/LO/0038

1. Introduction

People with schizophrenia suffer from a range of symptoms including hallucinations (such as hearing voices), delusions (false beliefs) and thought disorder (thoughts not flowing in a logical way), as well as 'negative symptoms' such as a lack of motivation and withdrawal from social contact. Currently, antipsychotic medication is the mainstay of treatment for schizophrenia and all existing antipsychotic medications are thought to work by acting to reduce transmission of a brain chemical called dopamine. However, even after attempts to treat the disorder with two different antipsychotics, around 30% of patients still fail to improve. When this happens, the medical guidelines recommend treatment with a different drug called clozapine. However clozapine has several side effects and requires regular blood tests, so people do not like taking it. It is also ineffective in some patients.

The result is that a large number of patients spend too long on ineffective drugs which impact greatly on their mental health, well-being and quality of life whilst the cost of ineffective treatment is a huge financial burden to the NHS, consuming 25-50% of the total national mental health budget.

STRATA (funded by a £5M Medical Research Council award) aims to build on new evidence from neuroimaging and genetics studies suggesting that those who do not respond may actually have a completely different neurochemical abnormality causing their symptoms (the same sort of symptoms as are caused by excessive dopamine), involving a different chemical called glutamate. There are some new medicines under development that we hope will help people whose illness has not responded to standard medicines acting on dopamine.

We aim to develop a method to predict, even as early as when first seen, which patients will respond to standard dopamine drugs, and which people are instead more likely to respond to the new glutamate drugs. This will allow people to receive the medicines they need straight away, without having to try ineffective drugs first.

The proposed research programme is broken down into several parts. This protocol describes the first study, which is a UK, multicentre study using brain scans to confirm that those patients who don't respond to standard treatments have higher glutamate levels, but lower dopamine levels than those who respond well. This information, along with clinical and genetic information, will be used to develop tests to identify in advance which people will respond to dopaminergic versus glutamatergic medication.

2. Study Objectives and Design

2.1. Study Objectives and Outcomes

The principle objective is to use Positron Emission Tomography (PET), Magnetic Resonance Spectroscopy (MRS), genetic and clinical data to confirm recent evidence of a distinct subtype of schizophrenia, based on differences in dopamine and glutamate function which would lead to developing a clinically useful, acceptable and cost-effective stratification tool.

The secondary research objectives are:

i) To establish a lasting network of academia and industry partners and patient databases to facilitate and expedite both follow-up and novel research built to address patient stratification.

ii) To examine and improve test-re-test and intercentre reliability of neuroimaging procedures for future large scale multisite studies in this mould that will be conducted.

The study is designed to generate a predictive test for treatment response so the outcome will be the overall measure generated. The data that will lead to this will include MRS glutamate level, the PET Ki value, polygenic risk score and clinical variables such as PANSS score.

2.2 Study Design

STRATA is a multi centred study. 100 participants will be recruited across 4 university research sites including KCL, University of Manchester, Cardiff University, and University of Edinburgh.

Participants will consent to all aspects of the study including interviews/assessments, blood and urine sampling, MRI scan and PET scan (the latter in London and Manchester only) but can also choose to opt out of some tasks if necessary.

1. Assessments

An initial interview will collect demographic and personal information (e.g. address, contact details, date of birth, gender, handedness, head injury and other relevant medical history), and structured assessments of medication history and response. Clinical information will also be recorded from medical records. The Mini International Neuropsychiatric Interview (MINI) will be used to confirm diagnosis, which takes around 15 minutes to complete.

Illness severity will be measured using:

i. Positive and Negative Syndrome Scale (PANSS),
ii. Clinical Global Impression scale (CGI -SCH)
iii. Kemp Clinician Rating Scale (of adherence to treatment)
iv. Brief Assessment of Cognition in Schizophrenia (BACS)

2. Biological samples

Blood samples will be collected via cannula (as described under the PET scan section below) or by venous puncture, during a routine blood sample whenever possible. The participant will give up to 50ml in blood (around 3 tablespoons), this is in line with sampling guidelines.

While the biological sample collection is ongoing, samples will be stored at the laboratory corresponding to each research site. The samples for genetics analysis will subsequently be transferred to the MRC Center for Neuropsychiatric Genetics and Genomics, Cardiff University.

KCL/South London and the Maudsley (SLaM) participants will also be invited to participate in the BRC Biobank. This is covered by a separate ethical approval (09/H0606/84 NRES Committee South Central-Oxford C.)

Participants will be asked to provide samples (urine and blood) for metabolomics analysis. This will be processed at MRC-NIHR National Phenome Centre. The Centre is funded by the MRC and NIHR and led by Imperial College London and King's College London.

As of April 2016, participants will also have a sample taken for proteomic analysis. These samples will be sent to the University of Manchester (Molecular Pathology Innovation Centre). This will be within the 50ml sampling guideline already approved.

3. **Magnetic Resonance Imaging (MRI)**

The MRI scans (100 in total) will take place at four locations (London, Cardiff, Manchester and Edinburgh) at NHS Trust or University sites. The MRI session will last a maximum of 1 hour. During the scan, participants will be asked to lie flat on their back with their head inside the scanner. The scanner makes a loud noise as it takes pictures, so participants will be given headphones to wear and asked to lie as still as possible. The researcher will be able to speak to the participant over the microphone throughout and participants will be told if they do feel uncomfortable the session can be stopped at any time. The MRI scan itself is painless and safe. Some people find scans claustrophobic or anxiety-provoking, and we have a mock scanner that participants can try out first. The scanner consists of a powerful magnet, which may attract metal objects. Therefore before the scan participants will go through a safety questionnaire, to check that they can have the scan. If a participant has any metal in their body, either from accidents or operations, they may not be able to have the MRI scan, but they can still take part in the rest of the study.

All data collection will occur at 3 Tesla. During the scan, data acquisition will include acquisition of localizer, T1-weighted and T2-weighted structural scans. 1H-MRS data for measurement of regional concentrations of glutamate and other metabolites present in the 1H-MRS spectra will be acquired using conventional PRESS (Point RESolved Spectrocopy) acquisition routines, as well as a resting state fMRI sequence if time allows.

Due to change of scanner at Cardiff University, participants recruited in Cardiff prior to the decommissioning of the old scanner will be re-contacted and asked whether they would volunteer for a second MRI scan on the new scanner. They will also be asked to repeat some of the interview/assessments and may be asked for biological samples (only in circumstances where these were not provided previously). Participants will be reimbursed for their time at the same rate.

In the unlikely event that MRI scanner issues or excessive movement make the MRS data unusable at other sites, participants can be re-contacted and asked whether they would like to volunteer for a second scan.

4. **Positron Emission Tomography (PET)**

The PET scans (60 in total, subset of those having MRI scans) will take place at two sites:
i) Imanova Limited, Imperial College London, Hammersmith Hospital in London.
ii)The Wolfson Molecular Imaging Centre in Manchester.

PET with the radiotracer 18F-DOPA will be used to assess brain dopaminergic function in a sub-set of participants (N=60) recruited at KCL and University of Manchester. The PET scan procedure involves an initial transmission scan followed by a dynamic scan lasting approximately

90 minutes after injection of the radiotracer 18F DOPA through a cannula inserted into an arm vein. In the event the participant has to get off the scanner e.g. to go to the toilet or for some other reason then the transmission scan may be repeated to reposition them in the camera. In the unlikely event of technical failure prior to or during the PET scan the subject will be invited back for a replacement session (the total dose will then be ~7.5mSv, and the risks of this will be explained).

In Manchester, participants will be offered the option of having an extra, High Resolution Research Tomograph (HRRT) scan after their main STRATA PET scan (after a 15 minute comfort break). This will be between 30-60 minutes depending upon participant tolerability. Due to the long half-life of 18F and the slow removal of 18F from the brain, this extra scan will not involve any further injection of radiotracer. Another transmission scan will be carried out for attenuation correction purposes although this will be of very low radiation dose (0.02mSv). In the event of significant head movement during the HRRT scan, this transmission scan may be repeated.

In order to minimise the peripheral breakdown of 18F-DOPA, an oral dose of 150mg carbidopa and 400mg entacapone will be given one hour prior to the scan. Very few people experience any side-effects from these. Very occasionally people experience stomach upset, muscle movements, dry mouth and/or an orange tinge to their urine from the tablets, which may last a few hours to a day. This permits the use of a lower dose of 18F-DOPA than would otherwise be necessary. Participants will be asked to refrain from eating, drinking (apart from water) and smoking from midnight on the night before the scan, until after the scan is finished. This is because large amino acids may affect brain uptake of 18F-DOPA. Participants will also be instructed not to take illicit drugs (such as cannabis or cocaine) in the prior three days. Before the scan we will ask for a urine sample to check whether substances that can affect the scan are in their system. Women of childbearing age will have a pregnancy test and will be required to use regular contraception prior to the scan. At the start of the scan we will give participants a radiotracer (which is mildly radioactive) to measure the brain dopamine system. At the end of the scan the cannula will be removed from their arm.

Participants taking part in a PET scan at Imanova (SLaM/KCL participants) will have an additional 1-2 tablespoons (up to 30ml) of blood taken through their cannula to measure natural blood chemicals (hormones and genes) that are connected to dopamine function.

Participants taking part in a PET scan in Manchester will have all their bloods taken at this point (up to 50ml) as described under ‘Biological Samples Section’, whenever possible.

3. Sample Size, Statistics, Selection and Withdrawal of Subjects

The patients will be identified by members of the clinical team. Only the clinical team (who may also be part of the research team with NHS honorary contracts) will be able to access participant records and data prior to consent. No patient records will be screened by study researchers prior to consent. Study researchers will have access to patient records after/ if participants have consented to this.

We will recruit a total of 100 participants. Potential participants may be referred via clinical teams or other research studies/existing databases with consent to re-contact or registries and recruitment initiatives in NHS Trusts whose terms are in accordance with NHS Trust policies.

STRATA: Investigating factors associated with response to antipsychotic treatment
Protocol. Version 4.0, 19th August 2016
REC REF: 15/LO/0038

Participants will be first approached by a member of the clinical team or a member of the research team, with approval from the clinical team/ other study

Inclusion Criteria

1) aged 18-65;
2) DSM 5 schizophrenia/schizophreniform disorder.
3) Participants must read and write in English at a level sufficient to understand and complete study-related procedures

Exclusion Criteria

1) Pregnancy;
2) Severe head injury involving loss of consciousness >5 minutes (ever);
3) Meeting ICD criteria for harmful substance misuse or psychotic disorder secondary to substance misuse;
4) Participation in MRI scans requires exclusion of contraindications to MRI at 3 tesla e.g. metallic or electronic implants;
5) Severe claustrophobia.
6) Treatment with clozapine in the last 3 months

To establish and confirm the stratifier 1H-MRS data will be acquired in a total of 100 patients early in the course of their treatment; 50 T-Resp and 50 T-NonResp; matched for chronicity of illness.

Operational definition of T-Resp:
(i) treatment with only one antipsychotic drug since onset, or treatment changes have been due to adverse effects, not for non-response. (ii) CGI-SCH severity score of <4; (iii) PANSS total <60 (Leucht 2005); (iv) CRS >3

Operational definition of T-NonResp:
(i) documented treatment with at least two antipsychotics each above the minimum therapeutic dose as defined by the BNF for >4 weeks each; (ii) despite ongoing treatment and adequate adherence (assessed by iv) a CGI-SCH severity score of >3; (iii). PANSS total severity rating of at least 70 iv) Clinician Rating Scale (CRS; a measure of adherence) (Kemp et al 1996) >3.

Power and sample size calculation:
The study is powered to give >95% power to detect differences found in Egerton et al 2012 (α=0.05, allowing for 10% loss of sensitivity due to combining data from multiple centres). We have more than 80% power to detect a significant difference between a ROC curve with AUC 0.7 and chance, assuming α=0.05, 2-tailed. Two-tailed 18F-DOPA PET data will be acquired in a subset at 2 sites (N=60) to determine if the double dissociation between DA function and GLU function we have seen in chronic patients is also evident early in the illness course, where the strategy is most likely to be used (T-Resp n=30, T-NonResp n=30; powered to give >95% power to detect differences found in Demjaha et al 2012; α=0.05, allowing for 10% loss of sensitivity due to combining data from multiple centres).

Summary statistics will be used to describe the demographic and clinical characteristics of each participant group.

Group differences in demographic, clinical variables and 18F-DOPA Ki and glutamate concentration will be determined using pre-specified between group comparisons as appropriate (e.g. Chi square; Fischer's Exact; ANOVA).

Missing data will be minimal given that data is being collected prospectively. The exact reason for the missing data will be recorded. Any blank measures or spurious data will be checked against the paper copy of the CRF stored securely at sites.

Participants will be clearly told they can withdraw from the study at any time without having to give a reason. This is clear in the information sheet and the researcher will also explain this verbally to participants during the informed consent process**.** If a participant wishes to withdraw from a study all their identifiable data will be destroyed. Data or tissue already collected with the consent, which is not identifiable, would be retained and used in the study. No further data or tissue would be collected or any other research procedures carried out on or in relation to the participant.

Control group
We will recruit up to 15 healthy volunteers aged 18-65 to be scanned at each PET site (two sites; Imanova Limited, Imperial College London and The Wolfson Molecular Imaging Centre in Manchester) and 10 healthy volunteers aged 18-65 to be scanned at each MRI site (4 sites). This is to determine inter-site scanner variability and to provide normal range data for comparison with the clinical groups. In addition to the exclusion criteria above, healthy volunteers will be excluded if there is a history of schizophrenia or other psychotic disorder. Healthy volunteers will be recruited using an existing database of interested potential participants held at KCL.

4. Study procedures

Informed Consent

1) Participants will be first approached by a member of the clinical team or a member of the research team, with approval from the clinical team.
2) The study will be described verbally to potential participants and they will be given a copy of the information sheet. They will be encouraged to ask questions about the research. Potential participants will be allowed as much time as they require to make a decision and at least 24 hours so they are able to seek advice from others about participation, including previous participants in the research where possible.
3) If a patient expresses an interest in taking part, capacity to consent will be assessed and documented by the research team, in consultation with the clinical team.
4) If the patient has capacity to consent and agrees to participate in the study, they will be asked to sign and date two copies of the consent form. One copy will be kept by the participant and one by the research team. The research team will pass onto the clinical team to scan into medical notes, or incorporate in paper notes.
5) The participant will be informed that they can withdraw consent at any time, and without giving a reason.
6) Participants will be informed they are to be compensated for their time and travel expenses. This monetary amount will be up to £120 (£145 in Manchester) depending on which parts of the study the participant is involved with.

Within Avon and Wiltshire Mental Health Partnership NHS Trust, Everyone Included will be used to identify potentially eligible participants. Potentially eligible participants are identified based on the study inclusion /exclusion criteria, excluding those who have declined to receive information. This is done via an automated search of the Trust's electronic patient record system (RiO). An authorised search will be requested by a member or the R&D department, who are part of the clinical team and carried out by a member of the Information Analysis team. A data set is returned directly to the Everyone Included Administrators for processing the letters. No patient identifiable data will ever leave the Trust or be accessed by an external research team during this process.

The 'Research Opportunity Letter' will be sent to these individuals. The letter itself will not contain any patient identifiable or disclosing information (such as making reference to their diagnosis or medications). It will provide a free-post return slip and contact details (phone, email, website, postal address) inviting individuals to get in touch if they would like to further information / to take part. The onus is on the individual to express an interest, otherwise no further action is taken.

Upon responding to the 'Research Opportunity Letter', a Participant Information Sheet will be provided. If the research team is external, the individual will be asked if they are happy for their details (i.e. name and phone number) to be passed directly to the research team. No information is ever accessed by or passed to an external research team without first gaining permission from the potential participant. At this point standard study recruitment processes proceed.

Risks and burdens

The questionnaires involve personal questions and recalling experiences that some people may find distressing. Participants will be told if they feel uncomfortable with any of the questions they do not have to answer them.

Blood sampling and placing the cannula can cause some discomfort, and there is a possibility that a small bruise may develop. This task will be performed by research workers trained in phlebotomy. Any risks of infection will be contained by using standard sterile procedures and the risks associated with this task will be the same as for any other blood sample collection.

Any participants who become distressed during any procedure involved in this study will be encouraged to pause and will be reminded routinely that they can withdraw from the study at any time without a reason or penalty.

Any clinically significant issues that may arise during the assessment, the verbal consent will be obtained from the patient to pass onto the responsible psychiatrist or other relevant member of the staff. This will always be done with the participants' permission and will only be breached in the rare cases when there is judged to be an issue of safety, for example if the participant makes specific threats towards an individual.

Imaging

The MRI and PET scans themselves are painless and safe. Some people find the scans claustrophobic or anxiety-provoking. There is a mock scanner that participants can try out first if they wish. Participants will be told if they feel uncomfortable the scanning can be stopped at any time. Before the scan we will go through a safety questionnaire, to check that participants can have the scan. If they have any metal in their body, either from accidents or operations, they may

not be able to have the MRI scan, but they can still take part in the rest of the study. Clinical Research workers and research workers will log screening results.

Very occasionally people experience side effects from the medication they receive when taking part in a PET scan. These side-effects can include stomach upsets, muscle movements, dry mouth and/or an orange tinge to their urine. These side-effects may last a few hours to a day but participants will be warned about this and told it is nothing to worry about.

PET scans involve a small amount of radiation. Any exposure to radiation carries a risk of damaging the body's tissues and possibly triggering cancer at a later date. However, the risk is very small. A standard PET scan in this study will expose participants to 3.7mSv, (this may be 3.72mSv in Manchester if participants decide to have the extra, high resolution PET scan), which is the same amount of radiation that they are exposed to from natural sources of radiation, such as the sun, over the course of 18 months. In extremely rare cases the PET scan may need to be repeated and we have ARSAC approval for a maximum of 7.5mSv exposure per participant. Most experts believe that the risk of cancer developing only becomes significant in people who are exposed to 100mSv or more. However, as a precaution we are excluding pregnant or breastfeeding women. A pregnancy test will be carried out on female urine samples before the PET scan is conducted. Participants will be asked to consent to this on the consent form. Clinical Research workers and research workers will log screening results and ensure participants will not be exposed to more than 10mSv in 12 months (ARSAC guidelines suggest 10mSv as the normal upper limit for radiation exposure related to research procedure)

5. Sample handling and laboratories

Biological sample collection tubes and barcodes will be sent to sites in advance from the MRC Center for Neuropsychiatric Genetics and Genomics, Cardiff University. Samples will be stored in laboratories at sites and transportation will be organized when required (likely at 6 monthly basis, dependent on recruitment). Details of sample collection and storage at site will be recorded. Study SOPs will describe collection and storage specifications to ensure all sites are following the same guidelines.

When samples arrive at Cardiff University, researchers will ensure that the physical integrity of these samples have not been compromised in transit and track the samples in using their barcodes. The research team at Cardiff will notify the sponsor and the other study teams of any issues in transportation.

Cardiff University will extract DNA from the blood. We will perform genome-wide and targeted genotyping and/or exome or whole genome sequencing. We will seek genetic association with the imaging and other outcome measures at the level of individual genotype/sequence variant, genes, gene sets/pathways and polygenic or other summary scores.

A urine and blood sample will undergo metabolomic analysis at MRC National Phenome Centre. The Centre is funded by the MRC and NIHR and led by Imperial College London and King's College London. An additional blood sample will undergo proteomic analysis at the University of Manchester.

KCL/South London and the Maudsley (SLaM) participants will also be invited to participate in the BRC Biobank. This is covered by a separate ethical approval (09/H0606/84 NRES Committee South Central-Oxford C.)

6. Assessment of Safety

There are no serious adverse events expected to occur during the study.

All blood samples will only be taken by researchers trained in phlebotomy. All risks are the same as for any routine blood sample and are therefore minimal.

The drugs administered and the radiotracer used for the PET are standard procedures. The drugs administered may cause stomach upsets, muscle movements, dry mouth and/or an orange tinge to their urine. These side-effects may last a few hours to a day but participants will be warned about this and told it is nothing to worry about. Female participants will have a pregnancy test in advance.

For MRI scans a safety questionnaire will be carried out prior to the scan to check the participant does not have any metal in their bodies from operations or accidents.

7. Study oversight arrangements

STRATA is a multi-centred study and this will be managed by attendance at a monthly Consortium Executive meeting which will be responsible for the effective oversight of the daily activities of the study. Quarterly Consortium Board (CB) teleconferences will oversee the progress of, and interaction between, the workstreams to maintain communication of issues and progress between sites across the different aspects of STRATA. The CB will submit six-monthly Programme reports to the funder, MRC.

The project team consists of a full time Project Manager based at the IoPPN, KCL and a 50% Project Manager at the University of Manchester.

8. Ethics & Regulatory Approvals

REC name and address: South East Coast-Surrey Research Ethics Committee, Whitefriars, Level 3, Block B, Lewins Mead, Bristol, BS1 2NT

This study has also been reviewed and approved by the Administration of Radioactive Substances Advisory Committee (ARSAC).

9. Data Handling

Once participants have consented to be in the study some personal details will be taken. These details will be taken by the researcher with full consent to do so. These details will be kept securely at sites and used to contact patients when required to make appointments. No personal data will be shared with anyone outside of that study team. Each participant will be given a unique identifier and any clinical or genetic or imagining data relating to the same participant will link via that code.

Data will be entered and stored on a secure web application called Research Electronic Data Capture (REDCap). REDCap will not store any personal details and all participants will have a unique non-identifiable ID code. This unique ID code will then be used to merge all processed imaging, genetics and clinical data. REDCap will be hosted on secure servers at the Biomedical

Research Centre at Kings College London. All sites can access REDCap for the purposes of data entry via a web browser and data is uploaded when a WIFI signal is available.

The CI will ensure that the trial is conducted in compliance with the principles of the Declaration of Helsinki (1996), and in accordance with all applicable regulatory requirements including but not limited to the Research Governance Framework, Trust and Research Office policies and procedures and any subsequent amendments.

10. Finance and Publication Policy

STRATA is funded by a £ £4,900,000 Medical Research Council grant. Kings College London will receive and manage this funding. A collaboration agreement has agreed budgets between sites.

Analysis and findings from the study will be published as papers in journals. No identifiable data will be included.

This study has been adopted onto the UKCRN Portfolio and the research project will be registered on their database which is publicly available.
