# Supplementary material for: Cross-sectional study comparing cognitive function in treatment responsive versus treatment non-responsive schizophrenia: evidence from the STRATA study
Source: BMJ Open. 2021 Nov 25;11(11):e054160. doi: 10.1136/bmjopen-2021-054160 (PMC8627394; doi:10.1136/bmjopen-2021-054160)
Supplement: Supplementary data [file bmjopen-2021-054160supp002.pdf]

Supplementary material
Table S.1
Univariable and multivariable linear regression models for response status and BACS performance

	R	NR	Unadjusted				Adjusted for age, gender, illness duration and anticholinergic effects				Adjusted for anticholinergic effects			
BACS measure	N	N	β	SE	95%CI	P-value	β	SE	95%CI	P-value	β	SE	95%CI	P-value
Verbal Memory	53	50	-1.99	2.34	-6.63 ; 2.66	.398	-3.18	2.38	-7.90 ; 1.54	.185	-2.34	2.35	-7.00 ; 2.32	.322
Digit Sequencing	53	50	0.11	0.90	-1.67 ; 1.89	.901	0.07	0.92	-1.76 ; 1.89	.944	-0.02	0.90	-1.81 ; 1.77	.983
Verbal Fluency	53	50	1.23	1.86	-2.46 ; 4.91	.510	1.08	1.94	-2.78 ; 4.94	.580	1.17	1.88	-2.56 ; 4.90	.536
Token Motor	53	49	-0.42	2.95	-6.28 ; 5.43	.886	-1.40	2.97	-7.29 ; 4.50	.638	-0.62	2.99	-6.56 ; 5.31	.835
Symbol Coding	53	50	-1.84	2.28	-6.37 ; 2.68	.421	-1.89	2.37	-6.60 ; 2.83	.428	-2.04	2.30	-6.60 ; 2.53	.378
Tower of London	53	50	0.40	0.82	-1.23 ; 2.03	.625	0.35	0.84	-1.30 ; 2.01	.672	0.23	0.82	-1.40 ; 1.85	.782
z score composite	53	49	-0.03	0.29	-0.60 ; 0.54	.922	-0.08	0.30	-0.68 ; 0.52	.798	-0.07	0.29	-0.65 ; 0.50	.800
t score composite	53	49	-0.64	2.87	-6.32 ; 5.05	.825	-1.33	3.02	-7.32 ; 4.67	.662	-1.24	2.88	-6.96 ; 4.48	.668

Note. R = antipsychotic responder; NR = antipsychotic non-responder; BACS = Brief Assessment of Cognition in Schizophrenia; CIs = confidence intervals.
