# Supplementary material for: Cross-sectional study comparing cognitive function in treatment responsive versus treatment non-responsive schizophrenia: evidence from the STRATA study
Source: BMJ Open. 2021 Nov 25;11(11):e054160. doi: 10.1136/bmjopen-2021-054160 (PMC8627394; doi:10.1136/bmjopen-2021-054160)
Supplement: Author's manuscript [file bmjopen-2021-054160.draft_revisions.pdf]

BMJ Open is committed to open peer review. As part of this commitment we make the peer review history of every article we publish publicly available.

When an article is published we post the peer reviewers' comments and the authors' responses online. We also post the versions of the paper that were used during peer review. These are the versions that the peer review comments apply to.

The versions of the paper that follow are the versions that were submitted during the peer review process. They are not the versions of record or the final published versions. They should not be cited or distributed as the published version of this manuscript.

BMJ Open is an open access journal and the full, final, typeset and author-corrected version of record of the manuscript is available on our site with no access controls, subscription charges or pay-per-view fees (http://bmjopen.bmj.com).

If you have any questions on BMJ Open's open peer review process please email info.bmjopen@bmj.com

BMJ Open

Cognitive function and antipsychotic response in schizophrenia: evidence from the STRATA study.

Journal:	*BMJ Open*
Manuscript ID	bmjopen-2021-054160
Article Type:	Original research
Date Submitted by the Author:	03-Jun-2021
Complete List of Authors:	Millgate, Edward; King's College London Institute of Psychiatry Psychology and Neuroscience, Department of Psychosis Studies<br>Kravariti, Eugenia; King's College London Institute of Psychiatry Psychology and Neuroscience, Department of Psychosis Studies; NIHR Biomedical Research Centre at Guy's and Saint Thomas' NHS Foundation Trust and King's College<br>Egerton, Alice; King's College London Institute of Psychiatry Psychology and Neuroscience, Department of Psychosis Studies; NIHR Biomedical Research Centre at Guy's and Saint Thomas' NHS Foundation Trust and King's College<br>Howes, Oliver; King's College London Institute of Psychiatry Psychology and Neuroscience, Department of Psychosis Studies; NIHR Biomedical Research Centre at Guy's and Saint Thomas' NHS Foundation Trust and King's College<br>Murray, Robin; King's College London Institute of Psychiatry Psychology and Neuroscience, Department of Psychosis Studies; NIHR Biomedical Research Centre at Guy's and Saint Thomas' NHS Foundation Trust and King's College<br>Kassoumeri, Laura; King's College London Institute of Psychiatry Psychology and Neuroscience, Department of Psychosis Studies; NIHR Biomedical Research Centre at Guy's and Saint Thomas' NHS Foundation Trust and King's College<br>Donocik, Jacek; King's College London Institute of Psychiatry Psychology and Neuroscience, Department of Psychosis Studies<br>Lewis, Shon; The University of Manchester Division of Psychology and Mental Health; Greater Manchester Mental Health NHS Foundation Trust<br>Drake, Richard; The University of Manchester Division of Psychology and Mental Health; Greater Manchester Mental Health NHS Foundation Trust<br>Lawrie, Stephen; The University of Edinburgh Division of Psychiatry, Psychiatry<br>Murphy , Anna; The University of Manchester Division of Psychology and Mental Health; Greater Manchester Mental Health NHS Foundation Trust<br>Collier, Tracy; King's College London Institute of Psychiatry Psychology and Neuroscience, Department of Psychosis Studies<br>Lees, Jane; The University of Manchester Division of Psychology and Mental Health; Greater Manchester Mental Health NHS Foundation Trust<br>Stockton-Powdrell, Charlotte; The University of Manchester Division of Psychology and Mental Health<br>Walters, James; Cardiff University<br>Deakin, JFW; The University of Manchester Division of Psychology and Mental Health

	MacCabe, James; King's College London Institute of Psychiatry Psychology and Neuroscience, Department of Psychosis Studies; NIHR Biomedical Research Centre at Guy's and Saint Thomas' NHS Foundation Trust and King's College
Keywords:	Schizophrenia & psychotic disorders < PSYCHIATRY, PSYCHIATRY, Adult psychiatry < PSYCHIATRY

SCHOLARONE™
Manuscripts

I, the Submitting Author has the right to grant and does grant on behalf of all authors of the Work (as defined in the below author licence), an exclusive licence and/or a non-exclusive licence for contributions from authors who are: i) UK Crown employees; ii) where BMJ has agreed a CC-BY licence shall apply, and/or iii) in accordance with the terms applicable for US Federal Government officers or employees acting as part of their official duties; on a worldwide, perpetual, irrevocable, royalty-free basis to BMJ Publishing Group Ltd ("BMJ") its licensees and where the relevant Journal is co-owned by BMJ to the co-owners of the Journal, to publish the Work in this journal and any other BMJ products and to exploit all rights, as set out in our licence.

The Submitting Author accepts and understands that any supply made under these terms is made by BMJ to the Submitting Author unless you are acting as an employee on behalf of your employer or a postgraduate student of an affiliated institution which is paying any applicable article publishing charge ("APC") for Open Access articles. Where the Submitting Author wishes to make the Work available on an Open Access basis (and intends to pay the relevant APC), the terms of reuse of such Open Access shall be governed by a Creative Commons licence – details of these licences and which Creative Commons licence will apply to this Work are set out in our licence referred to above.

Other than as permitted in any relevant BMJ Author's Self Archiving Policies, I confirm this Work has not been accepted for publication elsewhere, is not being considered for publication elsewhere and does not duplicate material already published. I confirm all authors consent to publication of this Work and authorise the granting of this licence.

For peer review only

Journal: BMJ Open

Word count: 2531 *(Table word count = 423)*

Abstract word count: 252

Submission date: 3rd June 2021

For peer review only

Cognitive function and antipsychotic response in schizophrenia: evidence from the STRATA study.

Edward Millgate[1], Eugenia Kravariti[1,2], Alice Egerton[1,2], Oliver D Howes[1,2], Robin M Murray[1,2], Laura Kassoumeri[1,2], Jacek Donocik[1], Shôn Lewis[3,4], Richard Drake[3,4], Stephen M Lawrie[5], Anna Murphy[3,4], Tracy Collier[1], Jane Lees[3,4], Charlotte Stockton-Powdrell[3], James Walters[6], Bill Deakin[3,4] and James H MacCabe[1,2]*

[1] Institute of Psychiatry, Psychology and Neuroscience, King's College London, United Kingdom

[2] NIHR Biomedical Research Centre at South London and Maudsley NHS Foundation Trust, London, United Kingdom

[3] Division of Psychology and Mental Health, School of Biological Sciences, Faculty of Biology, Medicine and Health, University of Manchester, Manchester

[4]Greater Manchester Mental Health NHS Foundation Trust, Manchester, United Kingdom

[5]Division of Psychiatry, University of Edinburgh, Edinburgh, United Kingdom

[6]Division of Psychological Medicine and Clinical Neuroscience, Cardiff University, Cardiff, United Kingdom

*Corresponding author: james.maccabe@kcl.ac.uk

Department of Psychosis Studies,

Institute of Psychiatry, Psychology & Neuroscience,

16 de Crespigny Park,

Denmark Hill, London,

United Kingdom, SE5 8AF

For peer review only - http://bmjopen.bmj.com/site/about/guidelines.xhtml

Abstract

Background: Failure to respond to antipsychotic treatment affects up to a third of patients with a schizophrenia diagnosis and are later termed treatment resistant. (TRS). 70-84% of individuals with TRS show antipsychotic non-response (NR) from the first episode. Emerging cross-sectional evidence comparing cognitive profiles in treatment resistant schizophrenia (TRS) to treatment-responsive schizophrenia has indicated that verbal memory and language functions may be more impaired in TRS. We sought to confirm this finding by comparing cognitive performance between antipsychotic non-responders (NR) and responders (R).

Design: Cross-sectional

Setting: This cross-sectional study recruited antipsychotic treatment responders (R) and antipsychotic non-responders (NR) across four UK sites. Cognitive performance was assessed using the Brief Assessment of Cognition in Schizophrenia (BACS).

Participants: 106 participants aged 18 – 65 years with a diagnosis of schizophrenia or schizophreniform disorder were recruited according to their treatment response, with 52 NR, and 54 R cases.

Outcomes: Composite and subscale scores of cognitive performance on the BACS. Group (R vs NR) differences in cognitive scores were investigated using univariable and multivariate linear regressions adjusted for age and gender.

Results: There were no significant differences in cognitive performance on BACS composite or subscale scores between R and NR groups.

Conclusions: The lack of group difference in cognition in our sample is likely due to a lack of clinical distinction between our groups. Future investigations should aim to investigate the role of cognitive function in antipsychotic response in early in the illness stage and how this may differ from antipsychotic responders.

Trail registration number: REC: 15/LO/0038.

Keywords: cognition, treatment resistance, antipsychotic response, schizophrenia, BACS

For peer review only

For peer review only - http://bmjopen.bmj.com/site/about/guidelines.xhtml

Article summary

Strengths and limitations of this study

- The study examined cognitive performance in a relatively large and multicentre sample of antipsychotic responders and non-responders
- Cognition was assessed on the BACS, a reliable and brief test battery specifically designed for schizophrenia
- The lack of significant group differences in cognition between antipsychotic responders and non-responders may reflect limited clinical separation between these groups.

For peer review only

For peer review only - http://bmjopen.bmj.com/site/about/guidelines.xhtml

Introduction

Up to a third of patients with a schizophrenia diagnosis have inadequate symptomatic improvement despite having at least two antipsychotic drugs, one being a second-generation antipsychotic excluding clozapine, at adequate doses and duration (4 – 6 weeks; NICE guidelines) [1] and are termed treatment resistant (TRS) [2,3]. Almost all guidelines recommend the antipsychotic clozapine in TRS[4], with earlier clozapine treatment associated with better outcomes [5-8]. There is increasing evidence that TRS may represent a distinct subtype in schizophrenia [9,10]. Most treatment resistant cases exhibit antipsychotic non-response (NR) from the first episode, with this observed in 70-84% of patients [3,11]. An earlier age of onset has also been consistently associated with antipsychotic treatment resistance [12-16], suggesting that TRS and NR may be associated with neurodevelopmental impairment. Identifying these underlying factors associated with antipsychotic treatment resistance in schizophrenia is therefore important for improving prediction and early treatment of NR and TRS.

Cognitive impairment in schizophrenia may provide some insight into antipsychotic treatment response. Performance on tasks of verbal memory has often been reported to be impaired in schizophrenia samples [17], including those prior to medication initiation [18], and at first episode [19,20]. In a recent meta-analysis, comparing mostly cross-sectional studies of treatment resistant cases and responders, TRS cases exhibited greatest cognitive impairments on tasks of verbal memory and learning (dl = -0.59, $p < .001$) and language functions (dl = -0.53, $p < .001$), with smaller but still statistically significant impairments in tasks across other cognitive domains, relative to their responder counterparts [21].

However, this meta-analysis[21] included an array of cognitive tasks, many with long test duration and stringent training requirements for raters. Short and comprehensive measures of cognitive performance may aid in the detection of neuropsychological differences between antipsychotic responders (R) and non-responders (NR), while also being cost-effective. The Brief Assessment of Cognition in Schizophrenia (BACS) [22] was originally developed to be an easily administrable, brief, test battery that efficiently and specifically assesses cognitive deficits in schizophrenia cases. The measures included in the battery correspond to several cognitive domains with established deficits in schizophrenia; executive functions [23,24], working memory [25,26], motor/processing speed [27], verbal memory [28,29], verbal fluency [30,31] and attention [32,33]. If observable differences between antipsychotic responders

For peer review only - http://bmjopen.bmj.com/site/about/guidelines.xhtml

and non-responders are identified, then, as well as improving our understanding of cognitive factors implicated in the aetiology of antipsychotic response, this would raise the possibility for future prospective research to use brief cognitive testing as part of predictive/diagnostic models for antipsychotic response and future treatment resistance.

Therefore, this cross-sectional study sought to assess the cognitive profiles of antipsychotic responders and non-responders utilising the Brief Assessment of Cognition in Schizophrenia. Based on the existing literature, we hypothesised that TRS patients would have poorer performance across BACS tasks, particularly on verbal memory and verbal fluency tasks.

Methods

Design

The study used a cross-sectional design comparing antipsychotic treatment responders (R) and antipsychotic non-responders (NR) on cognitive performance.

Setting

The study was part of 'Schizophrenia: Treatment Resistance and Therapeutic Advances' (STRATA), a consortium which included King's College London (London, UK), University of Manchester (Manchester, UK), Cardiff University (Wales, UK) and University of Edinburgh (Scotland, UK). The aim of the STRATA consortium is to identify neurobiological, cognitive and genetic biomarkers of antipsychotic treatment resistance and non-response within schizophrenia and other related psychotic disorders.

Participants

106 participants were recruited following a screening of patients across four sites: King's College London (N = 38), University of Manchester (N = 32), Cardiff University (N = 16) and University of Edinburgh (N = 18). Inclusion criteria were as follows: aged 18 – 65 years, with a schizophrenia or schizophreniform disorder diagnosis as per Diagnostic and Statistical Manual of Mental Disorders, 5th edition (DSM-5) [34] criteria and be able to read and write English to a sufficient level (see also Egerton et al [35]). Participants were excluded if they were pregnant, had ever experienced a head injury involving loss of consciousness for

For peer review only - http://bmjopen.bmj.com/site/about/guidelines.xhtml

more than 5 minutes, met ICD criteria for harmful substance misuse or a psychotic disorder secondary to substance use, scored < 3 on the Clinical Rating Scale (a measure of adherence) [36,37], or had been treated with clozapine in the previous three months. All participants gave informed consent prior to enrolment. This study was approved by the South East Coast-Surrey Research Ethics Committee; REC: 15/LO/0038.

Definition of antipsychotic response and antipsychotic non-response

Participants were defined as antipsychotic treatment responders (R) if they had been treated with only one antipsychotic drug since illness onset, or if their antipsychotic drug had been changed only for reasons of adverse effects as opposed to non-response. In addition to this, responders needed to have a Clinical Global Impression score (CGI-SCH) [38] of below 4 (*moderately ill*), a Positive and Negative Syndrome scale (PANSS) [39] total score below 60, and a Clinical Rating Scale (CRS) [36,37] level of adherence greater than 3 (‘*accepts only because compulsory*’). 54 treatment responders were recruited into the study.

Antipsychotic treatment non-response (NR) was defined as having documented treatment with at least two antipsychotics each above the minimum therapeutic dose as defined by the British National Formulary for > 4 weeks each, a CGI-SCH severity score of > 3, a PANSS total severity rating of at least 70, and a CRS adherence score of > 3. 52 participants met criteria for antipsychotic non-response.

Materials

Clinical and demographic measures

Previous and existing drug use were measured using the Alcohol, Drug and Tobacco Inventory. Participants’ disorder severity was measured using the Mini-International Neuropsychiatric Interview (M-Psychotic Disorders; A-Major Depressive Episode; D-Manic/Hypomanic/Bipolar; MINI) [40], Structured Clinical Interview- Positive and Negative Syndrome Scale (SCI-PANSS) [41] and Clinical Global Impression-Schizophrenia scale (CGI-SCH) [38]. Concordance with medication was assessed using the Clinical Rating Scale for Schizophrenia (CRS) [36,37]. Participants also provided demographic data, such as years of previous full-time education, age, gender, as well as information regarding their previous antipsychotic history, which were supplemented by medical records.

For peer review only - http://bmjopen.bmj.com/site/about/guidelines.xhtml

Measures of cognitive performance

Cognitive data was collected using the Brief Assessment of Cognition in Schizophrenia (BACS) [22] across all sites at the beginning of the assessment, following the administration of clinical and demographic measures. The battery is designed to take ~30 mins to complete, with minimal training demands, and is designed to be easily administered by clinical and healthcare workers [22]. The BACS (version A) [22] consists of six tests from the following cognitive domains: i) Verbal Memory: List learning task; ii) Working Memory: Digit Sequencing task; iii) Motor Speed: Token motor task; iv) Verbal Fluency: Category instances task (Animals) and phonological (F and S-words); v) Attention and speed of information processing: Symbol Coding task; vi) Executive Functions: Tower of London task. All tasks on the BACS are scored with higher scores representing better performance. Composite z and t scores for the BACS are generated using normative data [42] and the following formulas: $Composite\ z\ score = \frac{\Sigma\left(\Sigma\frac{(raw\ score - normative\ score)}{normative\ standard\ deviation}\right)}{3.63}$ with each measure's z score summed and the total divided by 3.63; $Composite\ t\ score = (Composite\ z\ score * 10) + 50$.

Data analysis

All analyses were conducted using STATA 15/SE [43]. Chi-square tests were used to compare cognitive performance across sites in case of site differences. Univariable regressions were used to compare cognitive performance between groups. Multivariable regression analyses were used to adjust univariable results for age and gender, due to the reported relationship of age [44,45] and gender [46,47] with cognitive outcomes.

Results

Descriptive statistics of demographic and clinical variables between responder groups are reported in Table 1.

For peer review only - http://bmjopen.bmj.com/site/about/guidelines.xhtml

Table 1

Demographic and clinical characteristics by group

	R			NR		
Demographic/clinical variable	N	Mean/ratio	SD	N	Mean/ratio	SD
Age	54	29.52	9.36	52	29.99	8.50
Gender (male : female)	54	46 : 8	-	52	43 : 9	-
Age of illness onset	53	26.10	6.53	50	25.31	5.93
Illness duration since 1st antipsychotic (years)	53	3.71	6.87	50	5.03	5.79
Duration from 1st psychotic symptom (years)	54	4.81	7.53	52	5.50	6.13
Duration from 1st contact with mental health services (years)	54	4.04	7.49	52	5.40	6.34
Full time education (years)	53	13.09	2.37	50	12.88	2.75
Chlorpromazine equivalents (mg/day)	53	270.76	313.89	52	417.32	742.34
PANSS positive score	54	12.24	3.40	42	22.65	3.54
PANSS negative score	54	13.82	3.38	52	20.96	4.56
PANSS total score	54	53.46	7.91	52	87.29	9.30
CGI positive symptoms score	53	3.26	.76	52	5.50	.10
CGI negative symptoms score	53	3.21	.86	52	4.88	1.04
CGI cognitive symptoms score	53	3.08	.83	52	4.83	1.22
CGI overall severity	53	3.42	.75	52	5.48	.58

Note. R = antipsychotic responder; NR = antipsychotic non-responder; PANSS = Positive and Negative Syndrome Scale; CGI = Clinical Global Impression.

Cognitive performance

Mean scores for each group on all BACS tasks and standardized composite scores are displayed in Table 2. All measures of the BACS were normally distributed, with exception of the Tower of London task which was moderately negatively skewed (skewness = -0.95) as per the guidelines from Bulmer [48]. Cognitive performance on BACS composite and subtests did not significantly differ by site where data was collected.

For peer review only - http://bmjopen.bmj.com/site/about/guidelines.xhtml

Table 2

Mean group performance on BACS measures and univariable and multivariable linear regression models for response status and BACS performance

	R			NR			Unadjusted				Adjusted for age and gender			
BACS measure	N	Mean	SD	N	Mean	SD	β	SE	95%CI	P-value	β	SE	95%CI	P-value
Verbal Memory	53	38.89	10.66	50	36.9	13.04	-1.99	2.34	-6.63 ; 2.66	.398	-2.06	2.33	-6.68 ; 2.55	.377
Digit Sequencing	53	17.87	4.95	50	17.98	4.09	0.11	0.90	-1.67 ; 1.89	.901	0.12	0.89	-1.64 ; 1.88	.893
Verbal Fluency	53	30.45	9.04	50	31.68	9.82	1.23	1.86	-2.46 ; 4.91	.510	1.20	1.87	-2.51 ; 4.91	.522
Token Motor	53	66.32	14.56	49	65.90	15.26	-0.42	2.95	-6.28 ; 5.43	.886	-0.63	2.84	-6.27 ; 5.00	.824
Symbol Coding	53	47.30	11.31	50	45.46	11.83	-1.84	2.28	-6.37 ; 2.68	.421	-1.87	2.28	-6.39 ; 2.65	.413
Tower of London	53	16.04	4.46	50	16.44	3.83	0.40	0.82	-1.23 ; 2.03	.625	0.42	0.82	-1.21 ; 2.05	.609
z score composite	53	-2.00	1.39	49	-2.03	1.51	-0.03	0.29	-0.60 ; 0.54	.922	-0.26	0.29	-0.60 ; 0.55	.928
t score composite	53	29.91	13.81	49	29.27	14.99	-0.64	2.87	-6.32 ; 5.05	.825	-0.61	2.89	-6.36 ; 5.13	.832

Note. R = antipsychotic responder; NR = antipsychotic non-responder; BACS = Brief Assessment of Cognition in Schizophrenia; CIs = confidence intervals.

For peer review only - http://bmjopen.bmj.com/site/about/guidelines.xhtml
For peer review only

Univariable linear regression analyses (Table 2) observed no significant relationships between response status and BACS performance. Multivariable models adjusted for age and gender also observed no significant relationships between response status and cognitive outcomes (Table 2).

Discussion

The present investigation sought to compare specific cognitive deficits in antipsychotic responders (R) and antipsychotic non-responders (NR) using the Brief Assessment of Cognition in Schizophrenia (BACS) [22], anticipating the greatest deficits for NR in measures of verbal memory and verbal fluency when compared to R. Unlike previous cross-sectional studies [49-56], this investigation identified no significant differences in cognitive performance between groups.

Previous cross sectional research investigating differences in cognitive performance between antipsychotic treatment responders and treatment resistant cases have identified poorer performance in verbal, executive function and full-scale IQ cognitive measures [49,50,53-55], and also verbal memory [49,52,54,56,58] in treatment resistant patients. A recent study using a similar methodology and sample size to ours also failed to show significant differences between antipsychotic responders and TRS cases on individual tasks of the BACS [57] but did observe significant differences on standardized (z and t) composite scores suggesting overall impairment in the TRS group.

The lack of significant differences in cognitive performance observed between R and NR groups in our study may be partly explained by the criteria used to define these groups. Unlike earlier investigations, our study did not include clozapine-treated patients, and there may have been less clinical separation between the R and NR groups than in some previous studies (as discussed in Egerton et al [35]). Furthermore, in our cross-sectional study design it is not possible to determine the proportion of participants in the NR group who would meet criteria for TRS [59]. It is therefore possible the non-responder group was less severely unwell as in some previous studies, which may have reduced the ability to observe potential impairments in cognition due to clinical overlap. Previous investigations which observed group differences in cognitive performance between R and TRS included patients prescribed clozapine [50,51,53-55,57,58], and reported higher PANSS positive, negative and total scores[53,54,58],

For peer review only - http://bmjopen.bmj.com/site/about/guidelines.xhtml

suggesting the NR/TRS groups may have had greater illness severity compared to our sample. Likewise, demographic and clinical variables previously found to be associated with antipsychotic response, such as a younger age and age of illness onset in non-responders [12-16], did not significantly differ between treatment responders and non-responders in our sample, again suggesting group that compared to previous investigations, there wasn't enough clinical separation between our samples. In addition, the power calculations for sample size were generated on the basis of being able to provide > 95% power to detect differences in levels of anterior cingulate glutamate [35] (see Protocol provided in supplementary material) and it is possible that the sample was underpowered to detect neurocognitive differences using the BACS. In previous investigations comparing performance between R and TRS cases, but using multiple measures of cognitive performance, our sample size is larger than most previous studies [50-54, 56, 58]. However, in the one study which identified only global differences between R and TRS groups on BACS composite total scores [57], a larger sample size was used (N=130), suggesting larger samples are needed to detect group differences on the BACS.

Another consideration is that our study focused on younger patients early in their treatment trajectories to reduce the potential effects of chronicity and previous medication, with a mean length of treatment of 3 to 4 years. Most previous cross-sectional investigations include older samples with a longer duration of illness [50,51,53,54,58], although differences in measures of verbal intelligence and fluency have been quantifiable at the first episode in treatment resistant psychosis[24]. Trajectory modelling of cognitive performance in FEP has observed deficits in executive function performance, relative to controls, with these remaining stable over illness duration [61]. However, deficits in verbal knowledge and memory became more apparent and exaggerated relative to controls following the first episode [60]. Similar exaggerated declines following the first episode have also been observed in measures of verbal memory [61,62]. With our sample of patients being early in their treatment, cognitive deficits may have been less marked at this illness stage.

Despite not detecting significant differences between antipsychotic responder groups, it is worth mentioning the importance of conducting research using clinically transferable measures of cognitive impairment. In Alzheimer's disease and elderly populations, brief measures such as the Mini Mental State Examination (MMSE) [63] or Addenbrooke's Cognitive Examination (ACE) [64] are readily used by clinicians to assess cognitive functioning trajectories. Recent research using the ACE has also found deficits in

For peer review only - http://bmjopen.bmj.com/site/about/guidelines.xhtml

schizophrenia in comparison to controls using this measure [65]. However, these measures either focus on too few aspects of cognition or are not developed with the deficits in schizophrenia as a primary focus. In contrast the BACS assess many cognitive domains in less time and with less training in comparison to traditional comprehensive batteries for schizophrenia but may require larger sample sizes to detect meaningful differences between responders and non-responders. In consideration of the wealth of evidence illustrating the impact of cognitive dysfunction in schizophrenia, future research should focus on standardizing brief cognitive batteries for clinical utility.

Conclusions

Within this cross-sectional investigation we observed no differences in cognitive performance between antipsychotic responders and non-responders. This may be because there was less clinical separation between these groups in our sample in comparison to previous investigations. Future investigations should consider the role of cognitive functions in antipsychotic response prospectively using first episode cohorts and how this may differ in future stages of treatment resistance, as well as establish the use of brief cognitive batteries for schizophrenia by clinical professionals.

Funding

STRATA is funded by a grant from the Medical Research Council (MRC) to JHM, grant reference MR/L011794. E.M.'s PhD is funded by the MRC-doctoral training partnership studentship in Biomedical Sciences at King's College London. J.H.M., E.K., R.M.M., A.E., O.D.H. are part funded by the National Institute for Health Research (NIHR) Biomedical Research Centre at South London and Maudsley NHS Foundation Trust and King's College London. In the past three years, S.M.L. has received personal support from Sunovion. The views expressed are those of the authors and not necessarily those of the NHS, the MRC, the NIHR, Sunovion or the Department of Health.

Acknowledgments

For more information on STRATA please visit; https://gtr.ukri.org/project/7F7F0378-8FD7-4A33-91AD-277A49EF4908). STRATA was funded by a grant from the Medical Research Council (MRC), to Prof James MacCabe. Data were collected between March 2015 and February 2017. The remaining authors report no conflicts of interest.

For peer review only - http://bmjopen.bmj.com/site/about/guidelines.xhtml

Data Availability

At the time of submission, the data governance frameworks are being put in place to make a fully anonymized version of the data available to the wider research community via the TranSMART data sharing platform: https://transmartfoundation.org/. To apply for access to the data, please contact J.H.M. at james.maccabe@kcl.ac.uk.

Author contributions

J.H.M., R.M.M., O.D.H., A.E., E.K., R.D. and S.M.L contributed to the design and implementation of the study. E.M. completed analyses and wrote the manuscript with the assistance of J.H.M. and E.K. J.H.M., R.M.M., O.D.H., A.E., E.K., S.M.L., J.D., S.L., provided comments on the manuscript.

For peer review only

References

1 National Institute for Health and Care Excellence, National Collaborating Centre for Mental Health (UK. Psychosis and schizophrenia in adults: treatment and management).

2 Elkis H. Treatment-resistant schizophrenia. *Psychiatric Clinics of North America*. 2007 Sep1;30(3):511-33.

3 Demjaha A, Lappin JM, Stahl D, et al. Antipsychotic treatment resistance in first-episode psychosis: prevalence, subtypes and predictors. *Psychological medicine*. 2017 Aug;47(11):1981-9.

4 Kane JM, Agid O, Baldwin ML, et al. Clinical guidance on the identification and management of treatment-resistant schizophrenia. *The Journal of clinical psychiatry*. 2019 Mar5;80(2):0-.

5 Üçok A, Çıkrıkçılı U, Ergül C, et al. Correlates of clozapine use after a first episode of schizophrenia: results from a long-term prospective study. *CNS drugs*. 2016 Oct;30(10):997-1006.

6 Köhler-Forsberg O, Horsdal HT, Legge SE, et al. Predictors of nonhospitalization and functional response in clozapine treatment: a nationwide, population-based cohort study. *Journal of clinical psychopharmacology*. 2017 Apr1;37(2):148-54.

7 Yoshimura B, Yada Y, So R, et al. The critical treatment window of clozapine in treatment-resistant schizophrenia: secondary analysis of an observational study. *Psychiatry research*. 2017 Apr1;250:65-70.

8 Griffiths K, Millgate E, Egerton A, et al. Demographic and clinical variables associated with response to clozapine in schizophrenia: a systematic review and meta-analysis. *Psychological Medicine*. 2021 Feb1:1-1.

9 Lally J, Gaughran F, Timms P, et al. Treatment-resistant schizophrenia: current insights on the pharmacogenomics of antipsychotics. *Pharmacogenomics and personalized medicine*. 2016;9:117.

10 Gillespie AL, Samanaite R, Mill J, et al. Is treatment-resistant schizophrenia categorically distinct from treatment-responsive schizophrenia? A systematic review. *BMC psychiatry*. 2017 Dec;17(1):1-4.

11 Lally J, Ajnakina O, Di Forti M, et al. Two distinct patterns of treatment resistance: clinical predictors of treatment resistance in first-episode schizophrenia spectrum psychoses. *Psychological medicine*. 2016 Nov;46(15):3231-40.

For peer review only

12 Wimberley T, Støvring H, Sørensen HJ, et al. Predictors of treatment resistance in patients with schizophrenia: a population-based cohort study. *The Lancet Psychiatry*. 2016 Apr1;3(4):358-66.

13 Smart SE, Kępińska AP, Murray RM et al. Predictors of treatment resistant schizophrenia: a systematic review of prospective observational studies. *Psychological medicine*. 2021 Jan;51(1):44-53.

14 Legge S, Dennison C, Pardinas A, et al. Clinical indicators of treatment-resistant psychosis. *British Journal of Psychiatry*. 2020 May31;216(5):259-66.

15 Bozzatello P, Bellino S, Rocca P. Predictive factors of treatment resistance in first episode of psychosis: a systematic review. *Frontiers in psychiatry*. 2019 Feb26;10:67.

16 Smart S. *Clinical and Genetic Predictors of Treatment Resistant Psychosis* (Doctoral dissertation, King's College London).

17 Toulopoulouand T, Murray RM. Verbal memory deficit in patients with schizophrenia: an important future target for treatment. *Expert review of neurotherapeutics*. 2004 Jan1;4(1):43-52.

18 Fatouros-Bergman H, Cervenka S, Flyckt L, et al. Meta-analysis of cognitive performance in drug-naïve patients with schizophrenia. *Schizophrenia research*. 2014 Sep1;158(1-3):156-62.

19 Mesholam-Gately RI, Giuliano AJ, Goff KP, et al. Neurocognition in first-episode schizophrenia: a meta-analytic review. *Neuropsychology*. 2009 May;23(3):315.

20 Kravariti E, Morgan K, Fearon P, et al. Neuropsychological functioning in first-episode schizophrenia. *The British Journal of Psychiatry*. 2009 Oct;195(4):336-45.

21 Millgate E, Hide O, Lawrie S, et al. Neuropsychological differences between treatment-resistant and treatment-responsive schizophrenia: a systematic review & meta-analysis. [Manuscript submitted for publication]. Institute of Psychiatry, Psychology and Neuroscience, King’s College London

22 Keefe RS, Goldberg TE, Harvey PD, et al. The Brief Assessment of Cognition in Schizophrenia: reliability, sensitivity, and comparison with a standard neurocognitive battery. *Schizophrenia research*. 2004 Jun1;68(2-3):283-97.

23 Velligan DI, Bow-Thomas CC. Executive function in schizophrenia. InSeminars in clinical neuropsychiatry 1999 Jan1(Vol. 4, No. 1, pp. 24-33).

24 Kravariti E, Dixon T, Frith C, et al. Association of symptoms and executive function in schizophrenia and bipolar disorder. *Schizophrenia research*. 2005 May1;74(2-3):221-31.

For peer review only - http://bmjopen.bmj.com/site/about/guidelines.xhtml

25 Lee J, Park S. Working memory impairments in schizophrenia: a meta-analysis. *Journal of abnormal psychology*. 2005 Nov;114(4):599.

26 Forbes NF, Carrick LA, McIntosh AM, et al. Working memory in schizophrenia: a meta-analysis. *Psychological medicine*. 2009 Jun1;39(6):889.

27 Flashman LA, Flaum M, Gupta S, et al. Soft signs and neuropsychological performance in schizophrenia. *The American journal of psychiatry*. 1996 Apr.

28 Bruder GE, Wexler BE, Sage MM, et al. Verbal memory in schizophrenia: additional evidence of subtypes having different cognitive deficits. *Schizophrenia Research*. 2004 Jun1;68(2-3):137-47.

29 Hill SK, Beers SR, Kmiec JA, et al. Impairment of verbal memory and learning in antipsychotic-naıve patients with first-episode schizophrenia. *Schizophrenia research*. 2004 Jun1;68(2-3):127-36.

30 Bokat CE, Goldberg TE. Letter and category fluency in schizophrenic patients: a meta-analysis. *Schizophrenia research*. 2003 Nov1;64(1):73-8.

31 Ojeda N, Sánchez P, Peña J, et al. Verbal fluency in schizophrenia: does cognitive performance reflect the same underlying mechanisms in patients and healthy controls?. *The Journal of nervous and mental disease.* 2010 Apr1;198(4):286-91.

32 Harris JG, Minassian A, Perry W. Stability of attention deficits in schizophrenia. *Schizophrenia research*. 2007 Mar1;91(1-3):107-11.

33 Carter JD, Bizzell J, Kim C, et al. Attention deficits in schizophrenia—preliminary evidence of dissociable transient and sustained deficits. *Schizophrenia research*. 2010 Sep1;122(1-3):104-12.

34 American Psychiatric Association. Diagnostic and statistical manual of mental disorders (DSM-5®). American Psychiatric Pub; 2013 May 22.

35 Egerton A, Murphy A, Donocik J, et al. Dopamine and Glutamate in Antipsychotic-Responsive Compared With Antipsychotic-Nonresponsive Psychosis: A Multicenter Positron Emission Tomography and Magnetic Resonance Spectroscopy Study (STRATA). *Schizophrenia Bulletin*. 2020 Sep10.

36 Kemp R, Hayward P, Applewhaite G, et al. Compliance therapy in psychotic patients: randomised controlled trial. *Bmj.* 1996 Feb10;312(7027):345-9.

For peer review only - http://bmjopen.bmj.com/site/about/guidelines.xhtml

37 Kemp R, Kirov G, Everitt B, et al. Randomised controlled trial of compliance therapy. *The British Journal of Psychiatry*. 1998 May1;172(5):413-9.

38 Guy W. ECDEU assessment manual for psychopharmacology. US Department of Health, Education, and Welfare, Public Health Service, Alcohol, Drug Abuse, and Mental Health Administration, National Institute of Mental Health, Psychopharmacology Research Branch, Division of Extramural Research Programs; 1976.

39 Kay SR, Fiszbein A, Opler LA. The positive and negative syndrome scale (PANSS) for schizophrenia. *Schizophrenia bulletin.* 1987;13(2):261.

40 Sheehan DV, Lecrubier Y, Sheehan KH, et al. The Mini-International Neuropsychiatric Interview (MINI): the development and validation of a structured diagnostic psychiatric interview for DSM-IV and ICD-10. *Journal of clinical psychiatry*. 1998 Jan1;59(20):22-33.

41 Opler LA, Kay SR, Lindenmayer JP, et al. Structured clinical interview: The positive and negative syndrome scale (SCI-PANSS). North Tonawanda, NY: Multi-Health Systems. 1999.

42 Keefe RS, Harvey PD, Goldberg TE, et al. Norms and standardization of the Brief Assessment of Cognition in Schizophrenia (BACS). *Schizophrenia research.* 2008 Jul1;102(1-3):108-15.

43 StataCorp LL. Stata Statistical Software: Release 15 College Station, TX, 2017. www. stata. com/features/documentation/(last accessed on 1 March 2018). 2017.

44 Craik FI, Bialystok E. Cognition through the lifespan: mechanisms of change. *Trends in cognitive sciences*. 2006 Mar1;10(3):131-8.

45 Harvey PD. What is the evidence for changes in cognition and functioning over the lifespan in patients with schizophrenia?. *The Journal of clinical psychiatry.* 2014 Jun5;75(suppl 2):34-8.

46 Perlick D, Mattis S, Stastny P, et al. Gender differences in cognition in schizophrenia. *Schizophrenia Research.* 1992 Oct1;8(1):69-73.

47 Han M, Huang XF, Xiu MH, et al. Gender differences in cognitive function of patients with chronic schizophrenia. *Progress in neuro-psychopharmacology and biological psychiatry*. 2012 Dec3;39(2):358-63.

48 Bulmer MG. Principles of statistics. Courier Corporation; 1979.

49 Lawrie SM, Ingle GT, Santosh CG, et al. Magnetic resonance imaging and single photon emission tomography in treatment-responsive and treatment-resistant schizophrenia. *The British Journal of Psychiatry.* 1995 Aug1;167(2):202-10.

For peer review only - http://bmjopen.bmj.com/site/about/guidelines.xhtml

50 Smith RC, Kadewari RP, Rosenberger JR, et al. Nonresponding schizophrenia: differentiation by neurological soft signs and neuropsychological tests. *Schizophrenia bulletin.* 1999 Jan1;25(4):813-25.

51 Joober R, Rouleau GA, Lal S, et al. Neuropsychological impairments in neuroleptic-responder vs.-nonresponder schizophrenic patients and healthy volunteers. *Schizophrenia Research.* 2002 Jan15;53(3):229-38.

52 de Bartolomeis A, Balletta R, Giordano S, et al. Differential cognitive performances between schizophrenic responders and non-responders to antipsychotics: correlation with course of the illness, psychopathology, attitude to the treatment and antipsychotics doses. *Psychiatry research.* 2013 Dec15;210(2):387-95.

53 Bourque J, Lakis N, Champagne J, et al. Clozapine and visuospatial processing in treatment-resistant schizophrenia. *Cognitive Neuropsychiatry.* 2013 Nov1;18(6):615-30.

54 Frydecka D, Beszłej JA, Gościmski P, et al. Profiling cognitive impairment in treatment-resistant schizophrenia patients. *Psychiatry research.* 2016 Jan30;235:133-8.

55 Kravariti E, Demjaha A, Zanelli J, et al. Neuropsychological function at first episode in treatment-resistant psychosis: findings from the ÆSOP-10 study. *Psychological medicine.* 2019 Sep;49(12):2100-10.

56 Huang J, Zhu Y, Fan F, et al. Hippocampus and cognitive domain deficits in treatment-resistant schizophrenia: A comparison with matched treatment-responsive patients and healthy controls☆,☆☆,★,★★. *Psychiatry Research: Neuroimaging.* 2020 Mar30;297:111043.

57 Sosin D, Ivashchenko D, Sozaeva Z, et al. Cognitive impairment in patients with treatment resistant schizophrenia: Associations with DRD2, DRD3, HTR2A, BDNF and CYP2D6 genetic polymorphisms. *Neurology, Psychiatry and Brain Research.* 2019 Sep1;33:48-55.

58 Iasevoli F, Avagliano C, Altavilla B, et al. Evaluation of a few discrete clinical markers may predict categorization of actively symptomatic non-acute schizophrenia patients as treatment resistant or responders: a study by ROC curve analysis and multivariate analyses. *Psychiatry research.* 2018 Nov1;269:481-93.

59 Howes OD, McCutcheon R, Agid O, et al. Treatment-resistant schizophrenia: treatment response and resistance in psychosis (TRRIP) working group consensus guidelines on diagnosis and terminology. *American Journal of Psychiatry.* 2017 Mar1;174(3):216-29.

For peer review only - http://bmjopen.bmj.com/site/about/guidelines.xhtml

60 Chan SK, Chan HY, Honer WG, et al. Predictors of Treatment-Resistant and Clozapine-Resistant Schizophrenia: A 12-Year Follow-up Study of First-Episode Schizophrenia-Spectrum Disorders. *Schizophrenia Bulletin.* 2020 Oct12.

61 Zanelli J, Mollon J, Sandin S, et al. Cognitive change in schizophrenia and other psychoses in the decade following the first episode. *American Journal of Psychiatry.* 2019 Oct1;176(10):811-9.

62 Barder HE, Sundet K, Rund B, et al. Ten year neurocognitive trajectories in first-episode psychosis. *Frontiers in human neuroscience.* 2013 Oct7;7:643.

63 Folstein MF, Folstein SE, McHugh PR. "Mini-mental state": a practical method for grading the cognitive state of patients for the clinician. *Journal of psychiatric research.* 1975 Nov1;12(3):189-98.

64 Mathuranath PS, Nestor PJ, Berrios GE, et al. A brief cognitive test battery to differentiate Alzheimer's disease and frontotemporal dementia. *Neurology.* 2000 Dec12;55(11):1613-20.

65 Charernboon T, Chompookard P. Detecting cognitive impairment in patients with schizophrenia with the Addenbrooke's Cognitive Examination. *Asian journal of psychiatry.* 2019 Feb1;40:19-22.

For peer review only

STRATA: Investigating factors associated with response to antipsychotic treatment
Protocol. Version 4.0, 19th August 2016
REC REF: 15/LO/0038

STRATA: Investigating factors associated with response to antipsychotic treatment

Sponsor

Name of organisation: King's College London
Address: Room 1.8 Hodgkin Building, Guy's Campus, London SE1 4UL
Telephone: 020 7848 6960
Fax:
Email: keith.brennan@kcl.ac.uk

Chief Investigator

Name: Dr James MacCabe
Address: Institute of Psychiatry, Psychology & Neuroscience (IoPPN), De Crespigny Park, SE5 8AF
Telephone: 02078480757
Fax:
Email: james.maccabe@kcl.ac.uk

Name and address of Principal Investigator (on grant award).

Name: Prof Shitij Kapur
Address: Institute of Psychiatry, Psychology & Neuroscience (IoPPN), De Crespigny Park, SE5 8AF
Telephone: 020 7848 0593
Fax:
Email: shitij.kapur@kcl.ac.uk

Name and address of lead Co-Investigators at sites

Name: Prof Shon Lewis
Address: The University of Manchester, University Place, Oxford Road, Manchester, M13 9PL
Telephone: 0161 306 7944
Fax:
Email: shon.lewis@manchester.ac.uk

Name: Prof Mick O'Donovan
Address: MRC Centre for Neuropsychiatric Genetics and Genomics, Cardiff University, Henry Wellcome Building, Heath Park, Cardiff, CF14 4XN
Telephone: (029) 20687066
Fax:
Email: odonovanmc@cardiff.ac.uk

For peer review only

For peer review only - http://bmjopen.bmj.com/site/about/guidelines.xhtml

STRATA: Investigating factors associated with response to antipsychotic treatment
Protocol. Version 4.0, 19th August 2016
REC REF: 15/LO/0038

Name: Prof Stephen Lawrie
Address: University of Edinburgh, Royal Edinburgh Hospital, Edinburgh, EH10 5HF
Telephone: 0131 537 6671
Fax:
Email: s.lawrie@ed.ac.uk

For peer review only

For peer review only - http://bmjopen.bmj.com/site/about/guidelines.xhtml

STRATA: Investigating factors associated with response to antipsychotic treatment
Protocol. Version 4.0, 19th August 2016
REC REF: 15/LO/0038

CONTENTS

1. Introduction

2. Study Objectives and Design

2.1. Study Objectives

2.2 Study Design

3. Sample Size, Statistics, Selection and Withdrawal of Subjects

4. Study procedures

5. Sample Handling and Laboratories

6. Assessment of Safety

7. Study oversight arrangements

8. Ethics and Regulatory Approvals

9. Data Handling

10. Finance & Publication Policy

For peer review only

For peer review only - http://bmjopen.bmj.com/site/about/guidelines.xhtml

STRATA: Investigating factors associated with response to antipsychotic treatment
Protocol. Version 4.0, 19th August 2016
REC REF: 15/LO/0038

Study Synopsis

Full Title	Investigating factors associated with response to antipsychotic treatment
Short Title/Acronym	STRATA
Protocol Version number and Date	Version 4.0, 19th August 2016
Study Duration	36 months
Study Design	Basic Science
Sponsor/Co-sponsors	Kings College London / South London and Maudsley NHS Foundation Trust
Chief Investigator	Dr James MacCabe
REC number	15/LO/0038
Primary objective	The principle objective is to use Positron Emission Tomography (PET), Magnetic Resonance Spectroscopy (MRS), genetic and clinical data to confirm recent evidence of a distinct subtype of schizophrenia, based on differences in dopamine and glutamate function which would lead to developing a clinically useful, acceptable and cost-effective stratification tool.
Secondary objective (s)	To establish a lasting network of academia and industry partners and patients databases to facilitate and expedite both follow-up and novel research built to address patient stratification.<br><br>To examine and improve test-re-test and intercentre reliability of neuroimaging procedures for future large scale multisite studies in this mould that will be conducted.
Number of Subject	100
Main Inclusion Criteria	aged 18-65;<br><br>DSM 5 schizophrenia/schizophreniform disorder.

For peer review only - http://bmjopen.bmj.com/site/about/guidelines.xhtml

Statistical Methodology and Analysis	Summary statistics will be used to describe the demographic and clinical characteristics of each participant group.<br><br>Group differences in demographic, clinical variables and 18F-DOPAKi and glutamate concentration will be determined using pre-specified between group comparisons as appropriate (e.g. Chi square; Fischer's Exact; ANOVA).

For peer review only

For peer review only - http://bmjopen.bmj.com/site/about/guidelines.xhtml

STRATA: Investigating factors associated with response to antipsychotic treatment
Protocol. Version 4.0, 19th August 2016
REC REF: 15/LO/0038

1. Introduction

People with schizophrenia suffer from a range of symptoms including hallucinations (such as hearing voices), delusions (false beliefs) and thought disorder (thoughts not flowing in a logical way), as well as 'negative symptoms' such as a lack of motivation and withdrawal from social contact. Currently, antipsychotic medication is the mainstay of treatment for schizophrenia and all existing antipsychotic medications are thought to work by acting to reduce transmission of a brain chemical called dopamine. However, even after attempts to treat the disorder with two different antipsychotics, around 30% of patients still fail to improve. When this happens, the medical guidelines recommend treatment with a different drug called clozapine. However clozapine has several side effects and requires regular blood tests, so people do not like taking it. It is also ineffective in some patients.

The result is that a large number of patients spend too long on ineffective drugs which impact greatly on their mental health, well-being and quality of life whilst the cost of ineffective treatment is a huge financial burden to the NHS, consuming 25-50% of the total national mental health budget.

STRATA (funded by a £5M Medical Research Council award) aims to build on new evidence from neuroimaging and genetics studies suggesting that those who do not respond may actually have a completely different neurochemical abnormality causing their symptoms (the same sort of symptoms as are caused by excessive dopamine), involving a different chemical called glutamate. There are some new medicines under development that we hope will help people whose illness has not responded to standard medicines acting on dopamine.

We aim to develop a method to predict, even as early as when first seen, which patients will respond to standard dopamine drugs, and which people are instead more likely to respond to the new glutamate drugs. This will allow people to receive the medicines they need straight away, without having to try ineffective drugs first.

The proposed research programme is broken down into several parts. This protocol describes the first study, which is a UK, multicentre study using brain scans to confirm that those patients who don't respond to standard treatments have higher glutamate levels, but lower dopamine levels than those who respond well. This information, along with clinical and genetic information, will be used to develop tests to identify in advance which people will respond to dopaminergic versus glutamatergic medication.

2. Study Objectives and Design

2.1. Study Objectives and Outcomes

The principle objective is to use Positron Emission Tomography (PET), Magnetic Resonance Spectroscopy (MRS), genetic and clinical data to confirm recent evidence of a distinct subtype of schizophrenia, based on differences in dopamine and glutamate function which would lead to developing a clinically useful, acceptable and cost-effective stratification tool.

The secondary research objectives are:

For peer review only - http://bmjopen.bmj.com/site/about/guidelines.xhtml

i) To establish a lasting network of academia and industry partners and patient databases to facilitate and expedite both follow-up and novel research built to address patient stratification.

ii) To examine and improve test-re-test and intercentre reliability of neuroimaging procedures for future large scale multisite studies in this mould that will be conducted.

The study is designed to generate a predictive test for treatment response so the outcome will be the overall measure generated. The data that will lead to this will include MRS glutamate level, the PET Ki value, polygenic risk score and clinical variables such as PANSS score.

2.2 Study Design

STRATA is a multi centred study. 100 participants will be recruited across 4 university research sites including KCL, University of Manchester, Cardiff University, and University of Edinburgh.

Participants will consent to all aspects of the study including interviews/assessments, blood and urine sampling, MRI scan and PET scan (the latter in London and Manchester only) but can also choose to opt out of some tasks if necessary.

1. Assessments

An initial interview will collect demographic and personal information (e.g. address, contact details, date of birth, gender, handedness, head injury and other relevant medical history), and structured assessments of medication history and response. Clinical information will also be recorded from medical records. The Mini International Neuropsychiatric Interview (MINI) will be used to confirm diagnosis, which takes around 15 minutes to complete.

Illness severity will be measured using:

i. Positive and Negative Syndrome Scale (PANSS),
ii. Clinical Global Impression scale (CGI -SCH)
iii. Kemp Clinician Rating Scale (of adherence to treatment)
iv. Brief Assessment of Cognition in Schizophrenia (BACS)

2. Biological samples

Blood samples will be collected via cannula (as described under the PET scan section below) or by venous puncture, during a routine blood sample whenever possible. The participant will give up to 50ml in blood (around 3 tablespoons), this is in line with sampling guidelines.

While the biological sample collection is ongoing, samples will be stored at the laboratory corresponding to each research site. The samples for genetics analysis will subsequently be transferred to the MRC Center for Neuropsychiatric Genetics and Genomics, Cardiff University.

KCL/South London and the Maudsley (SLaM) participants will also be invited to participate in the BRC Biobank. This is covered by a separate ethical approval (09/H0606/84 NRES Committee South Central-Oxford C.)

For peer review only - http://bmjopen.bmj.com/site/about/guidelines.xhtml

For peer review only

STRATA: Investigating factors associated with response to antipsychotic treatment
Protocol. Version 4.0, 19th August 2016
REC REF: 15/LO/0038

Participants will be asked to provide samples (urine and blood) for metabolomics analysis. This will be processed at MRC-NIHR National Phenome Centre. The Centre is funded by the MRC and NIHR and led by Imperial College London and King's College London.

As of April 2016, participants will also have a sample taken for proteomic analysis. These samples will be sent to the University of Manchester (Molecular Pathology Innovation Centre). This will be within the 50ml sampling guideline already approved.

3. Magnetic Resonance Imaging (MRI)

The MRI scans (100 in total) will take place at four locations (London, Cardiff, Manchester and Edinburgh) at NHS Trust or University sites. The MRI session will last a maximum of 1 hour. During the scan, participants will be asked to lie flat on their back with their head inside the scanner. The scanner makes a loud noise as it takes pictures, so participants will be given headphones to wear and asked to lie as still as possible. The researcher will be able to speak to the participant over the microphone throughout and participants will be told if they do feel uncomfortable the session can be stopped at any time. The MRI scan itself is painless and safe. Some people find scans claustrophobic or anxiety-provoking, and we have a mock scanner that participants can try out first. The scanner consists of a powerful magnet, which may attract metal objects. Therefore before the scan participants will go through a safety questionnaire, to check that they can have the scan. If a participant has any metal in their body, either from accidents or operations, they may not be able to have the MRI scan, but they can still take part in the rest of the study.

All data collection will occur at 3 Tesla. During the scan, data acquisition will include acquisition of localizer, T1-weighted and T2-weighted structural scans. 1H-MRS data for measurement of regional concentrations of glutamate and other metabolites present in the 1H-MRS spectra will be acquired using conventional PRESS (Point RESolved Spectrocopy) acquisition routines, as well as a resting state fMRI sequence if time allows.

Due to change of scanner at Cardiff University, participants recruited in Cardiff prior to the decommissioning of the old scanner will be re-contacted and asked whether they would volunteer for a second MRI scan on the new scanner. They will also be asked to repeat some of the interview/assessments and may be asked for biological samples (only in circumstances where these were not provided previously). Participants will be reimbursed for their time at the same rate.

In the unlikely event that MRI scanner issues or excessive movement make the MRS data unusable at other sites, participants can be re-contacted and asked whether they would like to volunteer for a second scan.

4. Positron Emission Tomography (PET)

The PET scans (60 in total, subset of those having MRI scans) will take place at two sites:
i) Imanova Limited, Imperial College London, Hammersmith Hospital in London.
ii)The Wolfson Molecular Imaging Centre in Manchester.

PET with the radiotracer 18F-DOPA will be used to assess brain dopaminergic function in a sub-set of participants (N=60) recruited at KCL and University of Manchester. The PET scan procedure involves an initial transmission scan followed by a dynamic scan lasting approximately

For peer review only - http://bmjopen.bmj.com/site/about/guidelines.xhtml

90 minutes after injection of the radiotracer 18F DOPA through a cannula inserted into an arm vein. In the event the participant has to get off the scanner e.g. to go to the toilet or for some other reason then the transmission scan may be repeated to reposition them in the camera. In the unlikely event of technical failure prior to or during the PET scan the subject will be invited back for a replacement session (the total dose will then be ~7.5mSv, and the risks of this will be explained).

In Manchester, participants will be offered the option of having an extra, High Resolution Research Tomograph (HRRT) scan after their main STRATA PET scan (after a 15 minute comfort break). This will be between 30-60 minutes depending upon participant tolerability. Due to the long half-life of 18F and the slow removal of 18F from the brain, this extra scan will not involve any further injection of radiotracer. Another transmission scan will be carried out for attenuation correction purposes although this will be of very low radiation dose (0.02mSv). In the event of significant head movement during the HRRT scan, this transmission scan may be repeated.

In order to minimise the peripheral breakdown of 18F-DOPA, an oral dose of 150mg carbidopa and 400mg entacapone will be given one hour prior to the scan. Very few people experience any side-effects from these. Very occasionally people experience stomach upset, muscle movements, dry mouth and/or an orange tinge to their urine from the tablets, which may last a few hours to a day. This permits the use of a lower dose of 18F-DOPA than would otherwise be necessary. Participants will be asked to refrain from eating, drinking (apart from water) and smoking from midnight on the night before the scan, until after the scan is finished. This is because large amino acids may affect brain uptake of 18F-DOPA. Participants will also be instructed not to take illicit drugs (such as cannabis or cocaine) in the prior three days. Before the scan we will ask for a urine sample to check whether substances that can affect the scan are in their system. Women of childbearing age will have a pregnancy test and will be required to use regular contraception prior to the scan. At the start of the scan we will give participants a radiotracer (which is mildly radioactive) to measure the brain dopamine system. At the end of the scan the cannula will be removed from their arm.

Participants taking part in a PET scan at Imanova (SLaM/KCL participants) will have an additional 1-2 tablespoons (up to 30ml) of blood taken through their cannula to measure natural blood chemicals (hormones and genes) that are connected to dopamine function.

Participants taking part in a PET scan in Manchester will have all their bloods taken at this point (up to 50ml) as described under 'Biological Samples Section', whenever possible.

3. Sample Size, Statistics, Selection and Withdrawal of Subjects

The patients will be identified by members of the clinical team. Only the clinical team (who may also be part of the research team with NHS honorary contracts) will be able to access participant records and data prior to consent. No patient records will be screened by study researchers prior to consent. Study researchers will have access to patient records after/ if participants have consented to this.

We will recruit a total of 100 participants. Potential participants may be referred via clinical teams or other research studies/existing databases with consent to re-contact or registries and recruitment initiatives in NHS Trusts whose terms are in accordance with NHS Trust policies.

For peer review only - http://bmjopen.bmj.com/site/about/guidelines.xhtml

STRATA: Investigating factors associated with response to antipsychotic treatment
Protocol. Version 4.0, 19th August 2016
REC REF: 15/LO/0038

Participants will be first approached by a member of the clinical team or a member of the research team, with approval from the clinical team/ other study

Inclusion Criteria

1) aged 18-65;
2) DSM 5 schizophrenia/schizophreniform disorder.
3) Participants must read and write in English at a level sufficient to understand and complete study-related procedures

Exclusion Criteria

1) Pregnancy;
2) Severe head injury involving loss of consciousness >5 minutes (ever);
3) Meeting ICD criteria for harmful substance misuse or psychotic disorder secondary to substance misuse;
4) Participation in MRI scans requires exclusion of contraindications to MRI at 3 tesla e.g. metallic or electronic implants;
5) Severe claustrophobia.
6) Treatment with clozapine in the last 3 months

For peer review only

To establish and confirm the stratifier 1H-MRS data will be acquired in a total of 100 patients early in the course of their treatment; 50 T-Resp and 50 T-NonResp; matched for chronicity of illness.

Operational definition of T-Resp:
(i) treatment with only one antipsychotic drug since onset, or treatment changes have been due to adverse effects, not for non-response. (ii) CGI-SCH severity score of <4; (iii) PANSS total <60 (Leucht 2005); (iv) CRS >3

Operational definition of T-NonResp:
(i) documented treatment with at least two antipsychotics each above the minimum therapeutic dose as defined by the BNF for >4 weeks each; (ii) despite ongoing treatment and adequate adherence (assessed by iv) a CGI-SCH severity score of >3; (iii). PANSS total severity rating of at least 70 iv) Clinician Rating Scale (CRS; a measure of adherence) (Kemp et al 1996) >3.

Power and sample size calculation:
The study is powered to give >95% power to detect differences found in Egerton et al 2012 (α=0.05, allowing for 10% loss of sensitivity due to combining data from multiple centres). We have more than 80% power to detect a significant difference between a ROC curve with AUC 0.7 and chance, assuming α=0.05, 2-tailed. Two-tailed 18F-DOPA PET data will be acquired in a subset at 2 sites (N=60) to determine if the double dissociation between DA function and GLU function we have seen in chronic patients is also evident early in the illness course, where the strategy is most likely to be used (T-Resp n=30, T-NonResp n=30; powered to give >95% power to detect differences found in Demjaha et al 2012; α=0.05, allowing for 10% loss of sensitivity due to combining data from multiple centres).

Summary statistics will be used to describe the demographic and clinical characteristics of each participant group.

For peer review only - http://bmjopen.bmj.com/site/about/guidelines.xhtml

Group differences in demographic, clinical variables and 18F-DOPA Ki and glutamate concentration will be determined using pre-specified between group comparisons as appropriate (e.g. Chi square; Fischer's Exact; ANOVA).

Missing data will be minimal given that data is being collected prospectively. The exact reason for the missing data will be recorded. Any blank measures or spurious data will be checked against the paper copy of the CRF stored securely at sites.

Participants will be clearly told they can withdraw from the study at any time without having to give a reason. This is clear in the information sheet and the researcher will also explain this verbally to participants during the informed consent process. If a participant wishes to withdraw from a study all their identifiable data will be destroyed. Data or tissue already collected with the consent, which is not identifiable, would be retained and used in the study. No further data or tissue would be collected or any other research procedures carried out on or in relation to the participant.

Control group

We will recruit up to 15 healthy volunteers aged 18-65 to be scanned at each PET site (two sites; Imanova Limited, Imperial College London and The Wolfson Molecular Imaging Centre in Manchester) and 10 healthy volunteers aged 18-65 to be scanned at each MRI site (4 sites). This is to determine inter-site scanner variability and to provide normal range data for comparison with the clinical groups. In addition to the exclusion criteria above, healthy volunteers will be excluded if there is a history of schizophrenia or other psychotic disorder. Healthy volunteers will be recruited using an existing database of interested potential participants held at KCL.

4. Study procedures

Informed Consent

1) Participants will be first approached by a member of the clinical team or a member of the research team, with approval from the clinical team.
2) The study will be described verbally to potential participants and they will be given a copy of the information sheet. They will be encouraged to ask questions about the research. Potential participants will be allowed as much time as they require to make a decision and at least 24 hours so they are able to seek advice from others about participation, including previous participants in the research where possible.
3) If a patient expresses an interest in taking part, capacity to consent will be assessed and documented by the research team, in consultation with the clinical team.
4) If the patient has capacity to consent and agrees to participate in the study, they will be asked to sign and date two copies of the consent form. One copy will be kept by the participant and one by the research team. The research team will pass onto the clinical team to scan into medical notes, or incorporate in paper notes.
5) The participant will be informed that they can withdraw consent at any time, and without giving a reason.
6) Participants will be informed they are to be compensated for their time and travel expenses. This monetary amount will be up to £120 (£145 in Manchester) depending on which parts of the study the participant is involved with.

For peer review only - http://bmjopen.bmj.com/site/about/guidelines.xhtml

STRATA: Investigating factors associated with response to antipsychotic treatment
Protocol. Version 4.0, 19th August 2016
REC REF: 15/LO/0038

Within Avon and Wiltshire Mental Health Partnership NHS Trust, Everyone Included will be used to identify potentially eligible participants. Potentially eligible participants are identified based on the study inclusion /exclusion criteria, excluding those who have declined to receive information. This is done via an automated search of the Trust's electronic patient record system (RiO). An authorised search will be requested by a member or the R&D department, who are part of the clinical team and carried out by a member of the Information Analysis team. A data set is returned directly to the Everyone Included Administrators for processing the letters. No patient identifiable data will ever leave the Trust or be accessed by an external research team during this process.

The 'Research Opportunity Letter' will be sent to these individuals. The letter itself will not contain any patient identifiable or disclosing information (such as making reference to their diagnosis or medications). It will provide a free-post return slip and contact details (phone, email, website, postal address) inviting individuals to get in touch if they would like to further information / to take part. The onus is on the individual to express an interest, otherwise no further action is taken.

Upon responding to the 'Research Opportunity Letter', a Participant Information Sheet will be provided. If the research team is external, the individual will be asked if they are happy for their details (i.e. name and phone number) to be passed directly to the research team. No information is ever accessed by or passed to an external research team without first gaining permission from the potential participant. At this point standard study recruitment processes proceed.

Risks and burdens

The questionnaires involve personal questions and recalling experiences that some people may find distressing. Participants will be told if they feel uncomfortable with any of the questions they do not have to answer them.

Blood sampling and placing the cannula can cause some discomfort, and there is a possibility that a small bruise may develop. This task will be performed by research workers trained in phlebotomy. Any risks of infection will be contained by using standard sterile procedures and the risks associated with this task will be the same as for any other blood sample collection.

Any participants who become distressed during any procedure involved in this study will be encouraged to pause and will be reminded routinely that they can withdraw from the study at any time without a reason or penalty.

Any clinically significant issues that may arise during the assessment, the verbal consent will be obtained from the patient to pass onto the responsible psychiatrist or other relevant member of the staff. This will always be done with the participants' permission and will only be breached in the rare cases when there is judged to be an issue of safety, for example if the participant makes specific threats towards an individual.

Imaging

The MRI and PET scans themselves are painless and safe. Some people find the scans claustrophobic or anxiety-provoking. There is a mock scanner that participants can try out first if they wish. Participants will be told if they feel uncomfortable the scanning can be stopped at any time. Before the scan we will go through a safety questionnaire, to check that participants can have the scan. If they have any metal in their body, either from accidents or operations, they may

For peer review only - http://bmjopen.bmj.com/site/about/guidelines.xhtml

not be able to have the MRI scan, but they can still take part in the rest of the study. Clinical Research workers and research workers will log screening results.

Very occasionally people experience side effects from the medication they receive when taking part in a PET scan. These side-effects can include stomach upsets, muscle movements, dry mouth and/or an orange tinge to their urine. These side-effects may last a few hours to a day but participants will be warned about this and told it is nothing to worry about.

PET scans involve a small amount of radiation. Any exposure to radiation carries a risk of damaging the body's tissues and possibly triggering cancer at a later date. However, the risk is very small. A standard PET scan in this study will expose participants to 3.7mSv, (this may be 3.72mSv in Manchester if participants decide to have the extra, high resolution PET scan), which is the same amount of radiation that they are exposed to from natural sources of radiation, such as the sun, over the course of 18 months. In extremely rare cases the PET scan may need to be repeated and we have ARSAC approval for a maximum of 7.5mSv exposure per participant. Most experts believe that the risk of cancer developing only becomes significant in people who are exposed to 100mSv or more. However, as a precaution we are excluding pregnant or breastfeeding women. A pregnancy test will be carried out on female urine samples before the PET scan is conducted. Participants will be asked to consent to this on the consent form. Clinical Research workers and research workers will log screening results and ensure participants will not be exposed to more than 10mSv in 12 months (ARSAC guidelines suggest 10mSv as the normal upper limit for radiation exposure related to research procedure)

5. Sample handling and laboratories

Biological sample collection tubes and barcodes will be sent to sites in advance from the MRC Center for Neuropsychiatric Genetics and Genomics, Cardiff University. Samples will be stored in laboratories at sites and transportation will be organized when required (likely at 6 monthly basis, dependent on recruitment). Details of sample collection and storage at site will be recorded. Study SOPs will describe collection and storage specifications to ensure all sites are following the same guidelines.

When samples arrive at Cardiff University, researchers will ensure that the physical integrity of these samples have not been compromised in transit and track the samples in using their barcodes. The research team at Cardiff will notify the sponsor and the other study teams of any issues in transportation.

Cardiff University will extract DNA from the blood. We will perform genome-wide and targeted genotyping and/or exome or whole genome sequencing. We will seek genetic association with the imaging and other outcome measures at the level of individual genotype/sequence variant, genes, gene sets/pathways and polygenic or other summary scores.

A urine and blood sample will undergo metabolomic analysis at MRC National Phenome Centre. The Centre is funded by the MRC and NIHR and led by Imperial College London and King's College London. An additional blood sample will undergo proteomic analysis at the University of Manchester.

KCL/South London and the Maudsley (SLaM) participants will also be invited to participate in the BRC Biobank. This is covered by a separate ethical approval (09/H0606/84 NRES Committee South Central-Oxford C.)

For peer review only - http://bmjopen.bmj.com/site/about/guidelines.xhtml

STRATA: Investigating factors associated with response to antipsychotic treatment
Protocol. Version 4.0, 19th August 2016
REC REF: 15/LO/0038

6. Assessment of Safety

There are no serious adverse events expected to occur during the study.

All blood samples will only be taken by researchers trained in phlebotomy. All risks are the same as for any routine blood sample and are therefore minimal.

The drugs administered and the radiotracer used for the PET are standard procedures. The drugs administered may cause stomach upsets, muscle movements, dry mouth and/or an orange tinge to their urine. These side-effects may last a few hours to a day but participants will be warned about this and told it is nothing to worry about. Female participants will have a pregnancy test in advance.

For MRI scans a safety questionnaire will be carried out prior to the scan to check the participant does not have any metal in their bodies from operations or accidents.

7. Study oversight arrangements

STRATA is a multi-centred study and this will be managed by attendance at a monthly Consortium Executive meeting which will be responsible for the effective oversight of the daily activities of the study. Quarterly Consortium Board (CB) teleconferences will oversee the progress of, and interaction between, the workstreams to maintain communication of issues and progress between sites across the different aspects of STRATA. The CB will submit six-monthly Programme reports to the funder, MRC.

The project team consists of a full time Project Manager based at the IoPPN, KCL and a 50% Project Manager at the University of Manchester.

8. Ethics & Regulatory Approvals

REC name and address: South East Coast-Surrey Research Ethics Committee, Whitefriars, Level 3, Block B, Lewins Mead, Bristol, BS1 2NT

This study has also been reviewed and approved by the Administration of Radioactive Substances Advisory Committee (ARSAC).

9. Data Handling

Once participants have consented to be in the study some personal details will be taken. These details will be taken by the researcher with full consent to do so. These details will be kept securely at sites and used to contact patients when required to make appointments. No personal data will be shared with anyone outside of that study team. Each participant will be given a unique identifier and any clinical or genetic or imagining data relating to the same participant will link via that code.

Data will be entered and stored on a secure web application called Research Electronic Data Capture (REDCap). REDCap will not store any personal details and all participants will have a unique non-identifiable ID code. This unique ID code will then be used to merge all processed imaging, genetics and clinical data. REDCap will be hosted on secure servers at the Biomedical

For peer review only - http://bmjopen.bmj.com/site/about/guidelines.xhtml

Research Centre at Kings College London. All sites can access REDCap for the purposes of data entry via a web browser and data is uploaded when a WIFI signal is available.

The CI will ensure that the trial is conducted in compliance with the principles of the Declaration of Helsinki (1996), and in accordance with all applicable regulatory requirements including but not limited to the Research Governance Framework, Trust and Research Office policies and procedures and any subsequent amendments.

10. Finance and Publication Policy

STRATA is funded by a £ £4,900,000 Medical Research Council grant. Kings College London will receive and manage this funding. A collaboration agreement has agreed budgets between sites.

Analysis and findings from the study will be published as papers in journals. No identifiable data will be included.

This study has been adopted onto the UKCRN Portfolio and the research project will be registered on their database which is publicly available.

For peer review only

For peer review only - http://bmjopen.bmj.com/site/about/guidelines.xhtml

BMJ Open

A cross sectional study comparing cognitive function in treatment responsive versus treatment non-responsive schizophrenia: evidence from the STRATA study.

Journal:	*BMJ Open*
Manuscript ID	bmjopen-2021-054160.R1
Article Type:	Original research
Date Submitted by the Author:	14-Aug-2021
Complete List of Authors:	Millgate, Edward; King's College London Institute of Psychiatry Psychology and Neuroscience, Department of Psychosis Studies<br>Kravariti, Eugenia; King's College London Institute of Psychiatry Psychology and Neuroscience, Department of Psychosis Studies; NIHR Biomedical Research Centre at Guy's and Saint Thomas' NHS Foundation Trust and King's College<br>Egerton, Alice; King's College London Institute of Psychiatry Psychology and Neuroscience, Department of Psychosis Studies; NIHR Biomedical Research Centre at Guy's and Saint Thomas' NHS Foundation Trust and King's College<br>Howes, Oliver; King's College London Institute of Psychiatry Psychology and Neuroscience, Department of Psychosis Studies; NIHR Biomedical Research Centre at Guy's and Saint Thomas' NHS Foundation Trust and King's College<br>Murray, Robin; King's College London Institute of Psychiatry Psychology and Neuroscience, Department of Psychosis Studies; NIHR Biomedical Research Centre at Guy's and Saint Thomas' NHS Foundation Trust and King's College<br>Kassoumeri, Laura; King's College London Institute of Psychiatry Psychology and Neuroscience, Department of Psychosis Studies; NIHR Biomedical Research Centre at Guy's and Saint Thomas' NHS Foundation Trust and King's College<br>Donocik, Jacek; King's College London Institute of Psychiatry Psychology and Neuroscience, Department of Psychosis Studies<br>Lewis, Shon; The University of Manchester Division of Psychology and Mental Health; Greater Manchester Mental Health NHS Foundation Trust<br>Drake, Richard; The University of Manchester Division of Psychology and Mental Health; Greater Manchester Mental Health NHS Foundation Trust<br>Lawrie, Stephen; The University of Edinburgh Division of Psychiatry, Psychiatry<br>Murphy , Anna; The University of Manchester Division of Psychology and Mental Health; Greater Manchester Mental Health NHS Foundation Trust<br>Collier, Tracy; King's College London Institute of Psychiatry Psychology and Neuroscience, Department of Psychosis Studies<br>Lees, Jane; The University of Manchester Division of Psychology and Mental Health; Greater Manchester Mental Health NHS Foundation Trust<br>Stockton-Powdrell, Charlotte; The University of Manchester Division of Psychology and Mental Health<br>Walters, James; Cardiff University

	Deakin, JFW; The University of Manchester Division of Psychology and Mental Health<br>MacCabe, James; King's College London Institute of Psychiatry Psychology and Neuroscience, Department of Psychosis Studies; NIHR Biomedical Research Centre at Guy's and Saint Thomas' NHS Foundation Trust and King's College
**Primary Subject Heading**:	Mental health
Secondary Subject Heading:	Pharmacology and therapeutics
Keywords:	Schizophrenia & psychotic disorders < PSYCHIATRY, PSYCHIATRY, Adult psychiatry < PSYCHIATRY

SCHOLARONE™ Manuscripts

I, the Submitting Author has the right to grant and does grant on behalf of all authors of the Work (as defined in the below author licence), an exclusive licence and/or a non-exclusive licence for contributions from authors who are: i) UK Crown employees; ii) where BMJ has agreed a CC-BY licence shall apply, and/or iii) in accordance with the terms applicable for US Federal Government officers or employees acting as part of their official duties; on a worldwide, perpetual, irrevocable, royalty-free basis to BMJ Publishing Group Ltd ("BMJ") its licensees and where the relevant Journal is co-owned by BMJ to the co-owners of the Journal, to publish the Work in this journal and any other BMJ products and to exploit all rights, as set out in our licence.

The Submitting Author accepts and understands that any supply made under these terms is made by BMJ to the Submitting Author unless you are acting as an employee on behalf of your employer or a postgraduate student of an affiliated institution which is paying any applicable article publishing charge ("APC") for Open Access articles. Where the Submitting Author wishes to make the Work available on an Open Access basis (and intends to pay the relevant APC), the terms of reuse of such Open Access shall be governed by a Creative Commons licence – details of these licences and which Creative Commons licence will apply to this Work are set out in our licence referred to above.

Other than as permitted in any relevant BMJ Author's Self Archiving Policies, I confirm this Work has not been accepted for publication elsewhere, is not being considered for publication elsewhere and does not duplicate material already published. I confirm all authors consent to publication of this Work and authorise the granting of this licence.

For peer review only

Journal: BMJ Open

Word count: 2996 *(Table word count = 490)*

Abstract word count: 293

Submission date: 3rd June 2021

A cross sectional study comparing cognitive function in treatment responsive versus treatment non-responsive schizophrenia: evidence from the STRATA study.

Edward Millgate[1], Eugenia Kravariti[1,2], Alice Egerton[1,2], Oliver D Howes[1,2], Robin M Murray[1,2], Laura Kassoumeri[1,2], Jacek Donocik[1], Shôn Lewis[3,4], Richard Drake[3,4], Stephen M Lawrie[5], Anna Murphy[3,4], Tracy Collier[1], Jane Lees[3,4], Charlotte Stockton-Powdrell[3], James Walters[6], Bill Deakin[3,4] and James H MacCabe[1,2]*

[1] Institute of Psychiatry, Psychology and Neuroscience, King's College London, United Kingdom

[2] NIHR Biomedical Research Centre at South London and Maudsley NHS Foundation Trust, London, United Kingdom

[3] Division of Psychology and Mental Health, School of Biological Sciences, Faculty of Biology, Medicine and Health, University of Manchester, Manchester

[4]Greater Manchester Mental Health NHS Foundation Trust, Manchester, United Kingdom

[5]Division of Psychiatry, University of Edinburgh, Edinburgh, United Kingdom

[6]Division of Psychological Medicine and Clinical Neuroscience, Cardiff University, Cardiff, United Kingdom

*Corresponding author: james.maccabe@kcl.ac.uk

Department of Psychosis Studies,

Institute of Psychiatry, Psychology & Neuroscience,

16 de Crespigny Park,

Denmark Hill, London,

United Kingdom, SE5 8AF

For peer review only - http://bmjopen.bmj.com/site/about/guidelines.xhtml

Abstract

Background: 70-84% of individuals with antipsychotic treatment resistance show non-response (NR) from the first episode. Emerging cross-sectional evidence comparing cognitive profiles in treatment resistant schizophrenia to treatment-responsive schizophrenia has indicated that verbal memory and language functions may be more impaired in treatment resistance. We sought to confirm this finding by comparing cognitive performance between antipsychotic non-responders (NR) and responders (R) using a brief cognitive battery for schizophrenia, with a primary focus on verbal tasks compared against other measures of cognition.

Design: Cross-sectional

Setting: This cross-sectional study recruited antipsychotic treatment responders (R) and antipsychotic non-responders (NR) across four UK sites. Cognitive performance was assessed using the Brief Assessment of Cognition in Schizophrenia (BACS).

Participants: One hundred and six participants aged 18 – 65 years with a diagnosis of schizophrenia or schizophreniform disorder were recruited according to their treatment response, with 52 NR, and 54 R cases.

Outcomes: Composite and subscale scores of cognitive performance on the BACS. Group (R vs NR) differences in cognitive scores were investigated using univariable and multivariable linear regressions adjusted for age, gender and illness duration.

Results: Univariable regression models observed no significant differences between R and NR groups on any measure of the BACS, including verbal memory (95% CI -6.63 to 2.66, $p = .398$) and verbal fluency (95% CI -2.46 to 4.91, $p = .510$). This pattern of findings was consistent in multivariable models.

Conclusions: The lack of group difference in cognition in our sample is likely due to a lack of clinical distinction between our groups. Future investigations should aim to utilise machine learning methods using longitudinal first episode samples to identify responder subtypes within schizophrenia, and how cognitive factors may interact within this.

Trail registration number: REC: 15/LO/0038.

Keywords: cognition, treatment resistance, antipsychotic response, schizophrenia, BACS

For peer review only - http://bmjopen.bmj.com/site/about/guidelines.xhtml

Article summary

Strengths and limitations of this study

- The study examined cognitive performance in a relatively large and multicentre sample of antipsychotic responders and non-responders
- Cognition was assessed with the BACS, a reliable and brief test battery specifically designed for schizophrenia
- The lack of significant group differences in cognition between antipsychotic responders and non-responders may reflect limited clinical separation between these groups.

For peer review only

For peer review only - http://bmjopen.bmj.com/site/about/guidelines.xhtml

Introduction

Up to a third of patients with a schizophrenia diagnosis have inadequate symptomatic improvement despite having at least two antipsychotic drugs, one being a second-generation antipsychotic excluding clozapine, at adequate doses and duration (4 – 6 weeks; NICE guidelines) [1] and are termed treatment resistant (TRS) [2,3]. Almost all guidelines recommend the antipsychotic clozapine in TRS [4] with earlier clozapine treatment associated with better outcomes [5-8]. There is increasing evidence that TRS may represent a distinct subtype in schizophrenia [9,10]. Most treatment resistant cases exhibit antipsychotic non-response (NR) from the first episode with this observed in 70-84% of patients [3,11]. An earlier age of onset has also been consistently associated with antipsychotic treatment resistance [12-16], suggesting that TRS and NR may be associated with neurodevelopmental impairment. Identifying these underlying factors associated with antipsychotic treatment resistance in schizophrenia is therefore important for improving prediction and early treatment of NR and TRS.

Cognitive impairment in schizophrenia may provide some insight into antipsychotic treatment response. Performance on tasks of verbal memory has often been reported to be impaired in schizophrenia samples [17], those prior to medication initiation [18], and at first episode [19,20]. Indeed, impairments in verbal memory and language functions have also been reported in unaffected first-degree relatives of schizophrenia patients relative to healthy controls [21,22]. Verbal memory and verbal working memory functions have also been reported to show a protracted maturation into adulthood, with impairments in these functions observed in both early and late schizophrenia [23]. This suggests a possibility of a genetic and cognitive continuum of risk in schizophrenia, which increases from controls to first-degree relatives, to treatment responsive schizophrenia. A broader hypothesis is that treatment resistance is etiologically continuous with treatment responsive schizophrenia but occupies a more exaggerated position on a continuum of neurodevelopmental liability.

In a recent meta-analysis comparing mostly cross-sectional studies of treatment resistant cases and responders, TRS cases exhibited greatest cognitive impairments on tasks of verbal memory and learning (dl = -0.59, p <.001) and language functions (dl = -0.53, p <.001), with smaller but still statistically significant impairments in tasks across other cognitive domains, relative to their responder counterparts [24]. However, this meta-analysis included an array of cognitive tasks, many with long test duration and stringent training

For peer review only - http://bmjopen.bmj.com/site/about/guidelines.xhtml

requirements for raters. Short and comprehensive measures of cognitive performance may aid in the detection of neuropsychological differences between antipsychotic responders (R) and non-responders (NR), while also being cost-effective. The Brief Assessment of Cognition in Schizophrenia (BACS) [25] was originally developed to be an easily administrable, brief, test battery that efficiently and specifically assesses cognitive deficits in schizophrenia cases. The measures included in the battery correspond to several cognitive domains with established deficits in schizophrenia; executive functions [26,27], working memory [28,29], motor/processing speed [30], verbal memory [31,32], verbal fluency [33,34] and attention [35,36]. If observable differences between antipsychotic responders and non-responders are identified, this would further improve our understanding of cognitive factors implicated in the aetiology of antipsychotic response. Likewise, this would raise the possibility for future prospective research to use brief cognitive testing as part of predictive/diagnostic models for antipsychotic response and future treatment resistance.

Therefore, this cross-sectional study sought to assess the cognitive profiles of antipsychotic responders and non-responders utilising the Brief Assessment of Cognition in Schizophrenia. Based on the existing literature, we hypothesised that TRS patients would have poorer performance across BACS tasks, particularly on verbal memory and verbal fluency measures.

Methods

Design

The study used a cross-sectional design comparing antipsychotic treatment responders (R) and antipsychotic non-responders (NR) on cognitive performance.

Setting

The study was part of 'Schizophrenia: Treatment Resistance and Therapeutic Advances' (STRATA), a consortium which included King's College London (London, UK), University of Manchester (Manchester, UK), Cardiff University (Wales, UK) and University of Edinburgh (Scotland, UK). The aim of the STRATA consortium is to identify neurobiological, cognitive and genetic biomarkers of antipsychotic treatment resistance and non-response within schizophrenia and other related psychotic disorders.

For peer review only - http://bmjopen.bmj.com/site/about/guidelines.xhtml

Patient and Public Involvement

During the early development of the study the views and recommendations of service users and carers regarding the use of stratified medicine research were assessed. Consultations were undertaken with the Institute of Psychiatry, Psychology and Neuroscience's Service User Advisory Group (SUAG). Service user researchers in London, Manchester, and Edinburgh (18 people) carried out focus groups, and one carer focus group in London (8 people). Focus groups were digitally recorded, the transcripts analysed in NVivo 10 using a simple thematic analysis, and quotations de-identified to protect participants. The results of this research are published in BioMed Central [37]. Both service users and carers reflected enthusiasm for stratified medicine. Each stage of the study was discussed, including their willingness to participate and attitudes towards, and perceived intrusiveness of different procedures. These individuals also aided in commenting and providing recommendations on consent and participant information forms.

Participants

One hundred and six participants were recruited following a screening of patients across four sites: King's College London (N = 38), University of Manchester (N = 32), Cardiff University (N = 16) and University of Edinburgh (N = 18). Inclusion criteria were as follows: aged 18 – 65 years, with a schizophrenia or schizophreniform disorder diagnosis as per Diagnostic and Statistical Manual of Mental Disorders, 5th edition (DSM-5) [38] criteria and be able to read and write English to a sufficient level (see also Egerton et al [39]). Participants were excluded if they were pregnant, had ever experienced a head injury involving loss of consciousness for more than 5 minutes, met ICD criteria for harmful substance misuse or a psychotic disorder secondary to substance use, scored < 3 on the Clinical Rating Scale (a measure of adherence) [40,41], or had been treated with clozapine in the previous three months. All participants gave informed consent prior to enrolment. This study was approved by the South East Coast-Surrey Research Ethics Committee; REC: 15/LO/0038. All participants provided informed consent prior to participation.

Definition of antipsychotic response and antipsychotic non-response

For peer review only - http://bmjopen.bmj.com/site/about/guidelines.xhtml

Participants were defined as antipsychotic treatment responders (R) if they had been treated with only one antipsychotic drug since illness onset or if their antipsychotic drug had been changed only for reasons of adverse effects as opposed to non-response. In addition to this, responders needed to have a Clinical Global Impression score (CGI-SCH) [42] of below 4 (*moderately ill*), a Positive and Negative Syndrome scale (PANSS) [43] total score below 60, and a Clinical Rating Scale (CRS) [40,41] level of adherence greater than 3 (‘*accepts only because compulsory*’). Fifty-four treatment responders were recruited into the study.

Antipsychotic treatment non-response (NR) was defined as having documented treatment with at least two antipsychotics each above the minimum therapeutic dose as defined by the British National Formulary for > 4 weeks each, a CGI-SCH severity score of > 3, a PANSS total severity rating of at least 70, and a CRS adherence score of > 3. Fifty-two participants met criteria for antipsychotic non-response.

Materials

Clinical and demographic measures

Previous and existing drug use were measured using the Alcohol, Drug and Tobacco Inventory. Participants’ disorder severity was measured using the Mini-International Neuropsychiatric Interview (M-Psychotic Disorders; A-Major Depressive Episode; D-Manic/Hypomanic/Bipolar; MINI) [44], Structured Clinical Interview- Positive and Negative Syndrome Scale (SCI-PANSS) [45] and Clinical Global Impression-Schizophrenia scale (CGI-SCH) [42]. Concordance with medication was assessed using the Clinical Rating Scale for Schizophrenia (CRS) [40,41]. Participants also provided demographic data, such as years of previous full-time education, age, gender, as well as information regarding their previous antipsychotic history which were supplemented by medical records.

Measures of cognitive performance

Cognitive data was collected using the Brief Assessment of Cognition in Schizophrenia (BACS) [25] across all sites at the beginning of the assessment, following the administration of clinical and demographic measures. The battery is designed to take ~30 mins to complete, with minimal training demands, and is designed to be easily administered by clinical and healthcare workers [25]. The BACS (version A) [25] consists of six tests from the following cognitive domains: i) Verbal Memory: List learning task; ii) Working Memory:

For peer review only - http://bmjopen.bmj.com/site/about/guidelines.xhtml

Digit Sequencing task; iii) Motor Speed: Token motor task; iv) Verbal Fluency: Category instances task (Animals) and phonological (F and S-words); v) Attention and speed of information processing: Symbol Coding task; vi) Executive Functions: Tower of London task. All tasks on the BACS are scored with higher scores representing better performance. Composite z and t scores for the BACS are generated using normative data [46] and the following formulas: $Composite\ z\ score = \frac{\Sigma\left(\Sigma\frac{(raw\ score\ -\ normative\ score)}{normative\ standard\ deviation}\right)}{3.63}$ with each measure's z score summed and the total divided by 3.63; $Composite\ t\ score = (Composite\ z\ score * 10) +50$.

Data analysis

All analyses were conducted using STATA 15/SE [47]. Chi-square tests were used to compare cognitive performance across sites in case of site differences. Univariable regressions were used to compare cognitive performance between groups. Multivariable regression analyses were used to adjust univariable results for age, gender and illness duration, due to the reported relationship of age [48,49], gender [50,51] and illness duration [52,53] with cognitive outcomes. Analyses adjusting for anticholinergic effects of antipsychotic medication are presented in the supplementary material (Table S.1).

Results

Descriptive statistics of demographic and clinical variables between responder groups are reported in Table 1. In the antipsychotic responder group (N = 54), 4 were treated with a first-generation antipsychotic. For the non-responder group (N = 52), 5 were treated with a first-generation antipsychotic. All other participants were treated with second-generation antipsychotics.

For peer review only - http://bmjopen.bmj.com/site/about/guidelines.xhtml

Table 1

Demographic and clinical characteristics by group

	R			NR		
Demographic/clinical variable	N	Mean/ratio	SD	N	Mean/ratio	SD
Age	54	29.52	9.36	52	29.99	8.50
Gender (male : female)	54	46 : 8	-	52	43 : 9	-
Age of illness onset	53	26.10	6.53	50	25.31	5.93
Illness duration since 1st antipsychotic (years)	53	3.71	6.87	50	5.03	5.79
Duration from 1st psychotic symptom (years)	54	4.81	7.53	52	5.50	6.13
Duration from 1st contact with mental health services (years)	54	4.04	7.49	52	5.40	6.34
Full time education (years)	53	13.09	2.37	50	12.88	2.75
Chlorpromazine equivalents (mg/day)	53	305.45	146.86	52	343.73	202.83
PANSS positive score	54	12.24	3.40	42	22.65	3.54
PANSS negative score	54	13.82	3.38	52	20.96	4.56
PANSS total score	54	53.46	7.91	52	87.29	9.30
CGI positive symptoms score	53	3.26	.76	52	5.50	.10
CGI negative symptoms score	53	3.21	.86	52	4.88	1.04
CGI cognitive symptoms score	53	3.08	.83	52	4.83	1.22
CGI overall severity	53	3.42	.75	52	5.48	.58
Antipsychotic at assessment	54	Amisulpride = 3<br>Aripiprazole = 13<br>Clopixol = 2<br>Haloperidol = 1<br>Olanzapine = 19<br>Quetiapine = 4<br>Risperidone = 9<br>Flupentixol = 1<br>Paliperidone = 2	-	52	Amisulpride = 8<br>Aripiprazole = 10<br>Clopixol = 1<br>Haloperidol = 2<br>Olanzapine = 7<br>Quetiapine = 9<br>Risperidone = 6<br>Flupentixol = 1<br>Paliperidone = 6<br>Zuclopenthixol	-

For peer review only - http://bmjopen.bmj.com/site/about/guidelines.xhtml

acetate = 1

Note. R = antipsychotic responder; NR = antipsychotic non-responder; PANSS = Positive and Negative Syndrome Scale; CGI = Clinical Global Impression.

Cognitive performance

Mean scores for each group on all BACS tasks and standardized composite scores are displayed in Table 2. All measures of the BACS were normally distributed, with exception of the Tower of London task which was moderately negatively skewed (skewness = -0.95) as per the guidelines from Bulmer [54]. Cognitive performance on BACS composite and subtests did not significantly differ by site where data was collected.

For peer review only

For peer review only - http://bmjopen.bmj.com/site/about/guidelines.xhtml

Table 2

Mean group performance on BACS measures and univariable and multivariable linear regression models for response status and BACS performance

	R			NR			Unadjusted				Adjusted for age, gender and illness duration			
BACS measure	N	Mean	SD	N	Mean	SD	β	SE	95%CI	P-value	β	SE	95%CI	P-value
Verbal Memory	53	38.89	10.66	50	36.9	13.04	-1.99	2.34	-6.63 ; 2.66	.398	-2.68	2.38	-7.41 ; 2.05	.263
Digit Sequencing	53	17.87	4.95	50	17.98	4.09	0.11	0.90	-1.67 ; 1.89	.901	0.21	0.92	-1.61 ; 2.03	.818
Verbal Fluency	53	30.45	9.04	50	31.68	9.82	1.23	1.86	-2.46 ; 4.91	.510	1.12	1.92	-2.70 ; 4.92	.563
Token Motor	53	66.32	14.56	49	65.90	15.26	-0.42	2.95	-6.28 ; 5.43	.886	-1.05	2.93	-6.87 ; 4.78	.723
Symbol Coding	53	47.30	11.31	50	45.46	11.83	-1.84	2.28	-6.37 ; 2.68	.421	-1.71	2.35	-6.37 ; 2.95	.469
Tower of London	53	16.04	4.46	50	16.44	3.83	0.40	0.82	-1.23 ; 2.03	.625	0.50	0.83	-1.16 ; 2.15	.552
z score composite	53	-2.00	1.39	49	-2.03	1.51	-0.03	0.29	-0.60 ; 0.54	.922	-0.04	0.30	-0.63 ; 0.56	.908
t score composite	53	29.91	13.81	49	29.27	14.99	-0.64	2.87	-6.32 ; 5.05	.825	-0.75	2.99	-6.69 ; 5.19	.804

Note. R = antipsychotic responder; NR = antipsychotic non-responder; BACS = Brief Assessment of Cognition in Schizophrenia; CIs = confidence intervals.

For peer review only - http://bmjopen.bmj.com/site/about/guidelines.xhtml

Univariable linear regression analyses (Table 2) observed no significant relationships between response status and BACS performance. Multivariable models adjusted for age, gender and illness duration also observed no significant relationships between response status and cognitive outcomes (Table 2).

Discussion

The present investigation sought to compare specific cognitive deficits in antipsychotic responders (R) and antipsychotic non-responders (NR) using the Brief Assessment of Cognition in Schizophrenia (BACS) [25], anticipating the greatest deficits for NR in measures of verbal memory and verbal fluency when compared to R. Unlike previous cross-sectional studies [55-62], this investigation identified no significant differences in cognitive performance between groups.

Previous cross sectional research investigating differences in cognitive performance between antipsychotic treatment responders and treatment resistant cases have identified poorer performance in verbal, executive function, full-scale IQ cognitive measures [55,56,59-61], and verbal memory [55,58,60,62,63] in treatment resistant patients. A recent study using a similar methodology and sample size to ours also failed to show significant differences between antipsychotic responders and TRS cases on individual tasks of the BACS [64] but did observe significant differences on standardized (z and t) composite scores suggesting overall impairment in the TRS group. Our additional analyses also adjusting for anticholinergic effects (supplementary material: Table S.1) also observed no change to the relationship between BACS and antipsychotic response, suggesting no medication effects on our findings. We also further restricted our analysis to exclude participants that were under dosed (i.e. not within the 150-600mg/per day range) removing 12 participants (R = 5, NR = 7). No change was observed in the pattern of results.

The lack of significant differences in cognitive performance observed between R and NR groups in our study may be partly explained by the criteria used to define these groups. Unlike earlier investigations, our study did not include clozapine-treated patients, and there may have been less clinical separation between the R and NR groups than in some previous studies (as discussed in Egerton et al [39]). Furthermore, in our cross-sectional study design it is not possible to determine the proportion of participants in the NR group who would meet

For peer review only - http://bmjopen.bmj.com/site/about/guidelines.xhtml

criteria for TRS [65]. It is therefore possible the non-responder group was less severely unwell as in some previous studies, which may have reduced the ability to observe potential impairments in cognition due to clinical overlap. Previous investigations which observed group differences in cognitive performance between R and TRS included patients prescribed clozapine [56,57,59-61,63,64], and reported higher PANSS positive, negative and total scores[59,60,64], suggesting the NR/TRS groups may have had greater illness severity compared to our sample. Likewise, demographic and clinical variables previously found to be associated with antipsychotic response, such as a younger age and age of illness onset in non-responders [12-16], did not differ between treatment responders and non-responders in our sample, again suggesting group that compared to previous investigations, there wasn't enough clinical separation between our samples. In addition, the power calculations for sample size were generated on the basis of being able to provide > 95% power to detect differences in levels of anterior cingulate glutamate [39] (see Protocol provided in supplementary material) and it is possible that the sample was underpowered to detect neurocognitive differences using the BACS.

It is also possible that our definition of antipsychotic response and inclusion criteria may have influenced our findings. As per definition, differences were only observed between groups on CGI-SCH and PANSS measures of symptom severity. Psychotic symptoms such as hallucinations, delusions and paranoia (i.e. schizophrenia-like symptoms) have been attributed to D2 dopamine receptors and functioning in the striatum, as evidenced by animal models [65]. It has also been reported that following amphetamine administration, hyperactivity of dopamine transition is associated with the activation of psychotic symptoms. However, amphetamine induced psychosis does not tend to exhibit negative and cognitive symptoms [66]. In contrast, cognitive deficits in schizophrenia have been reported to be related to functioning in the dorsolateral prefrontal cortex (DLPFC) [67,68], glutamate to GABA ratios in the DLPFC [69], as well as prefrontal glutamate levels in the dorsal anterior cingulate cortex in antipsychotic-naïve patients [69]. Unlike psychotic symptoms, the Dopamine D1 receptor signalling is essential for cognition [70]. Therefore, it is possible that the differences in the neurobiological underpinnings between psychotic and cognitive symptoms may also explain why no cognitive differences were observed between groups, as this was biased in favour of psychotic symptoms due to our inclusion criteria.

For peer review only - http://bmjopen.bmj.com/site/about/guidelines.xhtml

Another consideration is that our study focused on younger patients early in their treatment trajectories to reduce the potential effects of chronicity and previous medication, with a mean length of treatment of 3 to 4 years. Most previous cross-sectional investigations also include older samples with a longer duration of illness [56,57,59,60,64], although differences in measures of verbal intelligence and fluency have been quantifiable at the first episode in treatment resistant psychosis[27]. Trajectory modelling of cognitive performance in FEP has observed deficits in executive function performance, relative to controls, with these remaining stable over illness duration [71]. However, deficits in verbal knowledge and memory became more apparent and exaggerated relative to controls following the first episode [72]. Similar exaggerated declines following the first episode have also been observed in measures of verbal memory [71,73]. With our sample of patients being early in their treatment, cognitive deficits may have been less marked at this illness stage.

Despite not detecting significant differences between antipsychotic responder groups, it is worth mentioning the importance of conducting research using clinically transferable measures of cognitive impairment. It may be possible for future researchers to use machines learning algorithms to identify subgroups of schizophrenia from cognitive outcomes. Bak et al [74] used Gaussian mixture modelling to identify two distinct subgroups in antipsychotic-naive first episode schizophrenia samples. In this study, cognitive and electrophysiological data were used to identify the two groups. When predicting treatment response, assessed by the PANSS, there was a significant predictive relationship between group and antipsychotic response. Therefore, future research should aim to use more machine learning techniques to identify patterns of cognitive performance within schizophrenia subsamples and investigate antipsychotic response between these groups.

Conclusions

Within this cross-sectional investigation we observed no differences in cognitive performance between antipsychotic responders and non-responders. This may be because there was less clinical separation between these groups in our sample in comparison to previous investigations. Future investigations should consider the role of machine learning techniques to investigate the role of cognitive functions in identifying subgroups of

For peer review only - http://bmjopen.bmj.com/site/about/guidelines.xhtml

schizophrenia using first episode cohorts and how this may differ in future stages of treatment resistance. Such research using antipsychotic-naïve patients versus healthy controls has observed strong group discrimination using cognitive measures in comparison to electrophysiology and magnetic resonance imaging methods [75], with other investigations observing distinct subgroups in schizophrenia from differences in early information processing and higher cognitive functions [74].

Funding

STRATA is funded by a grant from the Medical Research Council (MRC) to JHM, grant reference MR/L011794. E.M.'s PhD is funded by the MRC-doctoral training partnership studentship in Biomedical Sciences at King's College London. J.H.M., E.K., R.M.M., A.E., O.D.H. are part funded by the National Institute for Health Research (NIHR) Biomedical Research Centre at South London and Maudsley NHS Foundation Trust and King's College London. In the past three years, S.M.L. has received personal support from Sunovion. The views expressed are those of the authors and not necessarily those of the NHS, the MRC, the NIHR, Sunovion or the Department of Health.

Competing interest

The remaining authors report no conflicts of interest.

Acknowledgments

For more information on STRATA please visit; https://gtr.ukri.org/project/7F7F0378-8FD7-4A33-91AD-277A49EF4908). STRATA was funded by a grant from the Medical Research Council (MRC), to Prof James MacCabe. Data were collected between March 2015 and February 2017.

Data Availability

At the time of submission, the data governance frameworks are being put in place to make a fully anonymized version of the data available to the wider research community via the TranSMART data sharing platform: https://transmartfoundation.org/. To apply for access to the data, please contact J.H.M. at james.maccabe@kcl.ac.uk.

For peer review only - http://bmjopen.bmj.com/site/about/guidelines.xhtml

Author contributions

J.H.M., R.M.M., O.D.H., A.E., E.K., R.D., L.K., J.D., A.M., T.C., J.L., C.S.P., J.W., J.W. and S.M.L. contributed to the design and implementation of the study. E.M. completed analyses and wrote the manuscript with the assistance of J.H.M. and E.K.. J.H.M., R.M.M., O.D.H., A.E., E.K., S.M.L., J.D., S.L., provided comments on the manuscript.

For peer review only

For peer review only - http://bmjopen.bmj.com/site/about/guidelines.xhtml

References

1 National Institute for Health and Care Excellence, National Collaborating Centre for Mental Health (UK. Psychosis and schizophrenia in adults: treatment and management). 2014.

2 Elkis H. Treatment-resistant schizophrenia. *Psychiatric Clinics of North America*. 2007 Sep1;30(3):511-33.

3 Demjaha A, Lappin JM, Stahl D, et al. Antipsychotic treatment resistance in first-episode psychosis: prevalence, subtypes and predictors. *Psychological medicine*. 2017 Aug;47(11):1981-9.

4 Kane JM, Agid O, Baldwin ML, et al. Clinical guidance on the identification and management of treatment-resistant schizophrenia. *The Journal of clinical psychiatry*. 2019 Mar5;80(2):0-.

5 Üçok A, Çıkrıkçılı U, Ergül C, et al. Correlates of clozapine use after a first episode of schizophrenia: results from a long-term prospective study. *CNS drugs*. 2016 Oct;30(10):997-1006.

6 Köhler-Forsberg O, Horsdal HT, Legge SE, et al. Predictors of nonhospitalization and functional response in clozapine treatment: a nationwide, population-based cohort study. *Journal of clinical psychopharmacology*. 2017 Apr1;37(2):148-54.

7 Yoshimura B, Yada Y, So R, et al. The critical treatment window of clozapine in treatment-resistant schizophrenia: secondary analysis of an observational study. *Psychiatry research*. 2017 Apr1;250:65-70.

8 Griffiths K, Millgate E, Egerton A, et al. Demographic and clinical variables associated with response to clozapine in schizophrenia: a systematic review and meta-analysis. *Psychological Medicine*. 2021 Feb1:1-1.

9 Lally J, Gaughran F, Timms P, et al. Treatment-resistant schizophrenia: current insights on the pharmacogenomics of antipsychotics. *Pharmacogenomics and personalized medicine*. 2016;9:117.

10 Gillespie AL, Samanaite R, Mill J, et al. Is treatment-resistant schizophrenia categorically distinct from treatment-responsive schizophrenia? A systematic review. *BMC psychiatry*. 2017 Dec;17(1):1-4.

For peer review only - http://bmjopen.bmj.com/site/about/guidelines.xhtml

11 Lally J, Ajnakina O, Di Forti M, et al. Two distinct patterns of treatment resistance: clinical predictors of treatment resistance in first-episode schizophrenia spectrum psychoses. *Psychological medicine*. 2016 Nov;46(15):3231-40.

12 Wimberley T, Støvring H, Sørensen HJ, et al. Predictors of treatment resistance in patients with schizophrenia: a population-based cohort study. *The Lancet Psychiatry*. 2016 Apr1;3(4):358-66.

13 Smart SE, Kępińska AP, Murray RM et al. Predictors of treatment resistant schizophrenia: a systematic review of prospective observational studies. *Psychological medicine*. 2021 Jan;51(1):44-53.

14 Legge S, Dennison C, Pardinas A, et al. Clinical indicators of treatment-resistant psychosis. *British Journal of Psychiatry*. 2020 May31;216(5):259-66.

15 Bozzatello P, Bellino S, Rocca P. Predictive factors of treatment resistance in first episode of psychosis: a systematic review. *Frontiers in psychiatry*. 2019 Feb26;10:67.

16 Smart S. *Clinical and Genetic Predictors of Treatment Resistant Psychosis* (Doctoral dissertation, King's College London). 2020.

17 Toulopoulouand T, Murray RM. Verbal memory deficit in patients with schizophrenia: an important future target for treatment. *Expert review of neurotherapeutics*. 2004 Jan1;4(1):43-52.

18 Fatouros-Bergman H, Cervenka S, Flyckt L, et al. Meta-analysis of cognitive performance in drug-naïve patients with schizophrenia. *Schizophrenia research*. 2014 Sep1;158(1-3):156-62.

19 Mesholam-Gately RI, Giuliano AJ, Goff KP, et al. Neurocognition in first-episode schizophrenia: a meta-analytic review. *Neuropsychology*. 2009 May;23(3):315.

20 Kravariti E, Morgan K, Fearon P, et al. Neuropsychological functioning in first-episode schizophrenia. *The British Journal of Psychiatry*. 2009 Oct;195(4):336-45.

21 Snitz BE, MacDonald III AW, Carter CS. Cognitive deficits in unaffected first-degree relatives of schizophrenia patients: a meta-analytic review of putative endophenotypes.

22 Bora E, Özerdem A. Meta-analysis of longitudinal studies of cognition in bipolar disorder: comparison with healthy controls and schizophrenia. *Psychological medicine*. 2017 Dec;47(16):2753-66.

23 Fagerlund B, Pantelis C, Jepsen JR, Raghava JM, Rostrup E, Thomas MB, Nielsen MØ, Bojesen K, Jensen KG, Stentebjerg-Decara M, Klauber DG. Differential effects of age at illness onset on verbal memory functions in antipsychotic-naïve schizophrenia patients aged 12–43 years. *Psychological medicine*. 2020 Mar 11:1-1.

For peer review only - http://bmjopen.bmj.com/site/about/guidelines.xhtml

24 Millgate E, Hide O, Lawrie S, et al. Neuropsychological differences between treatment-resistant and treatment-responsive schizophrenia: a systematic review & meta-analysis. [Manuscript submitted for publication]. Institute of Psychiatry, Psychology and Neuroscience, King's College London

25 Keefe RS, Goldberg TE, Harvey PD, et al. The Brief Assessment of Cognition in Schizophrenia: reliability, sensitivity, and comparison with a standard neurocognitive battery. *Schizophrenia research*. 2004 Jun1;68(2-3):283-97.

26 Velligan DI, Bow-Thomas CC. Executive function in schizophrenia. InSeminars in clinical neuropsychiatry 1999 Jan1(Vol. 4, No. 1, pp. 24-33).

27 Kravariti E, Dixon T, Frith C, et al. Association of symptoms and executive function in schizophrenia and bipolar disorder. *Schizophrenia research*. 2005 May1;74(2-3):221-31.

28 Lee J, Park S. Working memory impairments in schizophrenia: a meta-analysis. *Journal of abnormal psychology*. 2005 Nov;114(4):599.

29 Forbes NF, Carrick LA, McIntosh AM, et al. Working memory in schizophrenia: a meta-analysis. *Psychological medicine*. 2009 Jun1;39(6):889.

30 Flashman LA, Flaum M, Gupta S, et al. Soft signs and neuropsychological performance in schizophrenia. *The American journal of psychiatry*. 1996 Apr.

31 Bruder GE, Wexler BE, Sage MM, et al. Verbal memory in schizophrenia: additional evidence of subtypes having different cognitive deficits. *Schizophrenia Research*. 2004 Jun1;68(2-3):137-47.

32 Hill SK, Beers SR, Kmiec JA, et al. Impairment of verbal memory and learning in antipsychotic-naıve patients with first-episode schizophrenia. *Schizophrenia research*. 2004 Jun1;68(2-3):127-36.

33 Bokat CE, Goldberg TE. Letter and category fluency in schizophrenic patients: a meta-analysis. *Schizophrenia research*. 2003 Nov1;64(1):73-8.

34 Ojeda N, Sánchez P, Peña J, et al. Verbal fluency in schizophrenia: does cognitive performance reflect the same underlying mechanisms in patients and healthy controls?. *The Journal of nervous and mental disease*. 2010 Apr1;198(4):286-91.

35 Harris JG, Minassian A, Perry W. Stability of attention deficits in schizophrenia. *Schizophrenia research*. 2007 Mar1;91(1-3):107-11.

For peer review only - http://bmjopen.bmj.com/site/about/guidelines.xhtml

36 Carter JD, Bizzell J, Kim C, et al. Attention deficits in schizophrenia—preliminary evidence of dissociable transient and sustained deficits. *Schizophrenia research*. 2010 Sep1;122(1-3):104-12.

37 Rose D, Papoulias C, MacCabe J, Walke J. Service users' and carers' views on research towards stratified medicine in psychiatry: a qualitative study. *BMC research notes.* 2015 Dec;8(1):1-9.

38 American Psychiatric Association. Diagnostic and statistical manual of mental disorders (DSM-5®). American Psychiatric Pub; 2013 May 22.

39 Egerton A, Murphy A, Donocik J, et al. Dopamine and Glutamate in Antipsychotic-Responsive Compared With Antipsychotic-Nonresponsive Psychosis: A Multicenter Positron Emission Tomography and Magnetic Resonance Spectroscopy Study (STRATA). *Schizophrenia Bulletin*. 2020 Sep10.

40 Kemp R, Hayward P, Applewhaite G, et al. Compliance therapy in psychotic patients: randomised controlled trial. *Bmj.* 1996 Feb10;312(7027):345-9.

41 Kemp R, Kirov G, Everitt B, et al. Randomised controlled trial of compliance therapy. *The British Journal of Psychiatry*. 1998 May1;172(5):413-9.

42 Guy W. ECDEU assessment manual for psychopharmacology. US Department of Health, Education, and Welfare, Public Health Service, Alcohol, Drug Abuse, and Mental Health Administration, National Institute of Mental Health, Psychopharmacology Research Branch, Division of Extramural Research Programs; 1976.

43 Kay SR, Fiszbein A, Opler LA. The positive and negative syndrome scale (PANSS) for schizophrenia. *Schizophrenia bulletin.* 1987;13(2):261.

44 Sheehan DV, Lecrubier Y, Sheehan KH, et al. The Mini-International Neuropsychiatric Interview (MINI): the development and validation of a structured diagnostic psychiatric interview for DSM-IV and ICD-10. *Journal of clinical psychiatry*. 1998 Jan1;59(20):22-33.

45 Opler LA, Kay SR, Lindenmayer JP, et al. Structured clinical interview: The positive and negative syndrome scale (SCI-PANSS). North Tonawanda, NY: Multi-Health Systems. 1999.

46 Keefe RS, Harvey PD, Goldberg TE, et al. Norms and standardization of the Brief Assessment of Cognition in Schizophrenia (BACS). *Schizophrenia research.* 2008 Jul1;102(1-3):108-15.

47 StataCorp LL. Stata Statistical Software: Release 15 College Station, TX, 2017. www. stata. com/features/documentation/(last accessed on 1 March 2018). 2017.

For peer review only - http://bmjopen.bmj.com/site/about/guidelines.xhtml

48 Craik FI, Bialystok E. Cognition through the lifespan: mechanisms of change. *Trends in cognitive sciences*. 2006 Mar1;10(3):131-8.

49 Harvey PD. What is the evidence for changes in cognition and functioning over the lifespan in patients with schizophrenia?. *The Journal of clinical psychiatry.* 2014 Jun5;75(suppl 2):34-8.

50 Perlick D, Mattis S, Stastny P, et al. Gender differences in cognition in schizophrenia. *Schizophrenia Research.* 1992 Oct1;8(1):69-73.

51 Han M, Huang XF, Xiu MH, et al. Gender differences in cognitive function of patients with chronic schizophrenia. *Progress in neuro-psychopharmacology and biological psychiatry*. 2012 Dec3;39(2):358-63.

52 Cuesta MJ, Peralta V, Zarzuela A. Illness duration and neuropsychological impairments in schizophrenia. *Schizophrenia research*. 1998 Oct.

53 Bagney A, Rodriguez-Jimenez R, Martinez-Gras I, Sanchez-Morla EM, Santos JL, Jimenez-Arriero MA, Lobo A, McGorry PD, Palomo T. Negative symptoms and executive function in schizophrenia: does their relationship change with illness duration?. *Psychopathology*. 2013;46(4):241-8.

54 Bulmer MG. Principles of statistics. Courier Corporation; 1979.

55 Lawrie SM, Ingle GT, Santosh CG, et al. Magnetic resonance imaging and single photon emission tomography in treatment-responsive and treatment-resistant schizophrenia. *The British Journal of Psychiatry.* 1995 Aug1;167(2):202-10.

56 Smith RC, Kadewari RP, Rosenberger JR, et al. Nonresponding schizophrenia: differentiation by neurological soft signs and neuropsychological tests. *Schizophrenia bulletin.* 1999 Jan1;25(4):813-25.

57 Joober R, Rouleau GA, Lal S, et al. Neuropsychological impairments in neuroleptic-responder vs.-nonresponder schizophrenic patients and healthy volunteers. *Schizophrenia Research.* 2002 Jan15;53(3):229-38.

58 de Bartolomeis A, Balletta R, Giordano S, et al. Differential cognitive performances between schizophrenic responders and non-responders to antipsychotics: correlation with course of the illness, psychopathology, attitude to the treatment and antipsychotics doses. *Psychiatry research.* 2013 Dec15;210(2):387-95.

59 Bourque J, Lakis N, Champagne J, et al. Clozapine and visuospatial processing in treatment-resistant schizophrenia. *Cognitive Neuropsychiatry.* 2013 Nov1;18(6):615-30.

For peer review only - http://bmjopen.bmj.com/site/about/guidelines.xhtml

60 Frydecka D, Beszłej JA, Gościmski P, et al. Profiling cognitive impairment in treatment-resistant schizophrenia patients. *Psychiatry research.* 2016 Jan30;235:133-8.

61 Kravariti E, Demjaha A, Zanelli J, et al. Neuropsychological function at first episode in treatment-resistant psychosis: findings from the ÆSOP-10 study. *Psychological medicine.* 2019 Sep;49(12):2100-10.

62 Huang J, Zhu Y, Fan F, et al. Hippocampus and cognitive domain deficits in treatment-resistant schizophrenia: A comparison with matched treatment-responsive patients and healthy controls☆,☆☆,★,★★. *Psychiatry Research: Neuroimaging.* 2020 Mar30;297:111043.

63 Iasevoli F, Avagliano C, Altavilla B, et al. Evaluation of a few discrete clinical markers may predict categorization of actively symptomatic non-acute schizophrenia patients as treatment resistant or responders: a study by ROC curve analysis and multivariate analyses. *Psychiatry research.* 2018 Nov1;269:481-93.

64 Sosin D, Ivashchenko D, Sozaeva Z, et al. Cognitive impairment in patients with treatment resistant schizophrenia: Associations with DRD2, DRD3, HTR2A, BDNF and CYP2D6 genetic polymorphisms. *Neurology, Psychiatry and Brain Research.* 2019 Sep1;33:48-55.

65 Kellendonk C, Simpson EH, Polan HJ, Malleret G, Vronskaya S, Winiger V, Moore H, Kandel ER. Transient and selective overexpression of dopamine D2 receptors in the striatum causes persistent abnormalities in prefrontal cortex functioning. *Neuron*. 2006 Feb 16;49(4):603-15.

66 Voce A, Calabria B, Burns R, Castle D, McKetin R. A systematic review of the symptom profile and course of methamphetamine-associated psychosis: substance use and misuse. *Substance use & misuse*. 2019 Mar 21;54(4):549-59.

67 Miller EK, Cohen JD. An integrative theory of prefrontal cortex function. *Annual review of neuroscience*. 2001 Mar;24(1):167-202.

68. Schoonover KE, Dienel SJ, Lewis DA. Prefrontal cortical alterations of glutamate and GABA neurotransmission in schizophrenia: Insights for rational biomarker development. *Biomarkers in Neuropsychiatry*. 2020 Dec 1;3:100015.

69 Bojesen KB, Broberg BV, Fagerlund B, Jessen K, Thomas MB, Sigvard A, Tangmose K, Nielsen MØ, Andersen GS, Larsson HB, Edden RA. Associations between cognitive function and levels of glutamatergic metabolites and gamma-aminobutyric acid in antipsychotic-naïve patients with schizophrenia or psychosis. *Biological psychiatry*. 2021 Feb 1;89(3):278-87.

For peer review only - http://bmjopen.bmj.com/site/about/guidelines.xhtml

70 Arnsten AF. Catecholamine modulation of prefrontal cortical cognitive function. *Trends in cognitive sciences*. 1998 Nov 1;2(11):436-47.

71 Chan SK, Chan HY, Honer WG, et al. Predictors of Treatment-Resistant and Clozapine-Resistant Schizophrenia: A 12-Year Follow-up Study of First-Episode Schizophrenia-Spectrum Disorders. *Schizophrenia Bulletin.* 2020 Oct12.

72 Zanelli J, Mollon J, Sandin S, et al. Cognitive change in schizophrenia and other psychoses in the decade following the first episode. *American Journal of Psychiatry.* 2019 Oct1;176(10):811-9.

73 Barder HE, Sundet K, Rund B, et al. Ten year neurocognitive trajectories in first-episode psychosis. *Frontiers in human neuroscience.* 2013 Oct7;7:643.

74 Bak N, Ebdrup BH, Oranje B, Fagerlund B, Jensen MH, Düring SW, Nielsen MØ, Glenthøj BY, Hansen LK. Two subgroups of antipsychotic-naive, first-episode schizophrenia patients identified with a Gaussian mixture model on cognition and electrophysiology. *Translational psychiatry*. 2017 Apr;7(4):e1087-.

75 Ebdrup BH, Axelsen MC, Bak N, Fagerlund B, Oranje B, Raghava JM, Nielsen MØ, Rostrup E, Hansen LK, Glenthøj BY. Accuracy of diagnostic classification algorithms using cognitive-, electrophysiological-, and neuroanatomical data in antipsychotic-naïve schizophrenia patients. *Psychological medicine*. 2019 Dec;49(16):2754-63.

For peer review only

For peer review only - http://bmjopen.bmj.com/site/about/guidelines.xhtml

Supplementary material
Table S.1
Univariable and multivariable linear regression models for response status and BACS performance

	R	NR	Unadjusted				Adjusted for age, gender, illness duration and anticholinergic effects				Adjusted for anticholinergic effects			
BACS measure	N	N	β	SE	95%CI	P-value	β	SE	95%CI	P-value	β	SE	95%CI	P-value
Verbal Memory	53	50	-1.99	2.34	-6.63 ; 2.66	.398	-3.18	2.38	-7.90 ; 1.54	.185	-2.34	2.35	-7.00 ; 2.32	.322
Digit Sequencing	53	50	0.11	0.90	-1.67 ; 1.89	.901	0.07	0.92	-1.76 ; 1.89	.944	-0.02	0.90	-1.81 ; 1.77	.983
Verbal Fluency	53	50	1.23	1.86	-2.46 ; 4.91	.510	1.08	1.94	-2.78 ; 4.94	.580	1.17	1.88	-2.56 ; 4.90	.536
Token Motor	53	49	-0.42	2.95	-6.28 ; 5.43	.886	-1.40	2.97	-7.29 ; 4.50	.638	-0.62	2.99	-6.56 ; 5.31	.835
Symbol Coding	53	50	-1.84	2.28	-6.37 ; 2.68	.421	-1.89	2.37	-6.60 ; 2.83	.428	-2.04	2.30	-6.60 ; 2.53	.378
Tower of London	53	50	0.40	0.82	-1.23 ; 2.03	.625	0.35	0.84	-1.30 ; 2.01	.672	0.23	0.82	-1.40 ; 1.85	.782
z score composite	53	49	-0.03	0.29	-0.60 ; 0.54	.922	-0.08	0.30	-0.68 ; 0.52	.798	-0.07	0.29	-0.65 ; 0.50	.800
t score composite	53	49	-0.64	2.87	-6.32 ; 5.05	.825	-1.33	3.02	-7.32 ; 4.67	.662	-1.24	2.88	-6.96 ; 4.48	.668

Note. R = antipsychotic responder; NR = antipsychotic non-responder; BACS = Brief Assessment of Cognition in Schizophrenia; CIs = confidence intervals.

For peer review only

STRATA: Investigating factors associated with response to antipsychotic treatment
Protocol. Version 4.0, 19th August 2016
REC REF: 15/LO/0038

STRATA: Investigating factors associated with response to antipsychotic treatment

Sponsor

Name of organisation: King's College London
Address: Room 1.8 Hodgkin Building, Guy's Campus, London SE1 4UL
Telephone: 020 7848 6960
Fax:
Email: keith.brennan@kcl.ac.uk

Chief Investigator

Name: Dr James MacCabe
Address: Institute of Psychiatry, Psychology & Neuroscience (IoPPN), De Crespigny Park, SE5 8AF
Telephone: 02078480757
Fax:
Email: james.maccabe@kcl.ac.uk

Name and address of Principal Investigator (on grant award).

Name: Prof Shitij Kapur
Address: Institute of Psychiatry, Psychology & Neuroscience (IoPPN), De Crespigny Park, SE5 8AF
Telephone: 020 7848 0593
Fax:
Email: shitij.kapur@kcl.ac.uk

Name and address of lead Co-Investigators at sites

Name: Prof Shon Lewis
Address: The University of Manchester, University Place, Oxford Road, Manchester, M13 9PL
Telephone: 0161 306 7944
Fax:
Email: shon.lewis@manchester.ac.uk

Name: Prof Mick O'Donovan
Address: MRC Centre for Neuropsychiatric Genetics and Genomics, Cardiff University, Henry Wellcome Building, Heath Park, Cardiff, CF14 4XN
Telephone: (029) 20687066
Fax:
Email: odonovanmc@cardiff.ac.uk

For peer review only

For peer review only - http://bmjopen.bmj.com/site/about/guidelines.xhtml

STRATA: Investigating factors associated with response to antipsychotic treatment
Protocol. Version 4.0, 19th August 2016
REC REF: 15/LO/0038

Name: Prof Stephen Lawrie
Address: University of Edinburgh, Royal Edinburgh Hospital, Edinburgh, EH10 5HF
Telephone: 0131 537 6671
Fax:
Email: s.lawrie@ed.ac.uk

For peer review only

For peer review only - http://bmjopen.bmj.com/site/about/guidelines.xhtml

STRATA: Investigating factors associated with response to antipsychotic treatment
Protocol. Version 4.0, 19th August 2016
REC REF: 15/LO/0038

CONTENTS

1. Introduction

2. Study Objectives and Design

2.1. Study Objectives

2.2 Study Design

3. Sample Size, Statistics, Selection and Withdrawal of Subjects

4. Study procedures

5. Sample Handling and Laboratories

6. Assessment of Safety

7. Study oversight arrangements

8. Ethics and Regulatory Approvals

9. Data Handling

10. Finance & Publication Policy

For peer review only - http://bmjopen.bmj.com/site/about/guidelines.xhtml

STRATA: Investigating factors associated with response to antipsychotic treatment
Protocol. Version 4.0, 19th August 2016
REC REF: 15/LO/0038

Study Synopsis

Full Title	Investigating factors associated with response to antipsychotic treatment
Short Title/Acronym	STRATA
Protocol Version number and Date	Version 4.0, 19th August 2016
Study Duration	36 months
Study Design	Basic Science
Sponsor/Co-sponsors	Kings College London / South London and Maudsley NHS Foundation Trust
Chief Investigator	Dr James MacCabe
REC number	15/LO/0038
Primary objective	The principle objective is to use Positron Emission Tomography (PET), Magnetic Resonance Spectroscopy (MRS), genetic and clinical data to confirm recent evidence of a distinct subtype of schizophrenia, based on differences in dopamine and glutamate function which would lead to developing a clinically useful, acceptable and cost-effective stratification tool.
Secondary objective (s)	To establish a lasting network of academia and industry partners and patients databases to facilitate and expedite both follow-up and novel research built to address patient stratification.<br><br>To examine and improve test-re-test and intercentre reliability of neuroimaging procedures for future large scale multisite studies in this mould that will be conducted.
Number of Subject	100
Main Inclusion Criteria	aged 18-65;<br><br>DSM 5 schizophrenia/schizophreniform disorder.

For peer review only - http://bmjopen.bmj.com/site/about/guidelines.xhtml

STRATA: Investigating factors associated with response to antipsychotic treatment
Protocol. Version 4.0, 19th August 2016
REC REF: 15/LO/0038

Statistical Methodology and Analysis	Summary statistics will be used to describe the demographic and clinical characteristics of each participant group.<br><br>Group differences in demographic, clinical variables and 18F-DOPAKi and glutamate concentration will be determined using pre-specified between group comparisons as appropriate (e.g. Chi square; Fischer's Exact; ANOVA).

For peer review only

For peer review only - http://bmjopen.bmj.com/site/about/guidelines.xhtml

STRATA: Investigating factors associated with response to antipsychotic treatment
Protocol. Version 4.0, 19th August 2016
REC REF: 15/LO/0038

1. Introduction

People with schizophrenia suffer from a range of symptoms including hallucinations (such as hearing voices), delusions (false beliefs) and thought disorder (thoughts not flowing in a logical way), as well as 'negative symptoms' such as a lack of motivation and withdrawal from social contact. Currently, antipsychotic medication is the mainstay of treatment for schizophrenia and all existing antipsychotic medications are thought to work by acting to reduce transmission of a brain chemical called dopamine. However, even after attempts to treat the disorder with two different antipsychotics, around 30% of patients still fail to improve. When this happens, the medical guidelines recommend treatment with a different drug called clozapine. However clozapine has several side effects and requires regular blood tests, so people do not like taking it. It is also ineffective in some patients.

The result is that a large number of patients spend too long on ineffective drugs which impact greatly on their mental health, well-being and quality of life whilst the cost of ineffective treatment is a huge financial burden to the NHS, consuming 25-50% of the total national mental health budget.

STRATA (funded by a £5M Medical Research Council award) aims to build on new evidence from neuroimaging and genetics studies suggesting that those who do not respond may actually have a completely different neurochemical abnormality causing their symptoms (the same sort of symptoms as are caused by excessive dopamine), involving a different chemical called glutamate. There are some new medicines under development that we hope will help people whose illness has not responded to standard medicines acting on dopamine.

We aim to develop a method to predict, even as early as when first seen, which patients will respond to standard dopamine drugs, and which people are instead more likely to respond to the new glutamate drugs. This will allow people to receive the medicines they need straight away, without having to try ineffective drugs first.

The proposed research programme is broken down into several parts. This protocol describes the first study, which is a UK, multicentre study using brain scans to confirm that those patients who don't respond to standard treatments have higher glutamate levels, but lower dopamine levels than those who respond well. This information, along with clinical and genetic information, will be used to develop tests to identify in advance which people will respond to dopaminergic versus glutamatergic medication.

2. Study Objectives and Design

2.1. Study Objectives and Outcomes

The principle objective is to use Positron Emission Tomography (PET), Magnetic Resonance Spectroscopy (MRS), genetic and clinical data to confirm recent evidence of a distinct subtype of schizophrenia, based on differences in dopamine and glutamate function which would lead to developing a clinically useful, acceptable and cost-effective stratification tool.

The secondary research objectives are:

For peer review only - http://bmjopen.bmj.com/site/about/guidelines.xhtml

i) To establish a lasting network of academia and industry partners and patient databases to facilitate and expedite both follow-up and novel research built to address patient stratification.

ii) To examine and improve test-re-test and intercentre reliability of neuroimaging procedures for future large scale multisite studies in this mould that will be conducted.

The study is designed to generate a predictive test for treatment response so the outcome will be the overall measure generated. The data that will lead to this will include MRS glutamate level, the PET Ki value, polygenic risk score and clinical variables such as PANSS score.

2.2 Study Design

STRATA is a multi centred study. 100 participants will be recruited across 4 university research sites including KCL, University of Manchester, Cardiff University, and University of Edinburgh.

Participants will consent to all aspects of the study including interviews/assessments, blood and urine sampling, MRI scan and PET scan (the latter in London and Manchester only) but can also choose to opt out of some tasks if necessary.

1. Assessments

An initial interview will collect demographic and personal information (e.g. address, contact details, date of birth, gender, handedness, head injury and other relevant medical history), and structured assessments of medication history and response. Clinical information will also be recorded from medical records. The Mini International Neuropsychiatric Interview (MINI) will be used to confirm diagnosis, which takes around 15 minutes to complete.

Illness severity will be measured using:

i. Positive and Negative Syndrome Scale (PANSS),
ii. Clinical Global Impression scale (CGI -SCH)
iii. Kemp Clinician Rating Scale (of adherence to treatment)
iv. Brief Assessment of Cognition in Schizophrenia (BACS)

2. Biological samples

Blood samples will be collected via cannula (as described under the PET scan section below) or by venous puncture, during a routine blood sample whenever possible. The participant will give up to 50ml in blood (around 3 tablespoons), this is in line with sampling guidelines.

While the biological sample collection is ongoing, samples will be stored at the laboratory corresponding to each research site. The samples for genetics analysis will subsequently be transferred to the MRC Center for Neuropsychiatric Genetics and Genomics, Cardiff University.

KCL/South London and the Maudsley (SLaM) participants will also be invited to participate in the BRC Biobank. This is covered by a separate ethical approval (09/H0606/84 NRES Committee South Central-Oxford C.)

For peer review only

For peer review only - http://bmjopen.bmj.com/site/about/guidelines.xhtml

STRATA: Investigating factors associated with response to antipsychotic treatment
Protocol. Version 4.0, 19th August 2016
REC REF: 15/LO/0038

Participants will be asked to provide samples (urine and blood) for metabolomics analysis. This will be processed at MRC-NIHR National Phenome Centre. The Centre is funded by the MRC and NIHR and led by Imperial College London and King's College London.

As of April 2016, participants will also have a sample taken for proteomic analysis. These samples will be sent to the University of Manchester (Molecular Pathology Innovation Centre). This will be within the 50ml sampling guideline already approved.

3. Magnetic Resonance Imaging (MRI)

The MRI scans (100 in total) will take place at four locations (London, Cardiff, Manchester and Edinburgh) at NHS Trust or University sites. The MRI session will last a maximum of 1 hour. During the scan, participants will be asked to lie flat on their back with their head inside the scanner. The scanner makes a loud noise as it takes pictures, so participants will be given headphones to wear and asked to lie as still as possible. The researcher will be able to speak to the participant over the microphone throughout and participants will be told if they do feel uncomfortable the session can be stopped at any time. The MRI scan itself is painless and safe. Some people find scans claustrophobic or anxiety-provoking, and we have a mock scanner that participants can try out first. The scanner consists of a powerful magnet, which may attract metal objects. Therefore before the scan participants will go through a safety questionnaire, to check that they can have the scan. If a participant has any metal in their body, either from accidents or operations, they may not be able to have the MRI scan, but they can still take part in the rest of the study.

All data collection will occur at 3 Tesla. During the scan, data acquisition will include acquisition of localizer, T1-weighted and T2-weighted structural scans. 1H-MRS data for measurement of regional concentrations of glutamate and other metabolites present in the 1H-MRS spectra will be acquired using conventional PRESS (Point RESolved Spectrocopy) acquisition routines, as well as a resting state fMRI sequence if time allows.

Due to change of scanner at Cardiff University, participants recruited in Cardiff prior to the decommissioning of the old scanner will be re-contacted and asked whether they would volunteer for a second MRI scan on the new scanner. They will also be asked to repeat some of the interview/assessments and may be asked for biological samples (only in circumstances where these were not provided previously). Participants will be reimbursed for their time at the same rate.

In the unlikely event that MRI scanner issues or excessive movement make the MRS data unusable at other sites, participants can be re-contacted and asked whether they would like to volunteer for a second scan.

4. Positron Emission Tomography (PET)

The PET scans (60 in total, subset of those having MRI scans) will take place at two sites:
i) Imanova Limited, Imperial College London, Hammersmith Hospital in London.
ii)The Wolfson Molecular Imaging Centre in Manchester.

PET with the radiotracer 18F-DOPA will be used to assess brain dopaminergic function in a sub-set of participants (N=60) recruited at KCL and University of Manchester. The PET scan procedure involves an initial transmission scan followed by a dynamic scan lasting approximately

For peer review only - http://bmjopen.bmj.com/site/about/guidelines.xhtml

90 minutes after injection of the radiotracer 18F DOPA through a cannula inserted into an arm vein. In the event the participant has to get off the scanner e.g. to go to the toilet or for some other reason then the transmission scan may be repeated to reposition them in the camera. In the unlikely event of technical failure prior to or during the PET scan the subject will be invited back for a replacement session (the total dose will then be ~7.5mSv, and the risks of this will be explained).

In Manchester, participants will be offered the option of having an extra, High Resolution Research Tomograph (HRRT) scan after their main STRATA PET scan (after a 15 minute comfort break). This will be between 30-60 minutes depending upon participant tolerability. Due to the long half-life of 18F and the slow removal of 18F from the brain, this extra scan will not involve any further injection of radiotracer. Another transmission scan will be carried out for attenuation correction purposes although this will be of very low radiation dose (0.02mSv). In the event of significant head movement during the HRRT scan, this transmission scan may be repeated.

In order to minimise the peripheral breakdown of 18F-DOPA, an oral dose of 150mg carbidopa and 400mg entacapone will be given one hour prior to the scan. Very few people experience any side-effects from these. Very occasionally people experience stomach upset, muscle movements, dry mouth and/or an orange tinge to their urine from the tablets, which may last a few hours to a day. This permits the use of a lower dose of 18F-DOPA than would otherwise be necessary. Participants will be asked to refrain from eating, drinking (apart from water) and smoking from midnight on the night before the scan, until after the scan is finished. This is because large amino acids may affect brain uptake of 18F-DOPA. Participants will also be instructed not to take illicit drugs (such as cannabis or cocaine) in the prior three days. Before the scan we will ask for a urine sample to check whether substances that can affect the scan are in their system. Women of childbearing age will have a pregnancy test and will be required to use regular contraception prior to the scan. At the start of the scan we will give participants a radiotracer (which is mildly radioactive) to measure the brain dopamine system. At the end of the scan the cannula will be removed from their arm.

Participants taking part in a PET scan at Imanova (SLaM/KCL participants) will have an additional 1-2 tablespoons (up to 30ml) of blood taken through their cannula to measure natural blood chemicals (hormones and genes) that are connected to dopamine function.

Participants taking part in a PET scan in Manchester will have all their bloods taken at this point (up to 50ml) as described under 'Biological Samples Section', whenever possible.

3. Sample Size, Statistics, Selection and Withdrawal of Subjects

The patients will be identified by members of the clinical team. Only the clinical team (who may also be part of the research team with NHS honorary contracts) will be able to access participant records and data prior to consent. No patient records will be screened by study researchers prior to consent. Study researchers will have access to patient records after/ if participants have consented to this.

We will recruit a total of 100 participants. Potential participants may be referred via clinical teams or other research studies/existing databases with consent to re-contact or registries and recruitment initiatives in NHS Trusts whose terms are in accordance with NHS Trust policies.

For peer review only - http://bmjopen.bmj.com/site/about/guidelines.xhtml

STRATA: Investigating factors associated with response to antipsychotic treatment
Protocol. Version 4.0, 19th August 2016
REC REF: 15/LO/0038

Participants will be first approached by a member of the clinical team or a member of the research team, with approval from the clinical team/ other study

Inclusion Criteria

1) aged 18-65;
2) DSM 5 schizophrenia/schizophreniform disorder.
3) Participants must read and write in English at a level sufficient to understand and complete study-related procedures

Exclusion Criteria

1) Pregnancy;
2) Severe head injury involving loss of consciousness >5 minutes (ever);
3) Meeting ICD criteria for harmful substance misuse or psychotic disorder secondary to substance misuse;
4) Participation in MRI scans requires exclusion of contraindications to MRI at 3 tesla e.g. metallic or electronic implants;
5) Severe claustrophobia.
6) Treatment with clozapine in the last 3 months

To establish and confirm the stratifier 1H-MRS data will be acquired in a total of 100 patients early in the course of their treatment; 50 T-Resp and 50 T-NonResp; matched for chronicity of illness.

Operational definition of T-Resp:
(i) treatment with only one antipsychotic drug since onset, or treatment changes have been due to adverse effects, not for non-response. (ii) CGI-SCH severity score of <4; (iii) PANSS total <60 (Leucht 2005); (iv) CRS >3

Operational definition of T-NonResp:
(i) documented treatment with at least two antipsychotics each above the minimum therapeutic dose as defined by the BNF for >4 weeks each; (ii) despite ongoing treatment and adequate adherence (assessed by iv) a CGI-SCH severity score of >3; (iii). PANSS total severity rating of at least 70 iv) Clinician Rating Scale (CRS; a measure of adherence) (Kemp et al 1996) >3.

Power and sample size calculation:
The study is powered to give >95% power to detect differences found in Egerton et al 2012 (α=0.05, allowing for 10% loss of sensitivity due to combining data from multiple centres). We have more than 80% power to detect a significant difference between a ROC curve with AUC 0.7 and chance, assuming α=0.05, 2-tailed. Two-tailed 18F-DOPA PET data will be acquired in a subset at 2 sites (N=60) to determine if the double dissociation between DA function and GLU function we have seen in chronic patients is also evident early in the illness course, where the strategy is most likely to be used (T-Resp n=30, T-NonResp n=30; powered to give >95% power to detect differences found in Demjaha et al 2012; α=0.05, allowing for 10% loss of sensitivity due to combining data from multiple centres).

Summary statistics will be used to describe the demographic and clinical characteristics of each participant group.

For peer review only - http://bmjopen.bmj.com/site/about/guidelines.xhtml

Group differences in demographic, clinical variables and 18F-DOPA Ki and glutamate concentration will be determined using pre-specified between group comparisons as appropriate (e.g. Chi square; Fischer's Exact; ANOVA).

Missing data will be minimal given that data is being collected prospectively. The exact reason for the missing data will be recorded. Any blank measures or spurious data will be checked against the paper copy of the CRF stored securely at sites.

Participants will be clearly told they can withdraw from the study at any time without having to give a reason. This is clear in the information sheet and the researcher will also explain this verbally to participants during the informed consent process. If a participant wishes to withdraw from a study all their identifiable data will be destroyed. Data or tissue already collected with the consent, which is not identifiable, would be retained and used in the study. No further data or tissue would be collected or any other research procedures carried out on or in relation to the participant.

Control group
We will recruit up to 15 healthy volunteers aged 18-65 to be scanned at each PET site (two sites; Imanova Limited, Imperial College London and The Wolfson Molecular Imaging Centre in Manchester) and 10 healthy volunteers aged 18-65 to be scanned at each MRI site (4 sites). This is to determine inter-site scanner variability and to provide normal range data for comparison with the clinical groups. In addition to the exclusion criteria above, healthy volunteers will be excluded if there is a history of schizophrenia or other psychotic disorder. Healthy volunteers will be recruited using an existing database of interested potential participants held at KCL.

4. Study procedures

Informed Consent

1) Participants will be first approached by a member of the clinical team or a member of the research team, with approval from the clinical team.
2) The study will be described verbally to potential participants and they will be given a copy of the information sheet. They will be encouraged to ask questions about the research. Potential participants will be allowed as much time as they require to make a decision and at least 24 hours so they are able to seek advice from others about participation, including previous participants in the research where possible.
3) If a patient expresses an interest in taking part, capacity to consent will be assessed and documented by the research team, in consultation with the clinical team.
4) If the patient has capacity to consent and agrees to participate in the study, they will be asked to sign and date two copies of the consent form. One copy will be kept by the participant and one by the research team. The research team will pass onto the clinical team to scan into medical notes, or incorporate in paper notes.
5) The participant will be informed that they can withdraw consent at any time, and without giving a reason.
6) Participants will be informed they are to be compensated for their time and travel expenses. This monetary amount will be up to £120 (£145 in Manchester) depending on which parts of the study the participant is involved with.

For peer review only - http://bmjopen.bmj.com/site/about/guidelines.xhtml

STRATA: Investigating factors associated with response to antipsychotic treatment
Protocol. Version 4.0, 19th August 2016
REC REF: 15/LO/0038

Within Avon and Wiltshire Mental Health Partnership NHS Trust, Everyone Included will be used to identify potentially eligible participants. Potentially eligible participants are identified based on the study inclusion /exclusion criteria, excluding those who have declined to receive information. This is done via an automated search of the Trust's electronic patient record system (RiO). An authorised search will be requested by a member or the R&D department, who are part of the clinical team and carried out by a member of the Information Analysis team. A data set is returned directly to the Everyone Included Administrators for processing the letters. No patient identifiable data will ever leave the Trust or be accessed by an external research team during this process.

The 'Research Opportunity Letter' will be sent to these individuals. The letter itself will not contain any patient identifiable or disclosing information (such as making reference to their diagnosis or medications). It will provide a free-post return slip and contact details (phone, email, website, postal address) inviting individuals to get in touch if they would like to further information / to take part. The onus is on the individual to express an interest, otherwise no further action is taken.

Upon responding to the 'Research Opportunity Letter', a Participant Information Sheet will be provided. If the research team is external, the individual will be asked if they are happy for their details (i.e. name and phone number) to be passed directly to the research team. No information is ever accessed by or passed to an external research team without first gaining permission from the potential participant. At this point standard study recruitment processes proceed.

Risks and burdens
The questionnaires involve personal questions and recalling experiences that some people may find distressing. Participants will be told if they feel uncomfortable with any of the questions they do not have to answer them.

Blood sampling and placing the cannula can cause some discomfort, and there is a possibility that a small bruise may develop. This task will be performed by research workers trained in phlebotomy. Any risks of infection will be contained by using standard sterile procedures and the risks associated with this task will be the same as for any other blood sample collection.

Any participants who become distressed during any procedure involved in this study will be encouraged to pause and will be reminded routinely that they can withdraw from the study at any time without a reason or penalty.

Any clinically significant issues that may arise during the assessment, the verbal consent will be obtained from the patient to pass onto the responsible psychiatrist or other relevant member of the staff. This will always be done with the participants' permission and will only be breached in the rare cases when there is judged to be an issue of safety, for example if the participant makes specific threats towards an individual.

Imaging
The MRI and PET scans themselves are painless and safe. Some people find the scans claustrophobic or anxiety-provoking. There is a mock scanner that participants can try out first if they wish. Participants will be told if they feel uncomfortable the scanning can be stopped at any time. Before the scan we will go through a safety questionnaire, to check that participants can have the scan. If they have any metal in their body, either from accidents or operations, they may

For peer review only - http://bmjopen.bmj.com/site/about/guidelines.xhtml

not be able to have the MRI scan, but they can still take part in the rest of the study. Clinical Research workers and research workers will log screening results.

Very occasionally people experience side effects from the medication they receive when taking part in a PET scan. These side-effects can include stomach upsets, muscle movements, dry mouth and/or an orange tinge to their urine. These side-effects may last a few hours to a day but participants will be warned about this and told it is nothing to worry about.

PET scans involve a small amount of radiation. Any exposure to radiation carries a risk of damaging the body's tissues and possibly triggering cancer at a later date. However, the risk is very small. A standard PET scan in this study will expose participants to 3.7mSv, (this may be 3.72mSv in Manchester if participants decide to have the extra, high resolution PET scan), which is the same amount of radiation that they are exposed to from natural sources of radiation, such as the sun, over the course of 18 months. In extremely rare cases the PET scan may need to be repeated and we have ARSAC approval for a maximum of 7.5mSv exposure per participant. Most experts believe that the risk of cancer developing only becomes significant in people who are exposed to 100mSv or more. However, as a precaution we are excluding pregnant or breastfeeding women. A pregnancy test will be carried out on female urine samples before the PET scan is conducted. Participants will be asked to consent to this on the consent form. Clinical Research workers and research workers will log screening results and ensure participants will not be exposed to more than 10mSv in 12 months (ARSAC guidelines suggest 10mSv as the normal upper limit for radiation exposure related to research procedure)

5. Sample handling and laboratories

Biological sample collection tubes and barcodes will be sent to sites in advance from the MRC Center for Neuropsychiatric Genetics and Genomics, Cardiff University. Samples will be stored in laboratories at sites and transportation will be organized when required (likely at 6 monthly basis, dependent on recruitment). Details of sample collection and storage at site will be recorded. Study SOPs will describe collection and storage specifications to ensure all sites are following the same guidelines.

When samples arrive at Cardiff University, researchers will ensure that the physical integrity of these samples have not been compromised in transit and track the samples in using their barcodes. The research team at Cardiff will notify the sponsor and the other study teams of any issues in transportation.

Cardiff University will extract DNA from the blood. We will perform genome-wide and targeted genotyping and/or exome or whole genome sequencing. We will seek genetic association with the imaging and other outcome measures at the level of individual genotype/sequence variant, genes, gene sets/pathways and polygenic or other summary scores.

A urine and blood sample will undergo metabolomic analysis at MRC National Phenome Centre. The Centre is funded by the MRC and NIHR and led by Imperial College London and King's College London**.** An additional blood sample will undergo proteomic analysis at the University of Manchester.

KCL/South London and the Maudsley (SLaM) participants will also be invited to participate in the BRC Biobank. This is covered by a separate ethical approval (09/H0606/84 NRES Committee South Central-Oxford C.)

For peer review only - http://bmjopen.bmj.com/site/about/guidelines.xhtml

6. Assessment of Safety

There are no serious adverse events expected to occur during the study.

All blood samples will only be taken by researchers trained in phlebotomy. All risks are the same as for any routine blood sample and are therefore minimal.

The drugs administered and the radiotracer used for the PET are standard procedures. The drugs administered may cause stomach upsets, muscle movements, dry mouth and/or an orange tinge to their urine. These side-effects may last a few hours to a day but participants will be warned about this and told it is nothing to worry about. Female participants will have a pregnancy test in advance.

For MRI scans a safety questionnaire will be carried out prior to the scan to check the participant does not have any metal in their bodies from operations or accidents.

7. Study oversight arrangements

STRATA is a multi-centred study and this will be managed by attendance at a monthly Consortium Executive meeting which will be responsible for the effective oversight of the daily activities of the study. Quarterly Consortium Board (CB) teleconferences will oversee the progress of, and interaction between, the workstreams to maintain communication of issues and progress between sites across the different aspects of STRATA. The CB will submit six-monthly Programme reports to the funder, MRC.

The project team consists of a full time Project Manager based at the IoPPN, KCL and a 50% Project Manager at the University of Manchester.

8. Ethics & Regulatory Approvals

REC name and address: South East Coast-Surrey Research Ethics Committee, Whitefriars, Level 3, Block B, Lewins Mead, Bristol, BS1 2NT

This study has also been reviewed and approved by the Administration of Radioactive Substances Advisory Committee (ARSAC).

9. Data Handling

Once participants have consented to be in the study some personal details will be taken. These details will be taken by the researcher with full consent to do so. These details will be kept securely at sites and used to contact patients when required to make appointments. No personal data will be shared with anyone outside of that study team. Each participant will be given a unique identifier and any clinical or genetic or imagining data relating to the same participant will link via that code.

Data will be entered and stored on a secure web application called Research Electronic Data Capture (REDCap). REDCap will not store any personal details and all participants will have a unique non-identifiable ID code. This unique ID code will then be used to merge all processed imaging, genetics and clinical data. REDCap will be hosted on secure servers at the Biomedical

For peer review only - http://bmjopen.bmj.com/site/about/guidelines.xhtml

Research Centre at Kings College London. All sites can access REDCap for the purposes of data entry via a web browser and data is uploaded when a WIFI signal is available.

The CI will ensure that the trial is conducted in compliance with the principles of the Declaration of Helsinki (1996), and in accordance with all applicable regulatory requirements including but not limited to the Research Governance Framework, Trust and Research Office policies and procedures and any subsequent amendments.

10. Finance and Publication Policy

STRATA is funded by a £ £4,900,000 Medical Research Council grant. Kings College London will receive and manage this funding. A collaboration agreement has agreed budgets between sites.

Analysis and findings from the study will be published as papers in journals. No identifiable data will be included.

This study has been adopted onto the UKCRN Portfolio and the research project will be registered on their database which is publicly available.

For peer review only - http://bmjopen.bmj.com/site/about/guidelines.xhtml

STROBE Statement—checklist of items that should be included in reports of observational studies

	Item No	Recommendation	Noted on pg.
**Title and abstract**	1	(*a*) Indicate the study's design with a commonly used term in the title or the abstract	1,2
		(*b*) Provide in the abstract an informative and balanced summary of what was done and what was found	1,2
**Introduction**			
Background/rationale	2	Explain the scientific background and rationale for the investigation being reported	4
Objectives	3	State specific objectives, including any prespecified hypotheses	5
**Methods**			
Study design	4	Present key elements of study design early in the paper	5
Setting	5	Describe the setting, locations, and relevant dates, including periods of recruitment, exposure, follow-up, and data collection	5
Participants	6	(*a*) *Cohort study*—Give the eligibility criteria, and the sources and methods of selection of participants. Describe methods of follow-up<br>*Case-control study*—Give the eligibility criteria, and the sources and methods of case ascertainment and control selection. Give the rationale for the choice of cases and controls<br>*Cross-sectional study*—Give the eligibility criteria, and the sources and methods of selection of participants	6,7
		(*b*) *Cohort study*—For matched studies, give matching criteria and number of exposed and unexposed<br>*Case-control study*—For matched studies, give matching criteria and the number of controls per case	N/A
Variables	7	Clearly define all outcomes, exposures, predictors, potential confounders, and effect modifiers. Give diagnostic criteria, if applicable	7,8
Data sources/ measurement	8*	For each variable of interest, give sources of data and details of methods of assessment (measurement). Describe comparability of assessment methods if there is more than one group	7,8
Bias	9	Describe any efforts to address potential sources of bias	8
Study size	10	Explain how the study size was arrived at	13
Quantitative variables	11	Explain how quantitative variables were handled in the analyses. If applicable, describe which groupings were chosen and why	7,8
Statistical methods	12	(*a*) Describe all statistical methods, including those used to control for confounding	8
		(*b*) Describe any methods used to examine subgroups and interactions	8
		(*c*) Explain how missing data were addressed	N/A
		(*d*) *Cohort study*—If applicable, explain how loss to follow-up was addressed<br>*Case-control study*—If applicable, explain how matching of cases and controls was addressed<br>*Cross-sectional study*—If applicable, describe analytical methods taking account of sampling strategy	N/A
		(*e*) Describe any sensitivity analyses	8

For peer review only - http://bmjopen.bmj.com/site/about/guidelines.xhtml

Continued on next page

For peer review only

For peer review only - http://bmjopen.bmj.com/site/about/guidelines.xhtml

**Results**			
Participants	13*	(a) Report numbers of individuals at each stage of study—eg numbers potentially eligible, examined for eligibility, confirmed eligible, included in the study, completing follow-up, and analysed	6,7,8
		(b) Give reasons for non-participation at each stage	6
		(c) Consider use of a flow diagram	N/A
Descriptive data	14*	(a) Give characteristics of study participants (eg demographic, clinical, social) and information on exposures and potential confounders	8,9
		(b) Indicate number of participants with missing data for each variable of interest	N/A
		(c) *Cohort study*—Summarise follow-up time (eg, average and total amount)	N/A
Outcome data	15*	*Cohort study*—Report numbers of outcome events or summary measures over time	N/A
		Case-control study—Report numbers in each exposure category, or summary measures of exposure	N/A
		Cross-sectional study—Report numbers of outcome events or summary measures	10,11
Main results	16	(*a*) Give unadjusted estimates and, if applicable, confounder-adjusted estimates and their precision (eg, 95% confidence interval). Make clear which confounders were adjusted for and why they were included	11
		(*b*) Report category boundaries when continuous variables were categorized	N/A
		(*c*) If relevant, consider translating estimates of relative risk into absolute risk for a meaningful time period	N/A
Other analyses	17	Report other analyses done—eg analyses of subgroups and interactions, and sensitivity analyses	Supplementary
**Discussion**			
Key results	18	Summarise key results with reference to study objectives	12
Limitations	19	Discuss limitations of the study, taking into account sources of potential bias or imprecision. Discuss both direction and magnitude of any potential bias	12, 13, 14
Interpretation	20	Give a cautious overall interpretation of results considering objectives, limitations, multiplicity of analyses, results from similar studies, and other relevant evidence	12, 13, 14
Generalisability	21	Discuss the generalisability (external validity) of the study results	13
**Other information**			
Funding	22	Give the source of funding and the role of the funders for the present study and, if applicable, for the original study on which the present article is based	15

*Give information separately for cases and controls in case-control studies and, if applicable, for exposed and unexposed groups in cohort and cross-sectional studies.

Note: An Explanation and Elaboration article discusses each checklist item and gives methodological background and published examples of transparent reporting. The STROBE checklist is best used in conjunction with this article (freely available on the Web sites of PLoS Medicine at http://www.plosmedicine.org/, Annals of Internal Medicine at http://www.annals.org/, and Epidemiology at http://www.epidem.com/). Information on the STROBE Initiative is available at www.strobe-statement.org.

For peer review only

BMJ Open

A cross sectional study comparing cognitive function in treatment responsive versus treatment non-responsive schizophrenia: evidence from the STRATA study.

Journal:	*BMJ Open*
Manuscript ID	bmjopen-2021-054160.R2
Article Type:	Original research
Date Submitted by the Author:	29-Sep-2021
Complete List of Authors:	Millgate, Edward; King's College London Institute of Psychiatry Psychology and Neuroscience, Department of Psychosis Studies<br>Kravariti, Eugenia; King's College London Institute of Psychiatry Psychology and Neuroscience, Department of Psychosis Studies; NIHR Biomedical Research Centre at Guy's and Saint Thomas' NHS Foundation Trust and King's College<br>Egerton, Alice; King's College London Institute of Psychiatry Psychology and Neuroscience, Department of Psychosis Studies; NIHR Biomedical Research Centre at Guy's and Saint Thomas' NHS Foundation Trust and King's College<br>Howes, Oliver; King's College London Institute of Psychiatry Psychology and Neuroscience, Department of Psychosis Studies; NIHR Biomedical Research Centre at Guy's and Saint Thomas' NHS Foundation Trust and King's College<br>Murray, Robin; King's College London Institute of Psychiatry Psychology and Neuroscience, Department of Psychosis Studies; NIHR Biomedical Research Centre at Guy's and Saint Thomas' NHS Foundation Trust and King's College<br>Kassoumeri, Laura; King's College London Institute of Psychiatry Psychology and Neuroscience, Department of Psychosis Studies; NIHR Biomedical Research Centre at Guy's and Saint Thomas' NHS Foundation Trust and King's College<br>Donocik, Jacek; King's College London Institute of Psychiatry Psychology and Neuroscience, Department of Psychosis Studies<br>Lewis, Shon; The University of Manchester Division of Psychology and Mental Health; Greater Manchester Mental Health NHS Foundation Trust<br>Drake, Richard; The University of Manchester Division of Psychology and Mental Health; Greater Manchester Mental Health NHS Foundation Trust<br>Lawrie, Stephen; The University of Edinburgh Division of Psychiatry, Psychiatry<br>Murphy , Anna; The University of Manchester Division of Psychology and Mental Health; Greater Manchester Mental Health NHS Foundation Trust<br>Collier, Tracy; King's College London Institute of Psychiatry Psychology and Neuroscience, Department of Psychosis Studies<br>Lees, Jane; The University of Manchester Division of Psychology and Mental Health; Greater Manchester Mental Health NHS Foundation Trust<br>Stockton-Powdrell, Charlotte; The University of Manchester Division of Psychology and Mental Health<br>Walters, James; Cardiff University

	Deakin, JFW; The University of Manchester Division of Psychology and Mental Health<br>MacCabe, James; King's College London Institute of Psychiatry Psychology and Neuroscience, Department of Psychosis Studies; NIHR Biomedical Research Centre at Guy's and Saint Thomas' NHS Foundation Trust and King's College
**Primary Subject Heading**:	Mental health
Secondary Subject Heading:	Pharmacology and therapeutics
Keywords:	Schizophrenia & psychotic disorders < PSYCHIATRY, PSYCHIATRY, Adult psychiatry < PSYCHIATRY

SCHOLARONE™
Manuscripts

I, the Submitting Author has the right to grant and does grant on behalf of all authors of the Work (as defined in the below author licence), an exclusive licence and/or a non-exclusive licence for contributions from authors who are: i) UK Crown employees; ii) where BMJ has agreed a CC-BY licence shall apply, and/or iii) in accordance with the terms applicable for US Federal Government officers or employees acting as part of their official duties; on a worldwide, perpetual, irrevocable, royalty-free basis to BMJ Publishing Group Ltd ("BMJ") its licensees and where the relevant Journal is co-owned by BMJ to the co-owners of the Journal, to publish the Work in this journal and any other BMJ products and to exploit all rights, as set out in our licence.

The Submitting Author accepts and understands that any supply made under these terms is made by BMJ to the Submitting Author unless you are acting as an employee on behalf of your employer or a postgraduate student of an affiliated institution which is paying any applicable article publishing charge ("APC") for Open Access articles. Where the Submitting Author wishes to make the Work available on an Open Access basis (and intends to pay the relevant APC), the terms of reuse of such Open Access shall be governed by a Creative Commons licence – details of these licences and which Creative Commons licence will apply to this Work are set out in our licence referred to above.

Other than as permitted in any relevant BMJ Author's Self Archiving Policies, I confirm this Work has not been accepted for publication elsewhere, is not being considered for publication elsewhere and does not duplicate material already published. I confirm all authors consent to publication of this Work and authorise the granting of this licence.

For peer review only

Journal: BMJ Open

Word count: 3083 *(Table word count = 490)*

Abstract word count: 293

Submission date: 3rd June 2021

A cross sectional study comparing cognitive function in treatment responsive versus treatment non-responsive schizophrenia: evidence from the STRATA study.

Edward Millgate[1], Eugenia Kravariti[1,2], Alice Egerton[1,2], Oliver D Howes[1,2], Robin M Murray[1,2], Laura Kassoumeri[1,2], Jacek Donocik[1], Shôn Lewis[3,4], Richard Drake[3,4], Stephen M Lawrie[5], Anna Murphy[3,4], Tracy Collier[1], Jane Lees[3,4], Charlotte Stockton-Powdrell[3], James Walters[6], Bill Deakin[3,4] and James H MacCabe[1,2]*

[1] Institute of Psychiatry, Psychology and Neuroscience, King's College London, United Kingdom

[2] NIHR Biomedical Research Centre at South London and Maudsley NHS Foundation Trust, London, United Kingdom

[3] Division of Psychology and Mental Health, School of Biological Sciences, Faculty of Biology, Medicine and Health, University of Manchester, Manchester

[4]Greater Manchester Mental Health NHS Foundation Trust, Manchester, United Kingdom

[5]Division of Psychiatry, University of Edinburgh, Edinburgh, United Kingdom

[6]Division of Psychological Medicine and Clinical Neuroscience, Cardiff University, Cardiff, United Kingdom

*Corresponding author: james.maccabe@kcl.ac.uk

Department of Psychosis Studies,

Institute of Psychiatry, Psychology & Neuroscience,

16 de Crespigny Park,

Denmark Hill, London,

United Kingdom, SE5 8AF

For peer review only - http://bmjopen.bmj.com/site/about/guidelines.xhtml

Abstract

Background: 70-84% of individuals with antipsychotic treatment resistance show non-response (NR) from the first episode. Emerging cross-sectional evidence comparing cognitive profiles in treatment resistant schizophrenia to treatment-responsive schizophrenia has indicated that verbal memory and language functions may be more impaired in treatment resistance. We sought to confirm this finding by comparing cognitive performance between antipsychotic non-responders (NR) and responders (R) using a brief cognitive battery for schizophrenia, with a primary focus on verbal tasks compared against other measures of cognition.

Design: Cross-sectional

Setting: This cross-sectional study recruited antipsychotic treatment responders (R) and antipsychotic non-responders (NR) across four UK sites. Cognitive performance was assessed using the Brief Assessment of Cognition in Schizophrenia (BACS).

Participants: One hundred and six participants aged 18 – 65 years with a diagnosis of schizophrenia or schizophreniform disorder were recruited according to their treatment response, with 52 NR, and 54 R cases.

Outcomes: Composite and subscale scores of cognitive performance on the BACS. Group (R vs NR) differences in cognitive scores were investigated using univariable and multivariable linear regressions adjusted for age, gender and illness duration.

Results: Univariable regression models observed no significant differences between R and NR groups on any measure of the BACS, including verbal memory (95% CI -6.63 to 2.66, $p = .398$) and verbal fluency (95% CI -2.46 to 4.91, $p = .510$). This pattern of findings was consistent in multivariable models.

Conclusions: The lack of group difference in cognition in our sample is likely due to a lack of clinical distinction between our groups. Future investigations should aim to utilise machine learning methods using longitudinal first episode samples to identify responder subtypes within schizophrenia, and how cognitive factors may interact within this.

Trail registration number: REC: 15/LO/0038.

Keywords: cognition, treatment resistance, antipsychotic response, schizophrenia, BACS

For peer review only

Article summary

Strengths and limitations of this study

- The study examined cognitive performance in a relatively large and multicentre sample of antipsychotic responders and non-responders
- Cognition was assessed with the BACS, a reliable and brief test battery specifically designed for schizophrenia
- The lack of significant group differences in cognition between antipsychotic responders and non-responders may reflect limited clinical separation between these groups.

For peer review only

Introduction

Up to a third of patients with a schizophrenia diagnosis have inadequate symptomatic improvement despite having at least two antipsychotic drugs, one being a second-generation antipsychotic excluding clozapine, at adequate doses and duration (4 – 6 weeks; NICE guidelines) [1] and are termed treatment resistant (TRS) [2,3]. Almost all guidelines recommend the antipsychotic clozapine in TRS [4] with earlier clozapine treatment associated with better outcomes [5-8]. There is increasing evidence that TRS may represent a distinct subtype in schizophrenia [9,10]. Most treatment resistant cases exhibit antipsychotic non-response (NR) from the first episode with this observed in 70-84% of patients [3,11]. An earlier age of onset has also been consistently associated with antipsychotic treatment resistance [12-16], suggesting that TRS and NR may be associated with neurodevelopmental impairment. Identifying these underlying factors associated with antipsychotic treatment resistance in schizophrenia is therefore important for improving prediction and early treatment of NR and TRS.

Cognitive impairment in schizophrenia may provide some insight into antipsychotic treatment response. Performance on tasks of verbal memory has often been reported to be impaired in schizophrenia samples [17], those prior to medication initiation [18], and at first episode [19,20]. Indeed, impairments in verbal memory and language functions have also been reported in unaffected first-degree relatives of schizophrenia patients relative to healthy controls [21,22]. Verbal memory and verbal working memory functions have also been reported to show a protracted maturation into adulthood, with impairments in these functions observed in both early and late schizophrenia [23]. This suggests a possibility of a genetic and cognitive continuum of risk in schizophrenia, which increases from controls to first-degree relatives, to treatment responsive schizophrenia. A broader hypothesis is that treatment resistance is etiologically continuous with treatment responsive schizophrenia but occupies a more exaggerated position on a continuum of neurodevelopmental liability.

In a recent meta-analysis comparing mostly cross-sectional studies of treatment resistant cases and responders, TRS cases exhibited greatest cognitive impairments on tasks of verbal memory and learning (dl = -0.59, p <.001) and language functions (dl = -0.53, p <.001), with smaller but still statistically significant impairments in tasks across other cognitive domains, relative to their responder counterparts [24]. However, this meta-analysis

For peer review only - http://bmjopen.bmj.com/site/about/guidelines.xhtml

included an array of cognitive tasks, many with long test duration and stringent training requirements for raters. Short and comprehensive measures of cognitive performance may aid in the detection of neuropsychological differences between antipsychotic responders (R) and non-responders (NR), while also being cost-effective. The Brief Assessment of Cognition in Schizophrenia (BACS) [25] was originally developed to be an easily administrable, brief, test battery that efficiently and specifically assesses cognitive deficits in schizophrenia cases. The measures included in the battery correspond to several cognitive domains with established deficits in schizophrenia; executive functions [26,27], working memory [28,29], motor/processing speed [30], verbal memory [31,32], verbal fluency [33,34] and attention [35,36]. If observable differences between antipsychotic responders and non-responders are identified, this would further improve our understanding of cognitive factors implicated in the aetiology of antipsychotic response. Likewise, this would raise the possibility for future prospective research to use brief cognitive testing as part of predictive/diagnostic models for antipsychotic response and future treatment resistance.

Therefore, this cross-sectional study sought to assess the cognitive profiles of antipsychotic responders and non-responders utilising the Brief Assessment of Cognition in Schizophrenia. Based on the existing literature, we hypothesised that TRS patients would have poorer performance across BACS tasks, particularly on verbal memory and verbal fluency measures.

Methods

Design

The study used a cross-sectional design comparing antipsychotic treatment responders (R) and antipsychotic non-responders (NR) on cognitive performance.

Setting

The study was part of ‘Schizophrenia: Treatment Resistance and Therapeutic Advances’ (STRATA), a consortium which included King’s College London (London, UK), University of Manchester (Manchester, UK), Cardiff University (Wales, UK) and University of Edinburgh (Scotland, UK). The aim of the STRATA consortium is to identify

For peer review only - http://bmjopen.bmj.com/site/about/guidelines.xhtml

For peer review only

neurobiological, cognitive and genetic biomarkers of antipsychotic treatment resistance and non-response within schizophrenia and other related psychotic disorders.

Patient and Public Involvement

During the early development of the study the views and recommendations of service users and carers regarding the use of stratified medicine research were assessed. Consultations were undertaken with the Institute of Psychiatry, Psychology and Neuroscience's Service User Advisory Group (SUAG). Service user researchers in London, Manchester, and Edinburgh (18 people) carried out focus groups, and one carer focus group in London (8 people). Focus groups were digitally recorded, the transcripts analysed in NVivo 10 using a simple thematic analysis, and quotations de-identified to protect participants. The results of this research are published in BioMed Central [37]. Both service users and carers reflected enthusiasm for stratified medicine. Each stage of the study was discussed, including their willingness to participate and attitudes towards, and perceived intrusiveness of different procedures. These individuals also aided in commenting and providing recommendations on consent and participant information forms.

Participants

One hundred and six participants were recruited following a screening of patients across four sites: King's College London (N = 38), University of Manchester (N = 32), Cardiff University (N = 16) and University of Edinburgh (N = 18). Inclusion criteria were as follows: aged 18 – 65 years, with a schizophrenia or schizophreniform disorder diagnosis as per Diagnostic and Statistical Manual of Mental Disorders, 5th edition (DSM-5) [38] criteria and be able to read and write English to a sufficient level (see also Egerton et al [39]). Participants were excluded if they were pregnant, had ever experienced a head injury involving loss of consciousness for more than 5 minutes, met ICD criteria for harmful substance misuse or a psychotic disorder secondary to substance use, scored < 3 on the Clinical Rating Scale (a measure of adherence) [40,41], or had been treated with clozapine in the previous three months. All participants gave informed consent prior to enrolment.

Ethical approval

For peer review only - http://bmjopen.bmj.com/site/about/guidelines.xhtml

This study was approved by the South East Coast-Surrey Research Ethics Committee; REC: 15/LO/0038. All participants provided informed consent prior to participation.

Definition of antipsychotic response and antipsychotic non-response

Participants were defined as antipsychotic treatment responders (R) if they had been treated with only one antipsychotic drug since illness onset or if their antipsychotic drug had been changed only for reasons of adverse effects as opposed to non-response. In addition to this, responders needed to have a Clinical Global Impression score (CGI-SCH) [42] of below 4 (*moderately ill*), a Positive and Negative Syndrome scale (PANSS) [43] total score below 60, and a Clinical Rating Scale (CRS) [40,41] level of adherence greater than 3 (*'accepts only because compulsory'*). Fifty-four treatment responders were recruited into the study.

Antipsychotic treatment non-response (NR) was defined as having documented treatment with at least two antipsychotics each above the minimum therapeutic dose as defined by the British National Formulary for > 4 weeks each, a CGI-SCH severity score of > 3, a PANSS total severity rating of at least 70, and a CRS adherence score of > 3. Fifty-two participants met criteria for antipsychotic non-response.

Materials

Clinical and demographic measures

Previous and existing drug use were measured using the Alcohol, Drug and Tobacco Inventory. Participants' disorder severity was measured using the Mini-International Neuropsychiatric Interview (M-Psychotic Disorders; A-Major Depressive Episode; D-Manic/Hypomanic/Bipolar; MINI) [44], Structured Clinical Interview- Positive and Negative Syndrome Scale (SCI-PANSS) [45] and Clinical Global Impression-Schizophrenia scale (CGI-SCH) [42]. Concordance with medication was assessed using the Clinical Rating Scale for Schizophrenia (CRS) [40,41]. Participants also provided demographic data, such as years of previous full-time education, age, gender, as well as information regarding their previous antipsychotic history which were supplemented by medical records.

Measures of cognitive performance

Cognitive data was collected using the Brief Assessment of Cognition in Schizophrenia (BACS) [25] across all sites at the beginning of the assessment, following the

For peer review only - http://bmjopen.bmj.com/site/about/guidelines.xhtml

administration of clinical and demographic measures. The battery is designed to take ~30 mins to complete, with minimal training demands, and is designed to be easily administered by clinical and healthcare workers [25]. The BACS (version A) [25] consists of six tests from the following cognitive domains: i) Verbal Memory: List learning task; ii) Working Memory: Digit Sequencing task; iii) Motor Speed: Token motor task; iv) Verbal Fluency: Category instances task (Animals) and phonological (F and S-words); v) Attention and speed of information processing: Symbol Coding task; vi) Executive Functions: Tower of London task. All tasks on the BACS are scored with higher scores representing better performance. Composite z and t scores for the BACS are generated using normative data [46] and the following formulas: $Composite\ z\ score = \frac{\Sigma\left(\Sigma\frac{(raw\ score - normative\ score)}{normative\ standard\ deviation}\right)}{3.63}$ with each measure's z score summed and the total divided by 3.63; $Composite\ t\ score = (Composite\ z\ score * 10) + 50$.

Data analysis

All analyses were conducted using STATA 15/SE [47]. Chi-square tests were used to compare cognitive performance across sites in case of site differences. Univariable regressions were used to compare cognitive performance between groups. Multivariable regression analyses were used to adjust univariable results for age, gender and illness duration, due to the reported relationship of age [48,49], gender [50,51] and illness duration [52,53] with cognitive outcomes. Analyses adjusting for anticholinergic effects of antipsychotic medication are presented in the supplementary material (Table S.1).

Results

Descriptive statistics of demographic and clinical variables between responder groups are reported in Table 1. In the antipsychotic responder group (N = 54), 4 were treated with a first-generation antipsychotic. For the non-responder group (N = 52), 5 were treated with a first-generation antipsychotic. All other participants were treated with second-generation antipsychotics.

For peer review only - http://bmjopen.bmj.com/site/about/guidelines.xhtml

Table 1

Demographic and clinical characteristics by group

	R			NR		
Demographic/clinical variable	N	Mean/ratio	SD	N	Mean/ratio	SD
Age	54	29.52	9.36	52	29.99	8.50
Gender (male : female)	54	46 : 8	-	52	43 : 9	-
Age of illness onset	53	26.10	6.53	50	25.31	5.93
Illness duration since 1st antipsychotic (years)	53	3.71	6.87	50	5.03	5.79
Duration from 1st psychotic symptom (years)	54	4.81	7.53	52	5.50	6.13
Duration from 1st contact with mental health services (years)	54	4.04	7.49	52	5.40	6.34
Full time education (years)	53	13.09	2.37	50	12.88	2.75
Chlorpromazine equivalents (mg/day)	53	305.45	146.86	52	343.73	202.83
PANSS positive score	54	12.24	3.40	42	22.65	3.54
PANSS negative score	54	13.82	3.38	52	20.96	4.56
PANSS total score	54	53.46	7.91	52	87.29	9.30
CGI positive symptoms score	53	3.26	.76	52	5.50	.10
CGI negative symptoms score	53	3.21	.86	52	4.88	1.04
CGI cognitive symptoms score	53	3.08	.83	52	4.83	1.22
CGI overall severity	53	3.42	.75	52	5.48	.58
Antipsychotic at assessment	54	Amisulpride = 3<br>Aripiprazole = 13<br>Clopixol = 2<br>Haloperidol = 1<br>Olanzapine = 19<br>Quetiapine = 4<br>Risperidone = 9<br>Flupentixol = 1<br>Paliperidone = 2	-	52	Amisulpride = 8<br>Aripiprazole = 10<br>Clopixol = 1<br>Haloperidol = 2<br>Olanzapine = 7<br>Quetiapine = 9<br>Risperidone = 6<br>Flupentixol = 1<br>Paliperidone = 6<br>Zuclopenthixol acetate = 1	-

For peer review only - http://bmjopen.bmj.com/site/about/guidelines.xhtml
For peer review only

Note. R = antipsychotic responder; NR = antipsychotic non-responder; PANSS = Positive and Negative Syndrome Scale; CGI = Clinical Global Impression.

Cognitive performance

Mean scores for each group on all BACS tasks and standardized composite scores are displayed in Table 2. All measures of the BACS were normally distributed, with exception of the Tower of London task which was moderately negatively skewed (skewness = -0.95) as per the guidelines from Bulmer [54]. Cognitive performance on BACS composite and subtests did not significantly differ by site where data was collected.

For peer review only - http://bmjopen.bmj.com/site/about/guidelines.xhtml

Table 2

Mean group performance on BACS measures and univariable and multivariable linear regression models for response status and BACS performance

	R			NR			Unadjusted				Adjusted for age, gender and illness duration			
BACS measure	N	Mean	SD	N	Mean	SD	β	SE	95%CI	P-value	β	SE	95%CI	P-value
Verbal Memory	53	38.89	10.66	50	36.9	13.04	-1.99	2.34	-6.63 ; 2.66	.398	-2.68	2.38	-7.41 ; 2.05	.263
Digit Sequencing	53	17.87	4.95	50	17.98	4.09	0.11	0.90	-1.67 ; 1.89	.901	0.21	0.92	-1.61 ; 2.03	.818
Verbal Fluency	53	30.45	9.04	50	31.68	9.82	1.23	1.86	-2.46 ; 4.91	.510	1.12	1.92	-2.70 ; 4.92	.563
Token Motor	53	66.32	14.56	49	65.90	15.26	-0.42	2.95	-6.28 ; 5.43	.886	-1.05	2.93	-6.87 ; 4.78	.723
Symbol Coding	53	47.30	11.31	50	45.46	11.83	-1.84	2.28	-6.37 ; 2.68	.421	-1.71	2.35	-6.37 ; 2.95	.469
Tower of London	53	16.04	4.46	50	16.44	3.83	0.40	0.82	-1.23 ; 2.03	.625	0.50	0.83	-1.16 ; 2.15	.552
z score composite	53	-2.00	1.39	49	-2.03	1.51	-0.03	0.29	-0.60 ; 0.54	.922	-0.04	0.30	-0.63 ; 0.56	.908
t score composite	53	29.91	13.81	49	29.27	14.99	-0.64	2.87	-6.32 ; 5.05	.825	-0.75	2.99	-6.69 ; 5.19	.804

Note. R = antipsychotic responder; NR = antipsychotic non-responder; BACS = Brief Assessment of Cognition in Schizophrenia; CIs = confidence intervals.

For peer review only - http://bmjopen.bmj.com/site/about/guidelines.xhtml

Univariable linear regression analyses (Table 2) observed no significant relationships between response status and BACS performance. Multivariable models adjusted for age, gender and illness duration also observed no significant relationships between response status and cognitive outcomes (Table 2).

Discussion

The present investigation sought to compare specific cognitive deficits in antipsychotic responders (R) and antipsychotic non-responders (NR) using the Brief Assessment of Cognition in Schizophrenia (BACS) [25], anticipating the greatest deficits for NR in measures of verbal memory and verbal fluency when compared to R. Unlike previous cross-sectional studies [55-62], this investigation identified no significant differences in cognitive performance between groups.

Previous cross sectional research investigating differences in cognitive performance between antipsychotic treatment responders and treatment resistant cases have identified poorer performance in verbal, executive function, full-scale IQ cognitive measures [55,56,59-61], and verbal memory [55,58,60,62,63] in treatment resistant patients. A recent study using a similar methodology and sample size to ours also failed to show significant differences between antipsychotic responders and TRS cases on individual tasks of the BACS [64] but did observe significant differences on standardized (z and t) composite scores suggesting overall impairment in the TRS group. Our additional analyses also adjusting for anticholinergic effects (supplementary material: Table S.1) also observed no change to the relationship between BACS and antipsychotic response, suggesting no medication effects on our findings. We also further restricted our analysis to exclude participants that were under dosed (i.e. not within the 150-600mg/per day range) removing 12 participants (R = 5, NR = 7). No change was observed in the pattern of results.

The lack of significant differences in cognitive performance observed between R and NR groups in our study may be partly explained by the criteria used to define these groups. Unlike earlier investigations, our study did not include clozapine-treated patients, and there may have been less clinical separation between the R and NR groups than in some previous studies (as discussed in Egerton et al [39]). Furthermore, in our cross-sectional study design it is not possible to determine the proportion of participants in the NR group who would meet

For peer review only - http://bmjopen.bmj.com/site/about/guidelines.xhtml

criteria for TRS [65]. It is therefore possible the non-responder group was less severely unwell as in some previous studies, which may have reduced the ability to observe potential impairments in cognition due to clinical overlap. Previous investigations which observed group differences in cognitive performance between R and TRS included patients prescribed clozapine [56,57,59-61,63,64], and reported higher PANSS positive, negative and total scores[59,60,64], suggesting the NR/TRS groups may have had greater illness severity compared to our sample. Likewise, demographic and clinical variables previously found to be associated with antipsychotic response, such as a younger age and age of illness onset in non-responders [12-16], did not differ between treatment responders and non-responders in our sample, again suggesting group that compared to previous investigations, there wasn't enough clinical separation between our samples. In addition, the power calculations for sample size were generated on the basis of being able to provide > 95% power to detect differences in levels of anterior cingulate glutamate [39] (see Protocol provided in supplementary material) and it is possible that the sample was underpowered to detect neurocognitive differences using the BACS.

It is also possible that our definition of antipsychotic response and inclusion criteria may have influenced our findings. As per definition, differences were only observed between groups on CGI-SCH and PANSS measures of symptom severity. Psychotic symptoms such as hallucinations, delusions and paranoia (i.e. schizophrenia-like symptoms) have been attributed to D2 dopamine receptors and functioning in the striatum, as evidenced by animal models [65]. It has also been reported that following amphetamine administration, hyperactivity of dopamine transition is associated with the activation of psychotic symptoms. However, amphetamine induced psychosis does not tend to exhibit negative and cognitive symptoms [66]. In contrast, cognitive deficits in schizophrenia have been reported to be related to functioning in the dorsolateral prefrontal cortex (DLPFC) [67,68], glutamate to GABA ratios in the DLPFC [69], as well as prefrontal glutamate levels in the dorsal anterior cingulate cortex in antipsychotic-naïve patients [69]. Unlike psychotic symptoms, the Dopamine D1 receptor signalling is essential for cognition [70]. Therefore, it is possible that the differences in the neurobiological underpinnings between psychotic and cognitive symptoms may also explain why no cognitive differences were observed between groups, as this was biased in favour of psychotic symptoms due to our inclusion criteria.

For peer review only - http://bmjopen.bmj.com/site/about/guidelines.xhtml

Another consideration is that our study focused on younger patients early in their treatment trajectories to reduce the potential effects of chronicity and previous medication, with a mean length of treatment of 3 to 4 years. Most previous cross-sectional investigations also include older samples with a longer duration of illness [56,57,59,60,64], although differences in measures of verbal intelligence and fluency have been quantifiable at the first episode in treatment resistant psychosis[27]. Trajectory modelling of cognitive performance in FEP has observed deficits in executive function performance, relative to controls, with these remaining stable over illness duration [71]. However, deficits in verbal knowledge and memory became more apparent and exaggerated relative to controls following the first episode [72]. Similar exaggerated declines following the first episode have also been observed in measures of verbal memory [71,73]. With our sample of patients being early in their treatment, cognitive deficits may have been less marked at this illness stage.

Likewise, this more restricted focus may explain why there was smaller sampling of females in comparison to previous investigations. A recent nation-wide cohort study found that on average females are more likely to be first diagnosed with a mood disorder prior to a psychotic diagnosis[74]. This coupled with the observation that females also tend to have a later onset of psychotic symptoms than males[75], it is possible that recruiting younger participants may have restricted the true picture of schizophrenia at large within the general population.

Despite not detecting significant differences between antipsychotic responder groups, it is worth mentioning the importance of conducting research using clinically transferable measures of cognitive impairment. It may be possible for future researchers to use machines learning algorithms to identify subgroups of schizophrenia from cognitive outcomes. Bak et al [76] used Gaussian mixture modelling to identify two distinct subgroups in antipsychotic-naive first episode schizophrenia samples. In this study, cognitive and electrophysiological data were used to identify the two groups. When predicting treatment response, assessed by the PANSS, there was a significant predictive relationship between group and antipsychotic response. Therefore, future research should aim to use more machine learning techniques to identify patterns of cognitive performance within schizophrenia subsamples and investigate antipsychotic response between these groups.

For peer review only - http://bmjopen.bmj.com/site/about/guidelines.xhtml

Conclusions

Within this cross-sectional investigation we observed no differences in cognitive performance between antipsychotic responders and non-responders. This may be because there was less clinical separation between these groups in our sample in comparison to previous investigations. Future investigations should consider the role of machine learning techniques to investigate the role of cognitive functions in identifying subgroups of schizophrenia using first episode cohorts and how this may differ in future stages of treatment resistance. Such research using antipsychotic-naïve patients versus healthy controls has observed strong group discrimination using cognitive measures in comparison to electrophysiology and magnetic resonance imaging methods [77], with other investigations observing distinct subgroups in schizophrenia from differences in early information processing and higher cognitive functions [74].

Funding

STRATA is funded by a grant from the Medical Research Council (MRC) to JHM, grant reference MR/L011794. E.M.'s PhD is funded by the MRC-doctoral training partnership studentship in Biomedical Sciences at King's College London. J.H.M., E.K., R.M.M., A.E., O.D.H. are part funded by the National Institute for Health Research (NIHR) Biomedical Research Centre at South London and Maudsley NHS Foundation Trust and King's College London. In the past three years, S.M.L. has received personal support from Sunovion. The views expressed are those of the authors and not necessarily those of the NHS, the MRC, the NIHR, Sunovion or the Department of Health.

Competing interest

The remaining authors report no conflicts of interest.

Acknowledgments

For more information on STRATA please visit; https://gtr.ukri.org/project/7F7F0378-8FD7-4A33-91AD-277A49EF4908). STRATA was funded by a grant from the Medical Research Council (MRC), to Prof James MacCabe. Data were collected between March 2015 and February 2017.

For peer review only - http://bmjopen.bmj.com/site/about/guidelines.xhtml

Data Availability

At the time of submission, the data governance frameworks are being put in place to make a fully anonymized version of the data available to the wider research community via the TranSMART data sharing platform: https://transmartfoundation.org/. To apply for access to the data, please contact J.H.M. at james.maccabe@kcl.ac.uk.

Author contributions

J.H.M., R.M.M., O.D.H., A.E., E.K., R.D., L.K., J.D., A.M., T.C., J.L., C.S.P., J.W., B.D. and S.M.L. contributed to the design and implementation of the study. E.M. completed analyses and wrote the manuscript with the assistance of J.H.M. and E.K.. J.H.M., R.M.M., O.D.H., A.E., E.K., S.M.L., J.D., S.L., provided comments on the manuscript.

References

For peer review only - http://bmjopen.bmj.com/site/about/guidelines.xhtml

1 National Institute for Health and Care Excellence, National Collaborating Centre for Mental Health (UK. Psychosis and schizophrenia in adults: treatment and management). 2014.

2 Elkis H. Treatment-resistant schizophrenia. *Psychiatric Clinics of North America*. 2007 Sep1;30(3):511-33.

3 Demjaha A, Lappin JM, Stahl D, et al. Antipsychotic treatment resistance in first-episode psychosis: prevalence, subtypes and predictors. *Psychological medicine*. 2017 Aug;47(11):1981-9.

4 Kane JM, Agid O, Baldwin ML, et al. Clinical guidance on the identification and management of treatment-resistant schizophrenia. *The Journal of clinical psychiatry*. 2019 Mar5;80(2):0-.

5 Üçok A, Çıkrıkçılı U, Ergül C, et al. Correlates of clozapine use after a first episode of schizophrenia: results from a long-term prospective study. *CNS drugs*. 2016 Oct;30(10):997-1006.

6 Köhler-Forsberg O, Horsdal HT, Legge SE, et al. Predictors of nonhospitalization and functional response in clozapine treatment: a nationwide, population-based cohort study. *Journal of clinical psychopharmacology*. 2017 Apr1;37(2):148-54.

7 Yoshimura B, Yada Y, So R, et al. The critical treatment window of clozapine in treatment-resistant schizophrenia: secondary analysis of an observational study. *Psychiatry research*. 2017 Apr1;250:65-70.

8 Griffiths K, Millgate E, Egerton A, et al. Demographic and clinical variables associated with response to clozapine in schizophrenia: a systematic review and meta-analysis. *Psychological Medicine*. 2021 Feb1:1-1.

9 Lally J, Gaughran F, Timms P, et al. Treatment-resistant schizophrenia: current insights on the pharmacogenomics of antipsychotics. *Pharmacogenomics and personalized medicine*. 2016;9:117.

10 Gillespie AL, Samanaite R, Mill J, et al. Is treatment-resistant schizophrenia categorically distinct from treatment-responsive schizophrenia? A systematic review. *BMC psychiatry*. 2017 Dec;17(1):1-4.

11 Lally J, Ajnakina O, Di Forti M, et al. Two distinct patterns of treatment resistance: clinical predictors of treatment resistance in first-episode schizophrenia spectrum psychoses. *Psychological medicine*. 2016 Nov;46(15):3231-40.

For peer review only - http://bmjopen.bmj.com/site/about/guidelines.xhtml

12 Wimberley T, Støvring H, Sørensen HJ, et al. Predictors of treatment resistance in patients with schizophrenia: a population-based cohort study. *The Lancet Psychiatry*. 2016 Apr1;3(4):358-66.

13 Smart SE, Kępińska AP, Murray RM et al. Predictors of treatment resistant schizophrenia: a systematic review of prospective observational studies. *Psychological medicine*. 2021 Jan;51(1):44-53.

14 Legge S, Dennison C, Pardinas A, et al. Clinical indicators of treatment-resistant psychosis. *British Journal of Psychiatry*. 2020 May31;216(5):259-66.

15 Bozzatello P, Bellino S, Rocca P. Predictive factors of treatment resistance in first episode of psychosis: a systematic review. *Frontiers in psychiatry*. 2019 Feb26;10:67.

16 Smart S. *Clinical and Genetic Predictors of Treatment Resistant Psychosis* (Doctoral dissertation, King's College London). 2020.

17 Toulopoulouand T, Murray RM. Verbal memory deficit in patients with schizophrenia: an important future target for treatment. *Expert review of neurotherapeutics*. 2004 Jan1;4(1):43-52.

18 Fatouros-Bergman H, Cervenka S, Flyckt L, et al. Meta-analysis of cognitive performance in drug-naïve patients with schizophrenia. *Schizophrenia research*. 2014 Sep1;158(1-3):156-62.

19 Mesholam-Gately RI, Giuliano AJ, Goff KP, et al. Neurocognition in first-episode schizophrenia: a meta-analytic review. *Neuropsychology*. 2009 May;23(3):315.

20 Kravariti E, Morgan K, Fearon P, et al. Neuropsychological functioning in first-episode schizophrenia. *The British Journal of Psychiatry*. 2009 Oct;195(4):336-45.

21 Snitz BE, MacDonald III AW, Carter CS. Cognitive deficits in unaffected first-degree relatives of schizophrenia patients: a meta-analytic review of putative endophenotypes.

22 Bora E, Özerdem A. Meta-analysis of longitudinal studies of cognition in bipolar disorder: comparison with healthy controls and schizophrenia. *Psychological medicine*. 2017 Dec;47(16):2753-66.

23 Fagerlund B, Pantelis C, Jepsen JR, Raghava JM, Rostrup E, Thomas MB, Nielsen MØ, Bojesen K, Jensen KG, Stentebjerg-Decara M, Klauber DG. Differential effects of age at illness onset on verbal memory functions in antipsychotic-naïve schizophrenia patients aged 12–43 years. *Psychological medicine*. 2020 Mar 11:1-1.

24 Millgate E, Hide O, Lawrie S, et al. Neuropsychological differences between treatment-resistant and treatment-responsive schizophrenia: a systematic review & meta-analysis.

For peer review only - http://bmjopen.bmj.com/site/about/guidelines.xhtml

[Manuscript submitted for publication]. Institute of Psychiatry, Psychology and Neuroscience, King's College London

25 Keefe RS, Goldberg TE, Harvey PD, et al. The Brief Assessment of Cognition in Schizophrenia: reliability, sensitivity, and comparison with a standard neurocognitive battery. *Schizophrenia research*. 2004 Jun1;68(2-3):283-97.

26 Velligan DI, Bow-Thomas CC. Executive function in schizophrenia. InSeminars in clinical neuropsychiatry 1999 Jan1(Vol. 4, No. 1, pp. 24-33).

27 Kravariti E, Dixon T, Frith C, et al. Association of symptoms and executive function in schizophrenia and bipolar disorder. *Schizophrenia research*. 2005 May1;74(2-3):221-31.

28 Lee J, Park S. Working memory impairments in schizophrenia: a meta-analysis. *Journal of abnormal psychology*. 2005 Nov;114(4):599.

29 Forbes NF, Carrick LA, McIntosh AM, et al. Working memory in schizophrenia: a meta-analysis. *Psychological medicine*. 2009 Jun1;39(6):889.

30 Flashman LA, Flaum M, Gupta S, et al. Soft signs and neuropsychological performance in schizophrenia. *The American journal of psychiatry*. 1996 Apr.

31 Bruder GE, Wexler BE, Sage MM, et al. Verbal memory in schizophrenia: additional evidence of subtypes having different cognitive deficits. *Schizophrenia Research*. 2004 Jun1;68(2-3):137-47.

32 Hill SK, Beers SR, Kmiec JA, et al. Impairment of verbal memory and learning in antipsychotic-naıve patients with first-episode schizophrenia. *Schizophrenia research*. 2004 Jun1;68(2-3):127-36.

33 Bokat CE, Goldberg TE. Letter and category fluency in schizophrenic patients: a meta-analysis. *Schizophrenia research*. 2003 Nov1;64(1):73-8.

34 Ojeda N, Sánchez P, Peña J, et al. Verbal fluency in schizophrenia: does cognitive performance reflect the same underlying mechanisms in patients and healthy controls?. *The Journal of nervous and mental disease.* 2010 Apr1;198(4):286-91.

35 Harris JG, Minassian A, Perry W. Stability of attention deficits in schizophrenia. *Schizophrenia research*. 2007 Mar1;91(1-3):107-11.

36 Carter JD, Bizzell J, Kim C, et al. Attention deficits in schizophrenia—preliminary evidence of dissociable transient and sustained deficits. *Schizophrenia research*. 2010 Sep1;122(1-3):104-12.

For peer review only - http://bmjopen.bmj.com/site/about/guidelines.xhtml

37 Rose D, Papoulias C, MacCabe J, Walke J. Service users' and carers' views on research towards stratified medicine in psychiatry: a qualitative study. *BMC research notes.* 2015 Dec;8(1):1-9.

38 American Psychiatric Association. Diagnostic and statistical manual of mental disorders (DSM-5®). American Psychiatric Pub; 2013 May 22.

39 Egerton A, Murphy A, Donocik J, et al. Dopamine and Glutamate in Antipsychotic-Responsive Compared With Antipsychotic-Nonresponsive Psychosis: A Multicenter Positron Emission Tomography and Magnetic Resonance Spectroscopy Study (STRATA). *Schizophrenia Bulletin*. 2020 Sep10.

40 Kemp R, Hayward P, Applewhaite G, et al. Compliance therapy in psychotic patients: randomised controlled trial. *Bmj.* 1996 Feb10;312(7027):345-9.

41 Kemp R, Kirov G, Everitt B, et al. Randomised controlled trial of compliance therapy. *The British Journal of Psychiatry*. 1998 May1;172(5):413-9.

42 Guy W. ECDEU assessment manual for psychopharmacology. US Department of Health, Education, and Welfare, Public Health Service, Alcohol, Drug Abuse, and Mental Health Administration, National Institute of Mental Health, Psychopharmacology Research Branch, Division of Extramural Research Programs; 1976.

43 Kay SR, Fiszbein A, Opler LA. The positive and negative syndrome scale (PANSS) for schizophrenia. *Schizophrenia bulletin.* 1987;13(2):261.

44 Sheehan DV, Lecrubier Y, Sheehan KH, et al. The Mini-International Neuropsychiatric Interview (MINI): the development and validation of a structured diagnostic psychiatric interview for DSM-IV and ICD-10. *Journal of clinical psychiatry*. 1998 Jan1;59(20):22-33.

45 Opler LA, Kay SR, Lindenmayer JP, et al. Structured clinical interview: The positive and negative syndrome scale (SCI-PANSS). North Tonawanda, NY: Multi-Health Systems. 1999.

46 Keefe RS, Harvey PD, Goldberg TE, et al. Norms and standardization of the Brief Assessment of Cognition in Schizophrenia (BACS). *Schizophrenia research.* 2008 Jul1;102(1-3):108-15.

47 StataCorp LL. Stata Statistical Software: Release 15 College Station, TX, 2017. www. stata. com/features/documentation/(last accessed on 1 March 2018). 2017.

48 Craik FI, Bialystok E. Cognition through the lifespan: mechanisms of change. *Trends in cognitive sciences*. 2006 Mar1;10(3):131-8.

For peer review only - http://bmjopen.bmj.com/site/about/guidelines.xhtml

49 Harvey PD. What is the evidence for changes in cognition and functioning over the lifespan in patients with schizophrenia?. *The Journal of clinical psychiatry.* 2014 Jun5;75(suppl 2):34-8.

50 Perlick D, Mattis S, Stastny P, et al. Gender differences in cognition in schizophrenia. *Schizophrenia Research.* 1992 Oct1;8(1):69-73.

51 Han M, Huang XF, Xiu MH, et al. Gender differences in cognitive function of patients with chronic schizophrenia. *Progress in neuro-psychopharmacology and biological psychiatry*. 2012 Dec3;39(2):358-63.

52 Cuesta MJ, Peralta V, Zarzuela A. Illness duration and neuropsychological impairments in schizophrenia. *Schizophrenia research*. 1998 Oct.

53 Bagney A, Rodriguez-Jimenez R, Martinez-Gras I, Sanchez-Morla EM, Santos JL, Jimenez-Arriero MA, Lobo A, McGorry PD, Palomo T. Negative symptoms and executive function in schizophrenia: does their relationship change with illness duration?. *Psychopathology*. 2013;46(4):241-8.

54 Bulmer MG. Principles of statistics. Courier Corporation; 1979.

55 Lawrie SM, Ingle GT, Santosh CG, et al. Magnetic resonance imaging and single photon emission tomography in treatment-responsive and treatment-resistant schizophrenia. *The British Journal of Psychiatry.* 1995 Aug1;167(2):202-10.

56 Smith RC, Kadewari RP, Rosenberger JR, et al. Nonresponding schizophrenia: differentiation by neurological soft signs and neuropsychological tests. *Schizophrenia bulletin.* 1999 Jan1;25(4):813-25.

57 Joober R, Rouleau GA, Lal S, et al. Neuropsychological impairments in neuroleptic-responder vs.-nonresponder schizophrenic patients and healthy volunteers. *Schizophrenia Research.* 2002 Jan15;53(3):229-38.

58 de Bartolomeis A, Balletta R, Giordano S, et al. Differential cognitive performances between schizophrenic responders and non-responders to antipsychotics: correlation with course of the illness, psychopathology, attitude to the treatment and antipsychotics doses. *Psychiatry research.* 2013 Dec15;210(2):387-95.

59 Bourque J, Lakis N, Champagne J, et al. Clozapine and visuospatial processing in treatment-resistant schizophrenia. *Cognitive Neuropsychiatry.* 2013 Nov1;18(6):615-30.

60 Frydecka D, Beszłej JA, Gościmski P, et al. Profiling cognitive impairment in treatment-resistant schizophrenia patients. *Psychiatry research.* 2016 Jan30;235:133-8.

For peer review only - http://bmjopen.bmj.com/site/about/guidelines.xhtml

61 Kravariti E, Demjaha A, Zanelli J, et al. Neuropsychological function at first episode in treatment-resistant psychosis: findings from the ÆSOP-10 study. *Psychological medicine.* 2019 Sep;49(12):2100-10.

62 Huang J, Zhu Y, Fan F, et al. Hippocampus and cognitive domain deficits in treatment-resistant schizophrenia: A comparison with matched treatment-responsive patients and healthy controls☆,☆☆,★,★★. *Psychiatry Research: Neuroimaging.* 2020 Mar30;297:111043.

63 Iasevoli F, Avagliano C, Altavilla B, et al. Evaluation of a few discrete clinical markers may predict categorization of actively symptomatic non-acute schizophrenia patients as treatment resistant or responders: a study by ROC curve analysis and multivariate analyses. *Psychiatry research.* 2018 Nov1;269:481-93.

64 Sosin D, Ivashchenko D, Sozaeva Z, et al. Cognitive impairment in patients with treatment resistant schizophrenia: Associations with DRD2, DRD3, HTR2A, BDNF and CYP2D6 genetic polymorphisms. *Neurology, Psychiatry and Brain Research.* 2019 Sep1;33:48-55.

65 Kellendonk C, Simpson EH, Polan HJ, Malleret G, Vronskaya S, Winiger V, Moore H, Kandel ER. Transient and selective overexpression of dopamine D2 receptors in the striatum causes persistent abnormalities in prefrontal cortex functioning. *Neuron*. 2006 Feb 16;49(4):603-15.

66 Voce A, Calabria B, Burns R, Castle D, McKetin R. A systematic review of the symptom profile and course of methamphetamine-associated psychosis: substance use and misuse. *Substance use & misuse*. 2019 Mar 21;54(4):549-59.

67 Miller EK, Cohen JD. An integrative theory of prefrontal cortex function. *Annual review of neuroscience*. 2001 Mar;24(1):167-202.

68. Schoonover KE, Dienel SJ, Lewis DA. Prefrontal cortical alterations of glutamate and GABA neurotransmission in schizophrenia: Insights for rational biomarker development. *Biomarkers in Neuropsychiatry*. 2020 Dec 1;3:100015.

69 Bojesen KB, Broberg BV, Fagerlund B, Jessen K, Thomas MB, Sigvard A, Tangmose K, Nielsen MØ, Andersen GS, Larsson HB, Edden RA. Associations between cognitive function and levels of glutamatergic metabolites and gamma-aminobutyric acid in antipsychotic-naïve patients with schizophrenia or psychosis. *Biological psychiatry*. 2021 Feb 1;89(3):278-87.

70 Arnsten AF. Catecholamine modulation of prefrontal cortical cognitive function. *Trends in cognitive sciences*. 1998 Nov 1;2(11):436-47.

For peer review only - http://bmjopen.bmj.com/site/about/guidelines.xhtml

71 Chan SK, Chan HY, Honer WG, et al. Predictors of Treatment-Resistant and Clozapine-Resistant Schizophrenia: A 12-Year Follow-up Study of First-Episode Schizophrenia-Spectrum Disorders. *Schizophrenia Bulletin.* 2020 Oct12.

72 Zanelli J, Mollon J, Sandin S, et al. Cognitive change in schizophrenia and other psychoses in the decade following the first episode. *American Journal of Psychiatry.* 2019 Oct1;176(10):811-9.

73 Barder HE, Sundet K, Rund B, et al. Ten year neurocognitive trajectories in first-episode psychosis. *Frontiers in human neuroscience.* 2013 Oct7;7:643.

74 Sommer IE, Tiihonen J, van Mourik A, Tanskanen A, Taipale H. The clinical course of schizophrenia in women and men—a nation-wide cohort study. NPJ schizophrenia. 2020 May 1;6(1):1-7.

75 Ochoa S, Usall J, Cobo J, Labad X, Kulkarni J. Gender differences in schizophrenia and first-episode psychosis: a comprehensive literature review. Schizophrenia research and treatment. 2012 Oct;2012.

76 Bak N, Ebdrup BH, Oranje B, Fagerlund B, Jensen MH, Düring SW, Nielsen MØ, Glenthøj BY, Hansen LK. Two subgroups of antipsychotic-naive, first-episode schizophrenia patients identified with a Gaussian mixture model on cognition and electrophysiology. *Translational psychiatry*. 2017 Apr;7(4):e1087-.

77 Ebdrup BH, Axelsen MC, Bak N, Fagerlund B, Oranje B, Raghava JM, Nielsen MØ, Rostrup E, Hansen LK, Glenthøj BY. Accuracy of diagnostic classification algorithms using cognitive-, electrophysiological-, and neuroanatomical data in antipsychotic-naïve schizophrenia patients. *Psychological medicine*. 2019 Dec;49(16):2754-63.

For peer review only - http://bmjopen.bmj.com/site/about/guidelines.xhtml

Supplementary material
Table S.1
Univariable and multivariable linear regression models for response status and BACS performance

	R	NR	Unadjusted				Adjusted for age, gender, illness duration and anticholinergic effects				Adjusted for anticholinergic effects			
BACS measure	N	N	β	SE	95%CI	P-value	β	SE	95%CI	P-value	β	SE	95%CI	P-value
Verbal Memory	53	50	-1.99	2.34	-6.63 ; 2.66	.398	-3.18	2.38	-7.90 ; 1.54	.185	-2.34	2.35	-7.00 ; 2.32	.322
Digit Sequencing	53	50	0.11	0.90	-1.67 ; 1.89	.901	0.07	0.92	-1.76 ; 1.89	.944	-0.02	0.90	-1.81 ; 1.77	.983
Verbal Fluency	53	50	1.23	1.86	-2.46 ; 4.91	.510	1.08	1.94	-2.78 ; 4.94	.580	1.17	1.88	-2.56 ; 4.90	.536
Token Motor	53	49	-0.42	2.95	-6.28 ; 5.43	.886	-1.40	2.97	-7.29 ; 4.50	.638	-0.62	2.99	-6.56 ; 5.31	.835
Symbol Coding	53	50	-1.84	2.28	-6.37 ; 2.68	.421	-1.89	2.37	-6.60 ; 2.83	.428	-2.04	2.30	-6.60 ; 2.53	.378
Tower of London	53	50	0.40	0.82	-1.23 ; 2.03	.625	0.35	0.84	-1.30 ; 2.01	.672	0.23	0.82	-1.40 ; 1.85	.782
z score composite	53	49	-0.03	0.29	-0.60 ; 0.54	.922	-0.08	0.30	-0.68 ; 0.52	.798	-0.07	0.29	-0.65 ; 0.50	.800
t score composite	53	49	-0.64	2.87	-6.32 ; 5.05	.825	-1.33	3.02	-7.32 ; 4.67	.662	-1.24	2.88	-6.96 ; 4.48	.668

Note. R = antipsychotic responder; NR = antipsychotic non-responder; BACS = Brief Assessment of Cognition in Schizophrenia; CIs = confidence intervals.

For peer review only

STRATA: Investigating factors associated with response to antipsychotic treatment
Protocol. Version 4.0, 19th August 2016
REC REF: 15/LO/0038

STRATA: Investigating factors associated with response to antipsychotic treatment

Sponsor

Name of organisation: King's College London
Address: Room 1.8 Hodgkin Building, Guy's Campus, London SE1 4UL
Telephone: 020 7848 6960
Fax:
Email: keith.brennan@kcl.ac.uk

Chief Investigator

Name: Dr James MacCabe
Address: Institute of Psychiatry, Psychology & Neuroscience (IoPPN), De Crespigny Park, SE5 8AF
Telephone: 02078480757
Fax:
Email: james.maccabe@kcl.ac.uk

Name and address of Principal Investigator (on grant award).

Name: Prof Shitij Kapur
Address: Institute of Psychiatry, Psychology & Neuroscience (IoPPN), De Crespigny Park, SE5 8AF
Telephone: 020 7848 0593
Fax:
Email: shitij.kapur@kcl.ac.uk

Name and address of lead Co-Investigators at sites

Name: Prof Shon Lewis
Address: The University of Manchester, University Place, Oxford Road, Manchester, M13 9PL
Telephone: 0161 306 7944
Fax:
Email: shon.lewis@manchester.ac.uk

Name: Prof Mick O'Donovan
Address: MRC Centre for Neuropsychiatric Genetics and Genomics, Cardiff University, Henry Wellcome Building, Heath Park, Cardiff, CF14 4XN
Telephone: (029) 20687066
Fax:
Email: odonovanmc@cardiff.ac.uk

For peer review only - http://bmjopen.bmj.com/site/about/guidelines.xhtml

STRATA: Investigating factors associated with response to antipsychotic treatment
Protocol. Version 4.0, 19th August 2016
REC REF: 15/LO/0038

Name: Prof Stephen Lawrie
Address: University of Edinburgh, Royal Edinburgh Hospital, Edinburgh, EH10 5HF
Telephone: 0131 537 6671
Fax:
Email: s.lawrie@ed.ac.uk

For peer review only

For peer review only - http://bmjopen.bmj.com/site/about/guidelines.xhtml

STRATA: Investigating factors associated with response to antipsychotic treatment
Protocol. Version 4.0, 19th August 2016
REC REF: 15/LO/0038

CONTENTS

1. Introduction

2. Study Objectives and Design

2.1. Study Objectives

2.2 Study Design

3. Sample Size, Statistics, Selection and Withdrawal of Subjects

4. Study procedures

5. Sample Handling and Laboratories

6. Assessment of Safety

7. Study oversight arrangements

8. Ethics and Regulatory Approvals

9. Data Handling

10. Finance & Publication Policy

For peer review only

For peer review only - http://bmjopen.bmj.com/site/about/guidelines.xhtml

STRATA: Investigating factors associated with response to antipsychotic treatment
Protocol. Version 4.0, 19th August 2016
REC REF: 15/LO/0038

Study Synopsis

Full Title	Investigating factors associated with response to antipsychotic treatment
Short Title/Acronym	STRATA
Protocol Version number and Date	Version 4.0, 19th August 2016
Study Duration	36 months
Study Design	Basic Science
Sponsor/Co-sponsors	Kings College London / South London and Maudsley NHS Foundation Trust
Chief Investigator	Dr James MacCabe
REC number	15/LO/0038
Primary objective	The principle objective is to use Positron Emission Tomography (PET), Magnetic Resonance Spectroscopy (MRS), genetic and clinical data to confirm recent evidence of a distinct subtype of schizophrenia, based on differences in dopamine and glutamate function which would lead to developing a clinically useful, acceptable and cost-effective stratification tool.
Secondary objective (s)	To establish a lasting network of academia and industry partners and patients databases to facilitate and expedite both follow-up and novel research built to address patient stratification.<br><br>To examine and improve test-re-test and intercentre reliability of neuroimaging procedures for future large scale multisite studies in this mould that will be conducted.
Number of Subject	100
Main Inclusion Criteria	aged 18-65;<br><br>DSM 5 schizophrenia/schizophreniform disorder.

For peer review only - http://bmjopen.bmj.com/site/about/guidelines.xhtml

STRATA: Investigating factors associated with response to antipsychotic treatment
Protocol. Version 4.0, 19th August 2016
REC REF: 15/LO/0038

Statistical Methodology and Analysis	Summary statistics will be used to describe the demographic and clinical characteristics of each participant group.<br><br>Group differences in demographic, clinical variables and 18F-DOPAKi and glutamate concentration will be determined using pre-specified between group comparisons as appropriate (e.g. Chi square; Fischer's Exact; ANOVA).

For peer review only

For peer review only - http://bmjopen.bmj.com/site/about/guidelines.xhtml

STRATA: Investigating factors associated with response to antipsychotic treatment
Protocol. Version 4.0, 19th August 2016
REC REF: 15/LO/0038

1. Introduction

People with schizophrenia suffer from a range of symptoms including hallucinations (such as hearing voices), delusions (false beliefs) and thought disorder (thoughts not flowing in a logical way), as well as 'negative symptoms' such as a lack of motivation and withdrawal from social contact. Currently, antipsychotic medication is the mainstay of treatment for schizophrenia and all existing antipsychotic medications are thought to work by acting to reduce transmission of a brain chemical called dopamine. However, even after attempts to treat the disorder with two different antipsychotics, around 30% of patients still fail to improve. When this happens, the medical guidelines recommend treatment with a different drug called clozapine. However clozapine has several side effects and requires regular blood tests, so people do not like taking it. It is also ineffective in some patients.

The result is that a large number of patients spend too long on ineffective drugs which impact greatly on their mental health, well-being and quality of life whilst the cost of ineffective treatment is a huge financial burden to the NHS, consuming 25-50% of the total national mental health budget.

STRATA (funded by a £5M Medical Research Council award) aims to build on new evidence from neuroimaging and genetics studies suggesting that those who do not respond may actually have a completely different neurochemical abnormality causing their symptoms (the same sort of symptoms as are caused by excessive dopamine), involving a different chemical called glutamate. There are some new medicines under development that we hope will help people whose illness has not responded to standard medicines acting on dopamine.

We aim to develop a method to predict, even as early as when first seen, which patients will respond to standard dopamine drugs, and which people are instead more likely to respond to the new glutamate drugs. This will allow people to receive the medicines they need straight away, without having to try ineffective drugs first.

The proposed research programme is broken down into several parts. This protocol describes the first study, which is a UK, multicentre study using brain scans to confirm that those patients who don't respond to standard treatments have higher glutamate levels, but lower dopamine levels than those who respond well. This information, along with clinical and genetic information, will be used to develop tests to identify in advance which people will respond to dopaminergic versus glutamatergic medication.

2. Study Objectives and Design

2.1. Study Objectives and Outcomes

The principle objective is to use Positron Emission Tomography (PET), Magnetic Resonance Spectroscopy (MRS), genetic and clinical data to confirm recent evidence of a distinct subtype of schizophrenia, based on differences in dopamine and glutamate function which would lead to developing a clinically useful, acceptable and cost-effective stratification tool.

The secondary research objectives are:

For peer review only - http://bmjopen.bmj.com/site/about/guidelines.xhtml

i) To establish a lasting network of academia and industry partners and patient databases to facilitate and expedite both follow-up and novel research built to address patient stratification.

ii) To examine and improve test-re-test and intercentre reliability of neuroimaging procedures for future large scale multisite studies in this mould that will be conducted.

The study is designed to generate a predictive test for treatment response so the outcome will be the overall measure generated. The data that will lead to this will include MRS glutamate level, the PET Ki value, polygenic risk score and clinical variables such as PANSS score.

2.2 Study Design

STRATA is a multi centred study. 100 participants will be recruited across 4 university research sites including KCL, University of Manchester, Cardiff University, and University of Edinburgh.

Participants will consent to all aspects of the study including interviews/assessments, blood and urine sampling, MRI scan and PET scan (the latter in London and Manchester only) but can also choose to opt out of some tasks if necessary.

1. Assessments

An initial interview will collect demographic and personal information (e.g. address, contact details, date of birth, gender, handedness, head injury and other relevant medical history), and structured assessments of medication history and response. Clinical information will also be recorded from medical records. The Mini International Neuropsychiatric Interview (MINI) will be used to confirm diagnosis, which takes around 15 minutes to complete.

Illness severity will be measured using:

i. Positive and Negative Syndrome Scale (PANSS),
ii. Clinical Global Impression scale (CGI -SCH)
iii. Kemp Clinician Rating Scale (of adherence to treatment)
iv. Brief Assessment of Cognition in Schizophrenia (BACS)

2. Biological samples

Blood samples will be collected via cannula (as described under the PET scan section below) or by venous puncture, during a routine blood sample whenever possible. The participant will give up to 50ml in blood (around 3 tablespoons), this is in line with sampling guidelines.

While the biological sample collection is ongoing, samples will be stored at the laboratory corresponding to each research site. The samples for genetics analysis will subsequently be transferred to the MRC Center for Neuropsychiatric Genetics and Genomics, Cardiff University.

KCL/South London and the Maudsley (SLaM) participants will also be invited to participate in the BRC Biobank. This is covered by a separate ethical approval (09/H0606/84 NRES Committee South Central-Oxford C.)

For peer review only

For peer review only - http://bmjopen.bmj.com/site/about/guidelines.xhtml

STRATA: Investigating factors associated with response to antipsychotic treatment
Protocol. Version 4.0, 19th August 2016
REC REF: 15/LO/0038

Participants will be asked to provide samples (urine and blood) for metabolomics analysis. This will be processed at MRC-NIHR National Phenome Centre. The Centre is funded by the MRC and NIHR and led by Imperial College London and King's College London.

As of April 2016, participants will also have a sample taken for proteomic analysis. These samples will be sent to the University of Manchester (Molecular Pathology Innovation Centre). This will be within the 50ml sampling guideline already approved.

3. Magnetic Resonance Imaging (MRI)

The MRI scans (100 in total) will take place at four locations (London, Cardiff, Manchester and Edinburgh) at NHS Trust or University sites. The MRI session will last a maximum of 1 hour. During the scan, participants will be asked to lie flat on their back with their head inside the scanner. The scanner makes a loud noise as it takes pictures, so participants will be given headphones to wear and asked to lie as still as possible. The researcher will be able to speak to the participant over the microphone throughout and participants will be told if they do feel uncomfortable the session can be stopped at any time. The MRI scan itself is painless and safe. Some people find scans claustrophobic or anxiety-provoking, and we have a mock scanner that participants can try out first. The scanner consists of a powerful magnet, which may attract metal objects. Therefore before the scan participants will go through a safety questionnaire, to check that they can have the scan. If a participant has any metal in their body, either from accidents or operations, they may not be able to have the MRI scan, but they can still take part in the rest of the study.

All data collection will occur at 3 Tesla. During the scan, data acquisition will include acquisition of localizer, T1-weighted and T2-weighted structural scans. 1H-MRS data for measurement of regional concentrations of glutamate and other metabolites present in the 1H-MRS spectra will be acquired using conventional PRESS (Point RESolved Spectrocopy) acquisition routines, as well as a resting state fMRI sequence if time allows.

Due to change of scanner at Cardiff University, participants recruited in Cardiff prior to the decommissioning of the old scanner will be re-contacted and asked whether they would volunteer for a second MRI scan on the new scanner. They will also be asked to repeat some of the interview/assessments and may be asked for biological samples (only in circumstances where these were not provided previously). Participants will be reimbursed for their time at the same rate.

In the unlikely event that MRI scanner issues or excessive movement make the MRS data unusable at other sites, participants can be re-contacted and asked whether they would like to volunteer for a second scan.

4. Positron Emission Tomography (PET)

The PET scans (60 in total, subset of those having MRI scans) will take place at two sites:
i) Imanova Limited, Imperial College London, Hammersmith Hospital in London.
ii)The Wolfson Molecular Imaging Centre in Manchester.

PET with the radiotracer 18F-DOPA will be used to assess brain dopaminergic function in a sub-set of participants (N=60) recruited at KCL and University of Manchester. The PET scan procedure involves an initial transmission scan followed by a dynamic scan lasting approximately

For peer review only - http://bmjopen.bmj.com/site/about/guidelines.xhtml

90 minutes after injection of the radiotracer 18F DOPA through a cannula inserted into an arm vein. In the event the participant has to get off the scanner e.g. to go to the toilet or for some other reason then the transmission scan may be repeated to reposition them in the camera. In the unlikely event of technical failure prior to or during the PET scan the subject will be invited back for a replacement session (the total dose will then be ~7.5mSv, and the risks of this will be explained).

In Manchester, participants will be offered the option of having an extra, High Resolution Research Tomograph (HRRT) scan after their main STRATA PET scan (after a 15 minute comfort break). This will be between 30-60 minutes depending upon participant tolerability. Due to the long half-life of 18F and the slow removal of 18F from the brain, this extra scan will not involve any further injection of radiotracer. Another transmission scan will be carried out for attenuation correction purposes although this will be of very low radiation dose (0.02mSv). In the event of significant head movement during the HRRT scan, this transmission scan may be repeated.

In order to minimise the peripheral breakdown of 18F-DOPA, an oral dose of 150mg carbidopa and 400mg entacapone will be given one hour prior to the scan. Very few people experience any side-effects from these. Very occasionally people experience stomach upset, muscle movements, dry mouth and/or an orange tinge to their urine from the tablets, which may last a few hours to a day. This permits the use of a lower dose of 18F-DOPA than would otherwise be necessary. Participants will be asked to refrain from eating, drinking (apart from water) and smoking from midnight on the night before the scan, until after the scan is finished. This is because large amino acids may affect brain uptake of 18F-DOPA. Participants will also be instructed not to take illicit drugs (such as cannabis or cocaine) in the prior three days. Before the scan we will ask for a urine sample to check whether substances that can affect the scan are in their system. Women of childbearing age will have a pregnancy test and will be required to use regular contraception prior to the scan. At the start of the scan we will give participants a radiotracer (which is mildly radioactive) to measure the brain dopamine system. At the end of the scan the cannula will be removed from their arm.

Participants taking part in a PET scan at Imanova (SLaM/KCL participants) will have an additional 1-2 tablespoons (up to 30ml) of blood taken through their cannula to measure natural blood chemicals (hormones and genes) that are connected to dopamine function.

Participants taking part in a PET scan in Manchester will have all their bloods taken at this point (up to 50ml) as described under 'Biological Samples Section', whenever possible.

3. Sample Size, Statistics, Selection and Withdrawal of Subjects

The patients will be identified by members of the clinical team. Only the clinical team (who may also be part of the research team with NHS honorary contracts) will be able to access participant records and data prior to consent. No patient records will be screened by study researchers prior to consent. Study researchers will have access to patient records after/ if participants have consented to this.

We will recruit a total of 100 participants. Potential participants may be referred via clinical teams or other research studies/existing databases with consent to re-contact or registries and recruitment initiatives in NHS Trusts whose terms are in accordance with NHS Trust policies.

For peer review only - http://bmjopen.bmj.com/site/about/guidelines.xhtml

STRATA: Investigating factors associated with response to antipsychotic treatment
Protocol. Version 4.0, 19th August 2016
REC REF: 15/LO/0038

Participants will be first approached by a member of the clinical team or a member of the research team, with approval from the clinical team/ other study

Inclusion Criteria

1) aged 18-65;
2) DSM 5 schizophrenia/schizophreniform disorder.
3) Participants must read and write in English at a level sufficient to understand and complete study-related procedures

Exclusion Criteria

1) Pregnancy;
2) Severe head injury involving loss of consciousness >5 minutes (ever);
3) Meeting ICD criteria for harmful substance misuse or psychotic disorder secondary to substance misuse;
4) Participation in MRI scans requires exclusion of contraindications to MRI at 3 tesla e.g. metallic or electronic implants;
5) Severe claustrophobia.
6) Treatment with clozapine in the last 3 months

To establish and confirm the stratifier 1H-MRS data will be acquired in a total of 100 patients early in the course of their treatment; 50 T-Resp and 50 T-NonResp; matched for chronicity of illness.

Operational definition of T-Resp:
(i) treatment with only one antipsychotic drug since onset, or treatment changes have been due to adverse effects, not for non-response. (ii) CGI-SCH severity score of <4; (iii) PANSS total <60 (Leucht 2005); (iv) CRS >3

Operational definition of T-NonResp:
(i) documented treatment with at least two antipsychotics each above the minimum therapeutic dose as defined by the BNF for >4 weeks each; (ii) despite ongoing treatment and adequate adherence (assessed by iv) a CGI-SCH severity score of >3; (iii). PANSS total severity rating of at least 70 iv) Clinician Rating Scale (CRS; a measure of adherence) (Kemp et al 1996) >3.

Power and sample size calculation:
The study is powered to give >95% power to detect differences found in Egerton et al 2012 (α=0.05, allowing for 10% loss of sensitivity due to combining data from multiple centres). We have more than 80% power to detect a significant difference between a ROC curve with AUC 0.7 and chance, assuming α=0.05, 2-tailed. Two-tailed 18F-DOPA PET data will be acquired in a subset at 2 sites (N=60) to determine if the double dissociation between DA function and GLU function we have seen in chronic patients is also evident early in the illness course, where the strategy is most likely to be used (T-Resp n=30, T-NonResp n=30; powered to give >95% power to detect differences found in Demjaha et al 2012; α=0.05, allowing for 10% loss of sensitivity due to combining data from multiple centres).

Summary statistics will be used to describe the demographic and clinical characteristics of each participant group.

For peer review only - http://bmjopen.bmj.com/site/about/guidelines.xhtml

STRATA: Investigating factors associated with response to antipsychotic treatment
Protocol. Version 4.0, 19th August 2016
REC REF: 15/LO/0038

Group differences in demographic, clinical variables and 18F-DOPA Ki and glutamate concentration will be determined using pre-specified between group comparisons as appropriate (e.g. Chi square; Fischer's Exact; ANOVA).

Missing data will be minimal given that data is being collected prospectively. The exact reason for the missing data will be recorded. Any blank measures or spurious data will be checked against the paper copy of the CRF stored securely at sites.

Participants will be clearly told they can withdraw from the study at any time without having to give a reason. This is clear in the information sheet and the researcher will also explain this verbally to participants during the informed consent process. If a participant wishes to withdraw from a study all their identifiable data will be destroyed. Data or tissue already collected with the consent, which is not identifiable, would be retained and used in the study. No further data or tissue would be collected or any other research procedures carried out on or in relation to the participant.

Control group

We will recruit up to 15 healthy volunteers aged 18-65 to be scanned at each PET site (two sites; Imanova Limited, Imperial College London and The Wolfson Molecular Imaging Centre in Manchester) and 10 healthy volunteers aged 18-65 to be scanned at each MRI site (4 sites). This is to determine inter-site scanner variability and to provide normal range data for comparison with the clinical groups. In addition to the exclusion criteria above, healthy volunteers will be excluded if there is a history of schizophrenia or other psychotic disorder. Healthy volunteers will be recruited using an existing database of interested potential participants held at KCL.

4. Study procedures

Informed Consent

1) Participants will be first approached by a member of the clinical team or a member of the research team, with approval from the clinical team.
2) The study will be described verbally to potential participants and they will be given a copy of the information sheet. They will be encouraged to ask questions about the research. Potential participants will be allowed as much time as they require to make a decision and at least 24 hours so they are able to seek advice from others about participation, including previous participants in the research where possible.
3) If a patient expresses an interest in taking part, capacity to consent will be assessed and documented by the research team, in consultation with the clinical team.
4) If the patient has capacity to consent and agrees to participate in the study, they will be asked to sign and date two copies of the consent form. One copy will be kept by the participant and one by the research team. The research team will pass onto the clinical team to scan into medical notes, or incorporate in paper notes.
5) The participant will be informed that they can withdraw consent at any time, and without giving a reason.
6) Participants will be informed they are to be compensated for their time and travel expenses. This monetary amount will be up to £120 (£145 in Manchester) depending on which parts of the study the participant is involved with.

For peer review only - http://bmjopen.bmj.com/site/about/guidelines.xhtml

STRATA: Investigating factors associated with response to antipsychotic treatment
Protocol. Version 4.0, 19th August 2016
REC REF: 15/LO/0038

Within Avon and Wiltshire Mental Health Partnership NHS Trust, Everyone Included will be used to identify potentially eligible participants. Potentially eligible participants are identified based on the study inclusion /exclusion criteria, excluding those who have declined to receive information. This is done via an automated search of the Trust's electronic patient record system (RiO). An authorised search will be requested by a member or the R&D department, who are part of the clinical team and carried out by a member of the Information Analysis team. A data set is returned directly to the Everyone Included Administrators for processing the letters. No patient identifiable data will ever leave the Trust or be accessed by an external research team during this process.

The 'Research Opportunity Letter' will be sent to these individuals. The letter itself will not contain any patient identifiable or disclosing information (such as making reference to their diagnosis or medications). It will provide a free-post return slip and contact details (phone, email, website, postal address) inviting individuals to get in touch if they would like to further information / to take part. The onus is on the individual to express an interest, otherwise no further action is taken.

Upon responding to the 'Research Opportunity Letter', a Participant Information Sheet will be provided. If the research team is external, the individual will be asked if they are happy for their details (i.e. name and phone number) to be passed directly to the research team. No information is ever accessed by or passed to an external research team without first gaining permission from the potential participant. At this point standard study recruitment processes proceed.

Risks and burdens

The questionnaires involve personal questions and recalling experiences that some people may find distressing. Participants will be told if they feel uncomfortable with any of the questions they do not have to answer them.

Blood sampling and placing the cannula can cause some discomfort, and there is a possibility that a small bruise may develop. This task will be performed by research workers trained in phlebotomy. Any risks of infection will be contained by using standard sterile procedures and the risks associated with this task will be the same as for any other blood sample collection.

Any participants who become distressed during any procedure involved in this study will be encouraged to pause and will be reminded routinely that they can withdraw from the study at any time without a reason or penalty.

Any clinically significant issues that may arise during the assessment, the verbal consent will be obtained from the patient to pass onto the responsible psychiatrist or other relevant member of the staff. This will always be done with the participants' permission and will only be breached in the rare cases when there is judged to be an issue of safety, for example if the participant makes specific threats towards an individual.

Imaging

The MRI and PET scans themselves are painless and safe. Some people find the scans claustrophobic or anxiety-provoking. There is a mock scanner that participants can try out first if they wish. Participants will be told if they feel uncomfortable the scanning can be stopped at any time. Before the scan we will go through a safety questionnaire, to check that participants can have the scan. If they have any metal in their body, either from accidents or operations, they may

For peer review only - http://bmjopen.bmj.com/site/about/guidelines.xhtml

not be able to have the MRI scan, but they can still take part in the rest of the study. Clinical Research workers and research workers will log screening results.

Very occasionally people experience side effects from the medication they receive when taking part in a PET scan. These side-effects can include stomach upsets, muscle movements, dry mouth and/or an orange tinge to their urine. These side-effects may last a few hours to a day but participants will be warned about this and told it is nothing to worry about.

PET scans involve a small amount of radiation. Any exposure to radiation carries a risk of damaging the body's tissues and possibly triggering cancer at a later date. However, the risk is very small. A standard PET scan in this study will expose participants to 3.7mSv, (this may be 3.72mSv in Manchester if participants decide to have the extra, high resolution PET scan), which is the same amount of radiation that they are exposed to from natural sources of radiation, such as the sun, over the course of 18 months. In extremely rare cases the PET scan may need to be repeated and we have ARSAC approval for a maximum of 7.5mSv exposure per participant. Most experts believe that the risk of cancer developing only becomes significant in people who are exposed to 100mSv or more. However, as a precaution we are excluding pregnant or breastfeeding women. A pregnancy test will be carried out on female urine samples before the PET scan is conducted. Participants will be asked to consent to this on the consent form. Clinical Research workers and research workers will log screening results and ensure participants will not be exposed to more than 10mSv in 12 months (ARSAC guidelines suggest 10mSv as the normal upper limit for radiation exposure related to research procedure)

5. Sample handling and laboratories

Biological sample collection tubes and barcodes will be sent to sites in advance from the MRC Center for Neuropsychiatric Genetics and Genomics, Cardiff University. Samples will be stored in laboratories at sites and transportation will be organized when required (likely at 6 monthly basis, dependent on recruitment). Details of sample collection and storage at site will be recorded. Study SOPs will describe collection and storage specifications to ensure all sites are following the same guidelines.

When samples arrive at Cardiff University, researchers will ensure that the physical integrity of these samples have not been compromised in transit and track the samples in using their barcodes. The research team at Cardiff will notify the sponsor and the other study teams of any issues in transportation.

Cardiff University will extract DNA from the blood. We will perform genome-wide and targeted genotyping and/or exome or whole genome sequencing. We will seek genetic association with the imaging and other outcome measures at the level of individual genotype/sequence variant, genes, gene sets/pathways and polygenic or other summary scores.

A urine and blood sample will undergo metabolomic analysis at MRC National Phenome Centre. The Centre is funded by the MRC and NIHR and led by Imperial College London and King's College London. An additional blood sample will undergo proteomic analysis at the University of Manchester.

KCL/South London and the Maudsley (SLaM) participants will also be invited to participate in the BRC Biobank. This is covered by a separate ethical approval (09/H0606/84 NRES Committee South Central-Oxford C.)

For peer review only - http://bmjopen.bmj.com/site/about/guidelines.xhtml

STRATA: Investigating factors associated with response to antipsychotic treatment
Protocol. Version 4.0, 19th August 2016
REC REF: 15/LO/0038

6. Assessment of Safety

There are no serious adverse events expected to occur during the study.

All blood samples will only be taken by researchers trained in phlebotomy. All risks are the same as for any routine blood sample and are therefore minimal.

The drugs administered and the radiotracer used for the PET are standard procedures. The drugs administered may cause stomach upsets, muscle movements, dry mouth and/or an orange tinge to their urine. These side-effects may last a few hours to a day but participants will be warned about this and told it is nothing to worry about. Female participants will have a pregnancy test in advance.

For MRI scans a safety questionnaire will be carried out prior to the scan to check the participant does not have any metal in their bodies from operations or accidents.

7. Study oversight arrangements

STRATA is a multi-centred study and this will be managed by attendance at a monthly Consortium Executive meeting which will be responsible for the effective oversight of the daily activities of the study. Quarterly Consortium Board (CB) teleconferences will oversee the progress of, and interaction between, the workstreams to maintain communication of issues and progress between sites across the different aspects of STRATA. The CB will submit six-monthly Programme reports to the funder, MRC.

The project team consists of a full time Project Manager based at the IoPPN, KCL and a 50% Project Manager at the University of Manchester.

8. Ethics & Regulatory Approvals

REC name and address: South East Coast-Surrey Research Ethics Committee, Whitefriars, Level 3, Block B, Lewins Mead, Bristol, BS1 2NT

This study has also been reviewed and approved by the Administration of Radioactive Substances Advisory Committee (ARSAC).

9. Data Handling

Once participants have consented to be in the study some personal details will be taken. These details will be taken by the researcher with full consent to do so. These details will be kept securely at sites and used to contact patients when required to make appointments. No personal data will be shared with anyone outside of that study team. Each participant will be given a unique identifier and any clinical or genetic or imagining data relating to the same participant will link via that code.

Data will be entered and stored on a secure web application called Research Electronic Data Capture (REDCap). REDCap will not store any personal details and all participants will have a unique non-identifiable ID code. This unique ID code will then be used to merge all processed imaging, genetics and clinical data. REDCap will be hosted on secure servers at the Biomedical

For peer review only - http://bmjopen.bmj.com/site/about/guidelines.xhtml

Research Centre at Kings College London. All sites can access REDCap for the purposes of data entry via a web browser and data is uploaded when a WIFI signal is available.

The CI will ensure that the trial is conducted in compliance with the principles of the Declaration of Helsinki (1996), and in accordance with all applicable regulatory requirements including but not limited to the Research Governance Framework, Trust and Research Office policies and procedures and any subsequent amendments.

10. Finance and Publication Policy

STRATA is funded by a £ £4,900,000 Medical Research Council grant. Kings College London will receive and manage this funding. A collaboration agreement has agreed budgets between sites.

Analysis and findings from the study will be published as papers in journals. No identifiable data will be included.

This study has been adopted onto the UKCRN Portfolio and the research project will be registered on their database which is publicly available.

For peer review only - http://bmjopen.bmj.com/site/about/guidelines.xhtml

STROBE Statement—checklist of items that should be included in reports of observational studies

	Item No	Recommendation	Noted on pg.
**Title and abstract**	1	(*a*) Indicate the study's design with a commonly used term in the title or the abstract	1,2
		(*b*) Provide in the abstract an informative and balanced summary of what was done and what was found	1,2
**Introduction**			
Background/rationale	2	Explain the scientific background and rationale for the investigation being reported	4
Objectives	3	State specific objectives, including any prespecified hypotheses	5
**Methods**			
Study design	4	Present key elements of study design early in the paper	5
Setting	5	Describe the setting, locations, and relevant dates, including periods of recruitment, exposure, follow-up, and data collection	5
Participants	6	(*a*) *Cohort study*—Give the eligibility criteria, and the sources and methods of selection of participants. Describe methods of follow-up<br>*Case-control study*—Give the eligibility criteria, and the sources and methods of case ascertainment and control selection. Give the rationale for the choice of cases and controls<br>*Cross-sectional study*—Give the eligibility criteria, and the sources and methods of selection of participants	6,7
		(*b*) *Cohort study*—For matched studies, give matching criteria and number of exposed and unexposed<br>*Case-control study*—For matched studies, give matching criteria and the number of controls per case	N/A
Variables	7	Clearly define all outcomes, exposures, predictors, potential confounders, and effect modifiers. Give diagnostic criteria, if applicable	7,8
Data sources/ measurement	8*	For each variable of interest, give sources of data and details of methods of assessment (measurement). Describe comparability of assessment methods if there is more than one group	7,8
Bias	9	Describe any efforts to address potential sources of bias	8
Study size	10	Explain how the study size was arrived at	13
Quantitative variables	11	Explain how quantitative variables were handled in the analyses. If applicable, describe which groupings were chosen and why	7,8
Statistical methods	12	(*a*) Describe all statistical methods, including those used to control for confounding	8
		(*b*) Describe any methods used to examine subgroups and interactions	8
		(*c*) Explain how missing data were addressed	N/A
		(*d*) *Cohort study*—If applicable, explain how loss to follow-up was addressed<br>*Case-control study*—If applicable, explain how matching of cases and controls was addressed<br>*Cross-sectional study*—If applicable, describe analytical methods taking account of sampling strategy	N/A
		(*e*) Describe any sensitivity analyses	8

For peer review only - http://bmjopen.bmj.com/site/about/guidelines.xhtml

Continued on next page

For peer review only

For peer review only - http://bmjopen.bmj.com/site/about/guidelines.xhtml

**Results**			
Participants	13*	(a) Report numbers of individuals at each stage of study—eg numbers potentially eligible, examined for eligibility, confirmed eligible, included in the study, completing follow-up, and analysed	6,7,8
		(b) Give reasons for non-participation at each stage	6
		(c) Consider use of a flow diagram	N/A
Descriptive data	14*	(a) Give characteristics of study participants (eg demographic, clinical, social) and information on exposures and potential confounders	8,9
		(b) Indicate number of participants with missing data for each variable of interest	N/A
		(c) *Cohort study*—Summarise follow-up time (eg, average and total amount)	N/A
Outcome data	15*	*Cohort study*—Report numbers of outcome events or summary measures over time	N/A
		Case-control study—Report numbers in each exposure category, or summary measures of exposure	N/A
		Cross-sectional study—Report numbers of outcome events or summary measures	10,11
Main results	16	(*a*) Give unadjusted estimates and, if applicable, confounder-adjusted estimates and their precision (eg, 95% confidence interval). Make clear which confounders were adjusted for and why they were included	11
		(*b*) Report category boundaries when continuous variables were categorized	N/A
		(*c*) If relevant, consider translating estimates of relative risk into absolute risk for a meaningful time period	N/A
Other analyses	17	Report other analyses done—eg analyses of subgroups and interactions, and sensitivity analyses	Supplementary
**Discussion**			
Key results	18	Summarise key results with reference to study objectives	12
Limitations	19	Discuss limitations of the study, taking into account sources of potential bias or imprecision. Discuss both direction and magnitude of any potential bias	12, 13, 14
Interpretation	20	Give a cautious overall interpretation of results considering objectives, limitations, multiplicity of analyses, results from similar studies, and other relevant evidence	12, 13, 14
Generalisability	21	Discuss the generalisability (external validity) of the study results	13
**Other information**			
Funding	22	Give the source of funding and the role of the funders for the present study and, if applicable, for the original study on which the present article is based	15

*Give information separately for cases and controls in case-control studies and, if applicable, for exposed and unexposed groups in cohort and cross-sectional studies.

Note: An Explanation and Elaboration article discusses each checklist item and gives methodological background and published examples of transparent reporting. The STROBE checklist is best used in conjunction with this article (freely available on the Web sites of PLoS Medicine at http://www.plosmedicine.org/, Annals of Internal Medicine at http://www.annals.org/, and Epidemiology at http://www.epidem.com/). Information on the STROBE Initiative is available at www.strobe-statement.org.

For peer review only - http://bmjopen.bmj.com/site/about/guidelines.xhtml
